# Review of the genus *Namadytes* Hesse, 1969 (Insecta: Diptera: Mydidae: Syllegomydinae)

**DOI:** 10.3897/BDJ.2.e1071

**Published:** 2014-03-10

**Authors:** Torsten Dikow, Stephanie Leon

**Affiliations:** † National Museum of Natural History, Smithsonian Institution, Washington, DC, United States of America; ‡ Department of Entomology, University of California at Riverside, Riverside, United States of America

**Keywords:** Diptera, Mydidae, Syllegomydinae, *
Namadytes
*, Afrotropical Region, taxonomy

## Abstract

The Mydidae genus *Namadytes* Hesse, 1969 is reviewed. It is known from five species, primarily occurring in Namibia. The study of newly available material from both Namibia and South Africa deposited in several natural history collections results in the recognition of three species and new synonymy of two, *i.e.*, *Namadytes pallidus* Hesse, 1972 is a new junior synonym of *Namadytes maculiventris* (Hesse, 1969) and *Namadytes prozeskyi* Hesse, 1969: 282 is a new junior synonym of *Namadytes vansoni* Hesse, 1969: 280. All three species are re-described and comments on sexual dimorphism and intraspecific variation are made, a dichotomous key for their identification is presented, and illustrations and photographs are provided to support the descriptions and facilitate future identification. Distribution, occurrence in biodiversity hotspots *sensu* Conservation International, and seasonal incidence with associated weather and climatic data are discussed for all species. A morphological structure ventral to the halter and posterior to the metathoracic spiracle, the infra-halter sclerite, is here newly termed.

## Introduction

The southern African Mydidae fauna is the most diverse world-wide both in terms of species numbers and generic diversity. The seminal work by Hesse (1969) on the southern African mydids based primarily on specimens he collected himself throughout western South Africa, in which he described no fewer than 108 new species (106 of which are still valid) and 12 new genera (11 of which are still valid), provided a comprehensive overview of this unique fauna. Hesse (1972) added to the knowledge following the examination of additional material from Namibia (then South-West Africa).

### Taxonomic history

At the start of this review, *Namadytes* Hesse, 1969 is known from five species with an interesting taxonomic history.

- Hesse (1969) described the genus *Namadytes* (p. 278) based on two female specimens and representing two distinct species, *i.e.*, *Namadytes vansoni* Hesse, 1969: 280 from Seeheim, Namibia and *Namadytes prozeskyi* Hesse, 1969: 282 from Arechadamab, Namibia. On page 284, Hesse describes the genus *Namamydas* Hesse, 1969 based on a single male specimen, identified as *Namamydas maculiventris* Hesse, 1969, collected by himself and his colleagues from the South African Museum (now Iziko South African Museum) at Vioolsdrift on the South African bank of the Orange River, which represents the border with Namibia. Hesse comments on the unique arrangement of the male aedeagal prongs being fused medially in this species.
- Hesse (1972) established the synonymy of *Namadytes* and *Namamydas* based on the collection of female and male specimens at the Excelsior farm No. 127, Namibia, of a new species, *Namadytes cimbebasiensis* Hesse, 1972. He writes (p. 139), "The discovery of two additional species of *Namadytes* from South West Africa, described below, and of which one is represented by both sexes, proves without doubt that the male sex of *Namadytes* (unknown at the time of description) is identical generically with the male described by me as *Namamydas*. The latter genus thus falls away as a synonym of *Namadytes*."
- Bowden (1980) cataloged all five valid *Namadytes* species.

### Goals of this review

As can be seen from the above information, *Namadytes* and its five species were represented by nine specimens prior to this study. Two species, *i.e.*, *Namadytes prozeskyi* and *Namadytes vansoni*, were known from females only, while *Namadytes maculiventris* and *Namadytes pallidus* were known only from males, and *Namadytes cimbebasiensis* from both sexes.

This review is based on an additional 61 specimens from numerous natural history collections accumulated over the past 35 years representing all previously known species. Such an increase in specimen number, their geographic occurrence expanding the range of the genus considerably, and substantial morphological variation suggested that a few new species might be represented among the material. However, this not the case and to the contrary the number of valid species is reduced to three by synonymy.

## Materials and methods

Morphological terminology and abbreviations for setae follows McAlpine (1981), Stuckenberg (1999), Cumming and Wood (2009), Dikow (2009) except for the term 'aedeagal epimere', which is used as described by Hesse (1969) on page 32. Abdominal tergites are abbreviated in the descriptions with 'T', and sternites are abbreviated with 'S'. The terms prothoracic, mesothoracic, and metathoracic are abbreviated 'pro', 'mes', and 'met', respectively. The term pubescence (adjective 'pubescent') refers to the short, fine microtrichia densely covering certain body parts. Other generalized terms follow the Torre-Bueno Glossary of Entomology Nichols (1989). Species descriptions are based on composites of all specimens and not exclusively on the holotype and are compiled from a character matrix of 149 features and 224 character states assembled with Lucid Builder (version 3.5) and eventually exported as natural language descriptions. These species descriptions have been deposited in the DRYAD data depository and can be accessed in XML-format following the SDD (Structure of Descriptive Data) standard. When available, species are fully described in the male sex while females are only described with those features that differ. The structure of terminalia is only described once for the genus except when species differ. Additional species-specific features of the male terminalia should be interpreted from the provided illustrations.

The female genitalia and male terminalia are first excised and macerated in 10% potassium hydroxide (KOH) at 55 °C followed by neutralization in acetic acid (CH_3_COOH) and rinsing in distilled water (H_2_O). They are temporarily stored in 75% ethanol (C_2_H_5_OH) for examination and illustration and eventually sealed in polyethylene vials containing 100% glycerine (C_3_H_8_O_3_) and attached to the specimen's pin. Morphological features were examined using a Zeiss SteREO Discovery.V12 stereo microscope. Illustrations were observed with a *camera lucida*, drawn, inked, and scanned. The setation on terminalia is not shown. Wing length is measured from the tegula to the distal tip of the wing. Whole habitus photographs of pinned specimens were taken using a Visionary Digital Passport II system (base and StackShot only), an Olympus E-30 digital SLR, a 50 mm macro lens (equivalent to 100 mm focal length in 35 mm photography), and a 25 mm extension tube. The specimens were illuminated by a Falcon FLDM-i200 LED dome-light for even and soft light. Adobe DNG-format images were stacked using HeliconFocus software. Photographs of particular features were taken on a Zeiss SteREO Discovery.V12 stereo microscope and an attached Olympus PEN E-PL5 digital camera. All specimen photographs have been deposited in Morphbank:: Biological Imaging. These images can be automatically harvested by the Encyclopedia of Life (EOL) and are available under the respective species page.

The following data on species occurrences are given (where available): country, state/province, county, locality, geographic co-ordinates (formatted in both decimal and degrees minutes seconds latitude/longitude), elevation (in meters), date of collection (format: yyyy-mm-dd), habitat information, sampling protocol (if other than hand netting), collector, catalog number (a unique specimen number and any other identifying number), depository (institution and collection code), number of specimens and sex, life stage, name of person who identified the specimen, and any other previous identifications (note that for synonymized species the holotype still retains its status as a primary type specimen and therefore the particular material examined list will include two (or more) holotypes; see the entry under 'previousIdentifications' for the original identification by the author). Each specimen is listed with a unique specimen number (either an institutional catalog number or an AAM-XXXXXX number used by the senior author) that will allow the re-investigation as well as provide a unique Life Science Identifier (LSID). The occurrence of all species is illustrated in distribution maps plotted with SimpleMappr (Shorthouse 2010) with all of those localities for which co-ordinates are available. Type localities are plotted with a square symbol while all other specimens are plotted with a circular symbol. The distribution map includes Biodiversity Hotspots *sensu* Conservation International and the electronic shape-files were obtained from: http://www.conservation.org/where/priority_areas/hotspots/Pages/hotspots_main.aspx. The specimen occurrence data are deposited as a Darwin Core Archive (DwC-A) in the Global Biodiversity Information Facility (GBIF). The dichotomous, interactive key has been build with Lucid Phoenix and the multi-access, matrix-based key with Lucid Builder and both have been deposited in the IdentifyLife archive, registered in Lucidcentral, and made available on the senior author's research web-site. All taxon names have been registered in ZooBank (Pyle and Michel 2008).

### Institutions providing specimens

Institutions providing specimens are listed below, together with the abbreviations used in the text when citing depositories (institutionCode), a link to the record in the Global Registry of Biodiversity Repositories (GRBio), and the people who kindly assisted: AMGS – Albany Museum, Grahamstown, Eastern Cape, South Africa (A. Kirk-Spriggs, S. Gess); BMNH – The Natural History Museum, London, UK (E. McAlister); CSCA – California State Collection of Arthropods, Sacramento, California, USA (M. Hauser); INHS – Illinois Natural History Survey, Urbana-Champaign, Illinois, USA (D. Webb, M. Irwin); NMNW – National Museum of Namibia, Windhoek, Khomas, Namibia (A. Kirk-Spriggs); NMSA – KwaZulu-Natal Museum, Pietermaritzburg, KwaZulu-Natal, South Africa (B. Muller); SAMC – Iziko South African Museum, Cape Town, Western Cape, South Africa (M. Cochrane); SANC – South African National Collection of Insects, Pretoria, Gauteng, South Africa (R. Urban); TMSA – Ditsong National Museum of Natural History (formerly Transvaal Museum), Pretoria, Gauteng, South Africa (M. Krüger); USNM – National Museum of Natural History, Smithsonian Institution, Washington, DC, USA.

## Data resources

**Figure 1.**
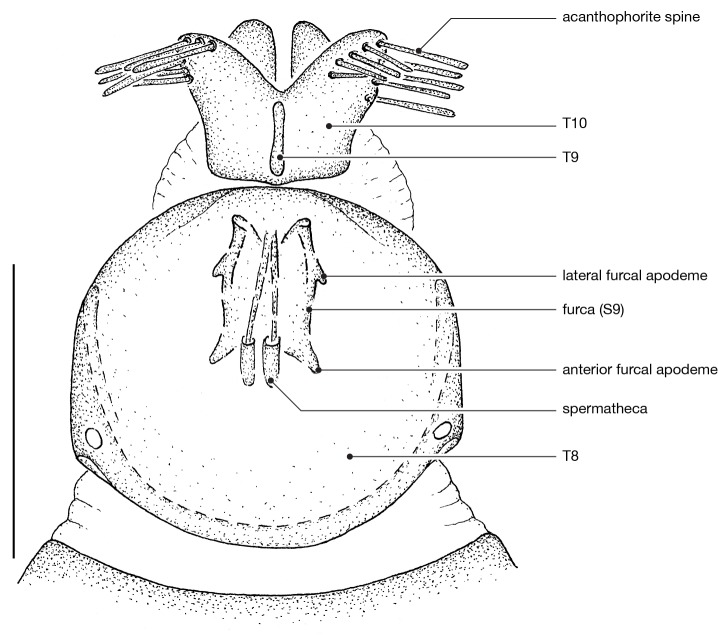
*Namadytes cimbebasiensis* ♀ terminalia (NMNW-H7809), dorsal view showing position of internal furca and spermathecae. Scale line = 1 mm.

- DRYAD: natural-language species descriptions from Lucid Builder in SDD format (also available as Suppl. material 1).
- GBIF: specimen occurrence data – 5e6acf4c-e913-45fd-8466-5c0b92c322dd.
- Morphbank:: Biological Imaging: high-resolution photographs – 835218.
- SimpleMappr: distribution map – 2368 (as in figure 1) and in Google Earth as a KML file.
- Lucid Builder: illustrated, multi-entry, matrix-based identification key – asilodflies.si.edu and IdentifyLife.
- Lucid Phoenix: illustrated, dichotomous identification key – asilodflies.si.edu.

## Taxon treatments

### Namadytes

Hesse, 1969

urn:lsid:zoobank.org:act:528203A4-E5FB-46E5-A5DF-811EDE021F18

Namadytes Hesse, 1969: 278.
Namamydas Hesse, 1969: 284 junior synonym *sensu*Hesse 1972: 139 (ZooBank LSID).
Namadytes Namadytes vansoni Hesse, 1969 – Hesse 1969: 278. ; Bowden 1980: 331. catalog

#### Description

**Female genitalia** (Fig. 1): densely arranged anteriorly directed setae present on T7–8 and S7–8; T8 with broad anterior rectangular apodeme; T9 formed by wide, rectangular sclerite with median protuberance; T9+10 entirely fused, T10 divided into 2 heavily sclerotized acanthophorite plates, 8–10 acanthophorite spines per plate; 2 spermathecae, all equally large, formed by ± expanded heavily sclerotized ducts; individual spermathecal duct short; S9 (furca) formed by 1 sclerite, ring–like (joined anteriorly and posteriorly), anterior furcal apodeme present, 2 lateral projections forming divided apodeme, lateral furcal apodeme present, median furcal bridge absent.

**Male terminalia** (e.g., Fig. 2) T1–7 well-developed, entirely sclerotized, T8 postero-medially weakly sclerotized, with anterior transverse sclerotized bridge connecting lateral sclerites; T7–8 anteriorly with 2 lateral apodemes; S6 regular, without any special setation postero-medially, S8 simple plate, entire (undivided) ventro-medially, not fused to T8 dorso-laterally; epandrium formed by single sclerite (fused medially ± entirely), distally in dorsal view blunt, evenly rounded; subepandrial sclerite without lateral or median protuberances; hypandrium strongly concave, cup-shaped, entirely sclerotized ventrally (forming a single sclerite), entirely fused with gonocoxite, forming a gonocoxite-hypandrial complex, supra-hypandrial sclerite absent; gonocoxite dorso-ventrally flattened in distal ½, higher in proximal ½, without median or lateral protuberance, gonocoxal apodeme absent; 2 functional aedeagal prongs, short and wide, medio-distally connected; aedeagal epimere absent; lateral ejaculatory process present, large cylindrical sclerite; ejaculatory apodeme formed by single dorso-ventrally oriented plate; ventro-median margin of dorsal aedeagal sheath heavily sclerotized (appearing entirely closed); dorsal aedeagal sheath long, sperm sac entirely covered; sperm sac appearing ± heavily sclerotized.

#### Diagnosis

**Figure 3.**
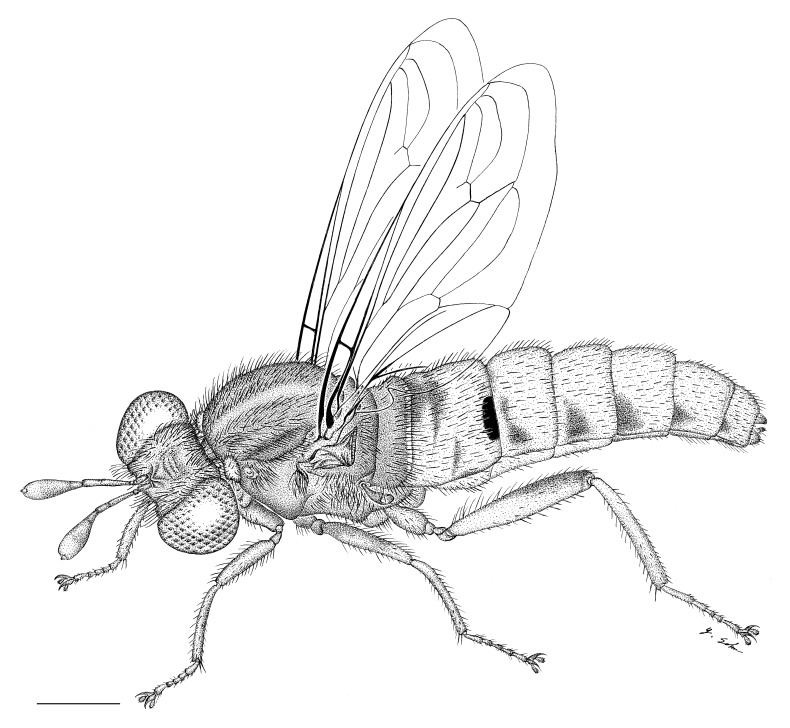
*Namadytes vansoni* male (AAM-002988). Scale line = 2 mm.

The genus (Fig. 3) is distinguished from other Syllegomydinae by the structures of the male genitalia (aedeagal prongs fused medially), the presence of a V-shaped indentation on the dorso-median antepronotum, and the presence of a tuft of setae on the infra-halter sclerite (ventral to halter base and posterior to metathoracic spiracle), with the exception of females of one species. Furthermore, flies are relatively small with a wing length of 6.6–14.2 mm and the males exhibit a yellow to light brown abdomen, which is unusual for southern African Mydidae.

#### Distribution

**Figure 4.**
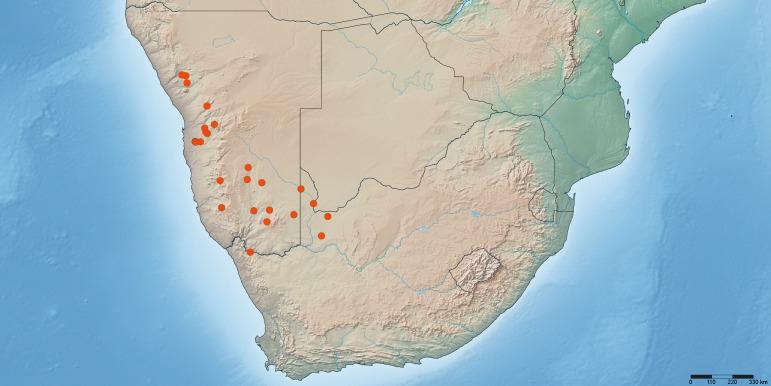
Distribution of *Namadytes*.

*Namadytes* is distributed in southern Africa and restricted to Namibia and north-westernmost South Africa (Fig. 4).

#### Taxon discussion

Males are unique in the arrangement of their medially fused aedeagal prongs and the yellow to light brown abdominal coloration. Females in contrast are more generalized and similar to other female Mydidae occurring in southern Africa. However, the antero-median V-shaped indentation on the antepronotum and the presence of white setae on the infra-halter sclerite are relatively easy to observe and distinguish the females from other Mydidae. There is considerable sexual dimorphism and the setation, for example on the anatergites, is always easier to observe in males. Intra-specific variation in the abdominal coloration, especially in females, is likewise substantial, which probably led Hesse to describe a species twice.

### Namadytes cimbebasiensis

Hesse, 1972

urn:lsid:zoobank.org:act:5AA365A2-2479-4959-BF33-45C64E746A1F

Namadytes cimbebasiensis Hesse, 1972: 143.

#### Materials

- **Type status:** Holotype. **Occurrence:** catalogNumber: SAM-DIP-A007146; recordedBy: H. Brown; sex: 1 male; lifeStage: Adult; otherCatalogNumbers: AAM-000450; **Taxon:** scientificNameID: urn:lsid:zoobank.org:act:5AA365A2-2479-4959-BF33-45C64E746A1F; scientificName: *Namadytes cimbebasiensis* Hesse, 1972; family: Mydidae; genus: Namadytes; specificEpithet: *cimbebasiensis*; scientificNameAuthorship: Hesse, 1972; **Location:** country: Namibia; stateProvince: Hardap; county: Maltahöhe; locality: Excelsior No. 127; verbatimCoordinates: 25°24'00''S 016°12'00''E; decimalLatitude: -25.4; decimalLongitude: 16.2; **Identification:** identifiedBy: A. Hesse; dateIdentified: 1972; **Event:** eventDate: 1969-05-07; **Record Level:** institutionCode: SAMC; collectionCode: Insects; basisOfRecord: PreservedSpecimen
- **Type status:** Paratype. **Occurrence:** catalogNumber: SAM-DIP-A007146; recordedBy: H. Brown; sex: 1 female; lifeStage: Adult; otherCatalogNumbers: AAM-000451; **Taxon:** scientificNameID: urn:lsid:zoobank.org:act:5AA365A2-2479-4959-BF33-45C64E746A1F; scientificName: *Namadytes cimbebasiensis* Hesse, 1972; family: Mydidae; genus: Namadytes; specificEpithet: cimbebasiensis; scientificNameAuthorship: Hesse, 1972; **Location:** country: Namibia; stateProvince: Hardap; county: Maltahöhe; locality: Excelsior No. 127; verbatimCoordinates: 25°24'00''S 016°12'00''E; decimalLatitude: -25.4; decimalLongitude: 16.2; **Identification:** identifiedBy: A. Hesse; dateIdentified: 1972; **Event:** eventDate: 1969-05-07; **Record Level:** institutionCode: SAMC; collectionCode: Insects; basisOfRecord: PreservedSpecimen
- **Type status:** Paratype. **Occurrence:** catalogNumber: SAM-DIP-A007146; recordedBy: H. Brown; sex: 1 male; lifeStage: Adult; otherCatalogNumbers: AAM-000452; **Taxon:** scientificNameID: urn:lsid:zoobank.org:act:5AA365A2-2479-4959-BF33-45C64E746A1F; scientificName: *Namadytes cimbebasiensis* Hesse, 1972; family: Mydidae; genus: Namadytes; specificEpithet: cimbebasiensis; scientificNameAuthorship: Hesse, 1972; **Location:** country: Namibia; stateProvince: Hardap; county: Maltahöhe; locality: Excelsior No. 127; verbatimCoordinates: 25°24'00''S 016°12'00''E; decimalLatitude: -25.4; decimalLongitude: 16.2; **Identification:** identifiedBy: A. Hesse; dateIdentified: 1972; **Event:** eventDate: 1969-05-07; **Record Level:** institutionCode: SAMC; collectionCode: Insects; basisOfRecord: PreservedSpecimen
- **Type status:** Paratype. **Occurrence:** catalogNumber: SAM-DIP-A007146; recordedBy: H. Brown; sex: 1 male; lifeStage: Adult; otherCatalogNumbers: AAM-000453; **Taxon:** scientificNameID: urn:lsid:zoobank.org:act:5AA365A2-2479-4959-BF33-45C64E746A1F; scientificName: *Namadytes cimbebasiensis* Hesse, 1972; family: Mydidae; genus: Namadytes; specificEpithet: cimbebasiensis; scientificNameAuthorship: Hesse, 1972; **Location:** country: Namibia; stateProvince: Hardap; county: Maltahöhe; locality: Excelsior No. 127; verbatimCoordinates: 25°24'00''S 016°12'00''E; decimalLatitude: -25.4; decimalLongitude: 16.2; **Identification:** identifiedBy: A. Hesse; dateIdentified: 1972; **Event:** eventDate: 1969-05-07; **Record Level:** institutionCode: SAMC; collectionCode: Insects; basisOfRecord: PreservedSpecimen
- **Type status:** Other material. **Occurrence:** catalogNumber: NMNW-H7809; sex: 1 female; lifeStage: Adult; otherCatalogNumbers: AAM-003014; **Taxon:** scientificNameID: urn:lsid:zoobank.org:act:5AA365A2-2479-4959-BF33-45C64E746A1F; scientificName: *Namadytes cimbebasiensis* Hesse, 1972; family: Mydidae; genus: Namadytes; specificEpithet: cimbebasiensis; scientificNameAuthorship: Hesse, 1972; **Location:** country: Namibia; stateProvince: Karas; county: Keetmanshoop; locality: Swartbaas West No. 276; verbatimCoordinates: 27°00'16''S 019°41'08''E; decimalLatitude: -27.00444; decimalLongitude: 19.68556; **Identification:** identifiedBy: T. Dikow S. Leon; dateIdentified: 2012; **Event:** eventDate: 1972-04-19–1972-04-22; **Record Level:** institutionCode: NMNW; collectionCode: Insects; basisOfRecord: PreservedSpecimen
- **Type status:** Other material. **Occurrence:** catalogNumber: NMNW-H7808; sex: 1 male; lifeStage: Adult; otherCatalogNumbers: AAM-003013; **Taxon:** scientificNameID: urn:lsid:zoobank.org:act:5AA365A2-2479-4959-BF33-45C64E746A1F; scientificName: *Namadytes cimbebasiensis* Hesse, 1972; family: Mydidae; genus: Namadytes; specificEpithet: cimbebasiensis; scientificNameAuthorship: Hesse, 1972; **Location:** country: Namibia; stateProvince: Karas; county: Keetmanshoop; locality: Swartbaas West No. 276; verbatimCoordinates: 27°00'16''S 019°41'08''E; decimalLatitude: -27.00444; decimalLongitude: 19.68556; **Identification:** identifiedBy: T. Dikow S. Leon; dateIdentified: 2012; **Event:** eventDate: 1972-04-19–1972-04-22; **Record Level:** institutionCode: NMNW; collectionCode: Insects; basisOfRecord: PreservedSpecimen
- **Type status:** Other material. **Occurrence:** catalogNumber: AAM-003000; recordedBy: M. Schwarz; sex: 1 female; lifeStage: Adult; otherCatalogNumbers: AAM-003000; **Taxon:** scientificNameID: urn:lsid:zoobank.org:act:5AA365A2-2479-4959-BF33-45C64E746A1F; scientificName: *Namadytes cimbebasiensis* Hesse, 1972; family: Mydidae; genus: Namadytes; specificEpithet: cimbebasiensis; scientificNameAuthorship: Hesse, 1972; **Location:** country: South Africa; stateProvince: Northern Cape; locality: Van Zylsrus, 90 km W; verbatimCoordinates: 27°04'47''S 021°17'08''E; decimalLatitude: -27.07972; decimalLongitude: 21.28556; **Identification:** identifiedBy: T. Dikow S. Leon; dateIdentified: 2012; **Event:** eventDate: 1990-03-26; **Record Level:** institutionCode: CSCA; collectionCode: Insects; basisOfRecord: PreservedSpecimen
- **Type status:** Other material. **Occurrence:** catalogNumber: AAM-003021; recordedBy: F. and S. Gess; sex: 1 male; lifeStage: Adult; otherCatalogNumbers: AAM-003021; **Taxon:** scientificNameID: urn:lsid:zoobank.org:act:5AA365A2-2479-4959-BF33-45C64E746A1F; scientificName: *Namadytes cimbebasiensis* Hesse, 1972; family: Mydidae; genus: Namadytes; specificEpithet: cimbebasiensis; scientificNameAuthorship: Hesse, 1972; **Location:** country: South Africa; stateProvince: Northern Cape; locality: 58 km N on R360 Upington-Kgalagadi; verbatimCoordinates: 27°59'22''S 020°59'51''E; decimalLatitude: -27.98944; decimalLongitude: 20.9975; **Identification:** identifiedBy: T. Dikow S. Leon; dateIdentified: 2012; **Event:** eventDate: 2000-04-06; **Record Level:** institutionCode: AMGS; collectionCode: Insects; basisOfRecord: PreservedSpecimen
- **Type status:** Other material. **Occurrence:** catalogNumber: NMSA-DIP-66615; recordedBy: J. Londt; sex: 1 male; lifeStage: Adult; **Taxon:** scientificNameID: urn:lsid:zoobank.org:act:5AA365A2-2479-4959-BF33-45C64E746A1F; scientificName: *Namadytes cimbebasiensis* Hesse, 1972; family: Mydidae; genus: Namadytes; specificEpithet: cimbebasiensis; scientificNameAuthorship: Hesse, 1972; **Location:** country: South Africa; stateProvince: Northern Cape; locality: Kgalagadi Transfontier Park, Kalahari Tented Camp; verbatimElevation: 947 m; verbatimCoordinates: 25°47'08''S 020°01'01''E; decimalLatitude: -25.78556; decimalLongitude: 20.01694; **Identification:** identifiedBy: T. Dikow; dateIdentified: 2013; **Event:** eventDate: 2012-04-11; habitat: dune scrub; **Record Level:** institutionCode: NMSA; collectionCode: Insects; basisOfRecord: PreservedSpecimen
- **Type status:** Other material. **Occurrence:** catalogNumber: NMSA-DIP-67240; recordedBy: J. and A. Londt; sex: 1 male; lifeStage: Adult; **Taxon:** scientificNameID: urn:lsid:zoobank.org:act:5AA365A2-2479-4959-BF33-45C64E746A1F; scientificName: *Namadytes cimbebasiensis* Hesse, 1972; family: Mydidae; genus: Namadytes; specificEpithet: cimbebasiensis; scientificNameAuthorship: Hesse, 1972; **Location:** country: South Africa; stateProvince: Northern Cape; locality: Kgalagadi Transfontier Park, Twee Rivieren; verbatimElevation: 864 m; verbatimCoordinates: 26°28'27''S 020°36'46''E; decimalLatitude: -26.47417; decimalLongitude: 20.61278; **Identification:** identifiedBy: T. Dikow; dateIdentified: 2013; **Event:** eventDate: 2012-04-08–2012-04-14; habitat: Acacia savanna; **Record Level:** institutionCode: NMSA; collectionCode: Insects; basisOfRecord: PreservedSpecimen

#### Description

**Male:** Fig. 5a, b.

Head: brown, in general grey pubescent; width distinctly greater than thorax, interocular distance on vertex larger than at ventral eye margin, vertex between compound eyes ± horizontally straight, medially only slightly below dorsal eye margin, parafacial area about as wide as ½ the width of central facial gibbosity; facial gibbosity distinct, well-developed and discernible in lateral view; mystax yellow, covering entire facial gibbosity; frons not elevated, predominantly apubescent; vertex entirely grey pubescent; postgena lightly grey pubescent; setation: vertex yellow, frons white or yellow, ocp setae white, pocl macrosetae absent; ocellar triangle apubescent; proboscis light brown, very short, vestigial, knob-like; labellum small, as wide as prementum, as long as prementum, unsclerotized laterally; maxillary palpus cylindrical, yellow, minute.

Antenna: brown, scape and pedicel white setose dorsally and ventrally; postpedicel cylindrical in proximal ½, symmetrically bulbous in distal ½, ≥ 4.0 times as long as combined length of scape and pedicel, asetose; apical seta-like sensory element situated apically in cavity on postpedicel.

Thorax: brown, lightly grey pubescent; scutum uniformly black or uniformly brown, surface entirely smooth, lightly grey pubescent, broad sublateral stripes apubescent, scutal setation comprised of scattered long white to yellow setae; dc setae pre- and postsuturally white or yellow, acr setae absent, lateral scutal setae white or yellow, npl setae 0, spal setae 0, pal setae 0; antepronotum dorso-medially with V-shaped indentation; postpronotal lobe light brown, grey pubescent; proepisternum, lateral postpronotum, and postpronotal lobe long white setose; scutellum grey pubescent, asetose, apical scutellar setae absent; mesopostnotum, anatergite, and katatergite partly grey pubescent, mesopostnotum apubescent, mesopostnotum asetose, anatergite asetose, katatergite long white setose; katatergite ± flat; anterior anepisternum white setose, supero-posterior anepisternum long white setose; posterior anepimeron asetose, katepimeron asetose; metanepisternum grey pubescent, asetose, metepimeron ± flat, yellow, grey pubescent, long white setose; infra-halter sclerite asetose or white setose.

Leg: light brown, setation yellow; pro, mes, and met coxa lightly white pubescent, short yellow setose; met trochanter setose medially; femur brown or light brown, met femur ± cylindrical only slightly wider than pro and mes femur, in distal ½ macrosetose, 1 antero-ventral and 1 postero-ventral row of macrosetae, postero-ventrally regular setose only; pro, mes, and met tibia straight, met tibia cylindrical, ventral keel absent, latero-posteriorly regular setose only; pro and mes tarsomere 1 longer than tarsomere 2, but less than combined length of tarsomeres 2–3, met tarsomere 1 as long as combined length of tarsomeres 2–4; pulvillus well-developed, as long as well-developed claw, and as wide as base of claw; empodium absent.

Wing: length = 6.6–8.1 mm; hyaline throughout, veins light brown, microtrichia absent; cells r_1_, r_4_, r_5_, m_3_, + cu*p* closed; C terminates at junction with R_1_; R_4_ terminates in R_1_; R_5_ terminates in R_1_; stump vein (R_3_) at base of R_4_ present, short not reaching R_2_; R_4_ and R_5_ widest apart medially; r-m distinct, R_4__+__5_ and M_1_ apart, connected by crossvein; M_1_ straight at r-m (not curving anteriorly), M_1_ (or M_1_+M_2_) terminates in R_1_; CuA_1_ and CuA_2_ split proximally to m-cu (cell m_3_ narrow proximally); M_3_+CuA_1_ do not terminate together in C; A_1_ undulating, cell a_1_ wide, A_1_ and wing margin further apart proximally than distally; alula well-developed; halter light yellow.

Abdomen: yellow to brown; setation comprised of dense white setae, surface entirely smooth; T1 brown, T2 brown anteriorly and postero-medially, otherwise yellow, T3–7 brown and yellow posteriorly; T1 and anterior ½ of T2 long white setose, remaining T short white setose; T predominantly apubescent; S1–7 light brown; S1 asetose, S2–7 sparsely white setose; S predominantly apubescent; T2–4 parallel-sided and not constricted waist-like; bullae on T2 black, transversely elongate, surface entirely smooth, T2 surface anterior to bullae smooth.

♂ terminalia: Fig. 2a, b, c.

**Female:** Fig. 5c, d.

Head: brown, facial gibbosity light brown; mystax white or yellow, sparsely covering entire facial gibbosity; setation: vertex white or yellow, pocl macrosetae light brown; maxillary palpus light brown.

Thorax: light brown, predominantly grey pubescent; scutum uniformly brown, predominantly brown pubescent, narrow sublateral stripes (wider anteriorly) and lateral and posterior margins grey pubescent, scutal setation comprised of scattered short yellow setae; proepisternum, lateral postpronotum, and postpronotal lobe short white setose; mesopostnotum, anatergite, and katatergite lightly grey pubescent, katatergite short white setose; anterior anepisternum white to yellow setose, supero-posterior anepisternum short white to yellow setose; metepimeron light brown; infra-halter sclerite asetose.

Leg: femur brown; pulvillus reduced, half length of well-developed claw.

Wing: length = 9.6–12.7 mm.

Abdomen: setation comprised of sparsely scattered short yellow setae; T1–7 brown, T2–6 light brown medially; T1–7 sparsely yellow setose; S1–7 brown; S1–7 sparsely short yellow setose; bullae on T2 oval.

♀ genitalia (Fig. 1): 8–10 acanthophorite spines per plate.

#### Diagnosis

This rather small species (wing length in males 6.6–8.1 mm and in females 9.6–12.7 mm) is distinguished from congeners by the entirely grey pubescent vertex, the short postpedicel (only about 4 times as long as combined length of scape and pedicel), the grey pubescent scutellum, and the few white setae on the infra-halter sclerite, which are absent in some females.

#### Distribution

**Figure 6.**
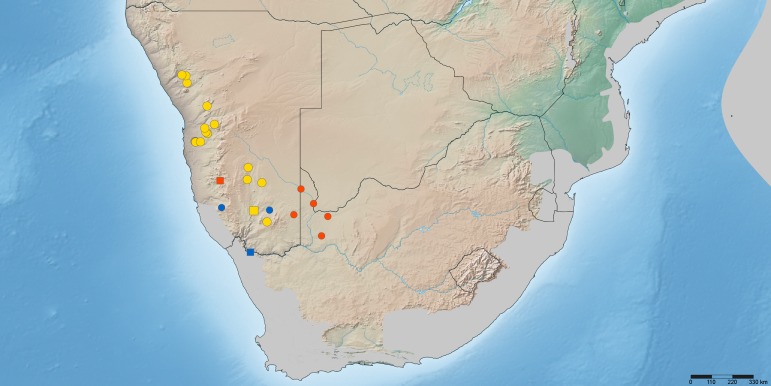
Map of southern Africa with elevational relief and biodiversity hotspots (in grey) showing distribution of *Namadytes cimbebasiensis* (red), *Namadytes maculiventris* (blue), and *Namadytes vansoni* (yellow). Map data available in Google Earth KML file and also through GBIF (data-set #5e6acf4c-e913-45fd-8466-5c0b92c322dd).

Namibia (Hardap, Karas) and South Africa (Northern Cape) (Fig. 6).

#### Biology

##### Habitat

*Namadytes cimbebasiensis* has recently been collected on dune scrub and in *Acacia* savanna in the Kgalagadi Transfrontier Park of the Kalahari Desert by J.G.H. Londt.

#### Discussion

*Namadytes cimbebasiensis* is very distinct and the smallest species of *Namadytes*. All specimens, originating from only five collecting events, are either greasy or are not in the best condition so that in particular the pubescence patterns might differ in freshly mounted material.

#### Type locality

Namibia: Hardab: Excelsior No. 127 (25°24'00''S, 016°12'00''E) (Fig. 6).

#### Biodiversity hotspot

Not known to occur in any of the southern African biodiversity hotspots (Cape Floristic Region, Maputaland-Pondoland-Albany, or Succulent Karoo) (Fig. 6).

### Namadytes maculiventris

(Hesse, 1969)

urn:lsid:zoobank.org:act:78BC184A-C090-4A75-9DDC-EF4CA95CB254

Namamydas maculiventris Hesse, 1969: 285.
Namadytes maculiventris (Hesse, 1969) new combination *sensu*Hesse 1972: 142.
Namadytes pallidus Hesse, 1972: 146 **syn. nov.** (ZooBank LSID). Type locality of Namadytes pallidus: Namibia: Karas: Keetmanshoop, 48 km SE (26°53'00''S 018°26'00''E).

#### Materials

- **Type status:** Holotype. **Occurrence:** catalogNumber: SAM-DIP-A007147; recordedBy: SAM Museum Staff; sex: 1 male; lifeStage: Adult; previousIdentifications: Namamydas maculiventris by A. Hesse in 1969; **Taxon:** scientificNameID: urn:lsid:zoobank.org:act:78BC184A-C090-4A75-9DDC-EF4CA95CB254; scientificName: *Namadytes maculiventris* Hesse, 1969; family: Mydidae; genus: Namadytes; specificEpithet: maculiventris; scientificNameAuthorship: Hesse, 1969; **Location:** country: South Africa; stateProvince: Northern Cape; locality: Vioolsdrift; verbatimCoordinates: 28°46'10''S 017°37'37''E; decimalLatitude: -28.76944; decimalLongitude: 17.62694; **Identification:** identifiedBy: A. Hesse; dateIdentified: 1972; **Event:** eventDate: 1935-03-00; **Record Level:** institutionCode: SAMC; collectionCode: Insects; basisOfRecord: PreservedSpecimen
- **Type status:** Holotype. **Occurrence:** catalogNumber: SAM-DIP-A007148; recordedBy: J. Rozen E. Martinez; sex: 1 male; lifeStage: Adult; otherCatalogNumbers: AAM-000454; previousIdentifications: Namadytes pallidus by A. Hesse in 1972; **Taxon:** scientificNameID: urn:lsid:zoobank.org:act:78BC184A-C090-4A75-9DDC-EF4CA95CB254; scientificName: *Namadytes maculiventris* Hesse, 1969; family: Mydidae; genus: Namadytes; specificEpithet: maculiventris; scientificNameAuthorship: Hesse, 1969; **Location:** country: Namibia; stateProvince: Karas; locality: Keetmanshoop, 48 km SE; verbatimCoordinates: 26°46'47''S 018°32'15''E; decimalLatitude: -26.77972; decimalLongitude: 18.5375; **Identification:** identifiedBy: T. Dikow S. Leon; dateIdentified: 2013; **Event:** eventDate: 1968-10-30; **Record Level:** institutionCode: SAMC; collectionCode: Insects; basisOfRecord: PreservedSpecimen
- **Type status:** Paratype. **Occurrence:** catalogNumber: SAM-DIP-A007148; recordedBy: J. Rozen E. Martinez; sex: 1 male; lifeStage: Adult; otherCatalogNumbers: AAM-000455; previousIdentifications: Namadytes pallidus by A. Hesse in 1972; **Taxon:** scientificNameID: urn:lsid:zoobank.org:act:78BC184A-C090-4A75-9DDC-EF4CA95CB254; scientificName: *Namadytes maculiventris* Hesse, 1969; family: Mydidae; genus: Namadytes; specificEpithet: maculiventris; scientificNameAuthorship: Hesse, 1969; **Location:** country: Namibia; stateProvince: Karas; locality: Keetmanshoop, 48 km SE; verbatimCoordinates: 26°46'47''S 018°32'15''E; decimalLatitude: -26.77972; decimalLongitude: 18.5375; **Identification:** identifiedBy: T. Dikow S. Leon; dateIdentified: 2013; **Event:** eventDate: 1968-10-30; **Record Level:** institutionCode: SAMC; collectionCode: Insects; basisOfRecord: PreservedSpecimen
- **Type status:** Other material. **Occurrence:** catalogNumber: USNMENT00779997; recordedBy: M. Schwarz; sex: 1 male; lifeStage: Adult; **Taxon:** scientificNameID: urn:lsid:zoobank.org:act:78BC184A-C090-4A75-9DDC-EF4CA95CB254; scientificName: *Namadytes maculiventris* Hesse, 1969; family: Mydidae; genus: Namadytes; specificEpithet: maculiventris; scientificNameAuthorship: Hesse, 1969; **Location:** country: Namibia; stateProvince: Karas; locality: Aus; verbatimCoordinates: 26°40'00''S 016°16'00''E; decimalLatitude: -26.66667; decimalLongitude: 16.26667; **Identification:** identifiedBy: T. Dikow S. Leon; dateIdentified: 2013; **Event:** eventDate: 1990-02-11; **Record Level:** institutionCode: USNM; collectionCode: Entomology; basisOfRecord: PreservedSpecimen

#### Description

**Male:** Fig. 7.

Head: brown, facial gibbosity light brown, in general densely grey pubescent; width distinctly greater than thorax, interocular distance on vertex larger than at ventral eye margin, vertex between compound eyes ± horizontally straight, medially only slightly below dorsal eye margin, parafacial area about as wide as ½ the width of central facial gibbosity; facial gibbosity distinct, well-developed and discernible in lateral view; mystax white, densely covering entire facial gibbosity; frons not elevated, predominantly apubescent; vertex entirely white pubescent; postgena white pubescent; setation: vertex white, frons white, ocp setae white, pocl macrosetae white; ocellar triangle apubescent; proboscis yellow, very short, vestigial, knob-like; labellum small, as wide as prementum, as long as prementum, unsclerotized laterally; maxillary palpus cylindrical, yellow, minute.

Antenna: brown, scape and pedicel white setose dorsally and ventrally; postpedicel cylindrical in proximal ½, symmetrically bulbous in distal ½, ≥ 7.0 times as long as combined length of scape and pedicel, asetose; apical seta-like sensory element situated apically in cavity on postpedicel.

Thorax: brown, lightly grey pubescent; scutum predominantly black, anteriorly and laterally yellow to light brown, surface entirely smooth, lightly grey pubescent, scutal setation comprised of long white setae with distinct rows of long dorsocentral setae and dense lateral scutal setae; dc setae pre- and postsuturally white, acr setae absent, lateral scutal setae white, npl setae 0, spal setae 0, pal setae 0; antepronotum dorso-medially with V-shaped indentation; postpronotal lobe yellow, white pubescent; proepisternum, lateral postpronotum, and postpronotal lobe long white setose; scutellum apubescent, asetose, apical scutellar setae absent; mesopostnotum, anatergite, and katatergite grey pubescent, mesopostnotum asetose, anatergite asetose, katatergite long white setose; katatergite ± flat; anterior anepisternum white setose, supero-posterior anepisternum long white setose; posterior anepimeron asetose, katepimeron asetose; metanepisternum grey pubescent, asetose, metepimeron ± flat, yellow, grey pubescent, long white setose; infra-halter sclerite white setose.

Leg: yellow to light brown, setation predominantly white; pro, mes, and met coxa lightly white pubescent, long white setose; met trochanter setose medially; femur yellow to light brown, met femur ± cylindrical only slightly wider than pro and mes femur, in distal ½ macrosetose, 1 antero-ventral and 1 postero-ventral row of macrosetae, postero-ventrally long white, erect setose with setae arranged in distinct row; pro, mes, and met tibia straight, met tibia cylindrical, ventral keel absent, latero-posteriorly long white, erect setose with setae arranged in distinct row; pro and mes tarsomere 1 longer than tarsomere 2, but less than combined length of tarsomeres 2–3, met tarsomere 1 as long as combined length of tarsomeres 2–4; pulvillus well-developed, as long as well-developed claw, and as wide as base of claw; empodium absent.

Wing: length = 9.6–12.2 mm; hyaline throughout, veins light brown, microtrichia absent; cells r_1_, r_4_, r_5_, m_3_, + cu*p* closed; C terminates at junction with R_1_; R_4_ terminates in R_1_; R_5_ terminates in R_1_; stump vein (R_3_) at base of R_4_ present, short not reaching R_2_; R_4_ and R_5_ widest apart medially; r-m distinct, R_4__+__5_ and M_1_ apart, connected by crossvein; M_1_ straight at r-m (not curving anteriorly), M_1_ (or M_1_+M_2_) terminates in R_1_; CuA_1_ and CuA_2_ split proximally to m-cu (cell m_3_ narrow proximally); M_3_+CuA_1_ do not terminate together in C; A_1_ undulating, cell a_1_ wide, A_1_ and wing margin further apart proximally than distally; alula well-developed; halter light yellow.

Abdomen: yellow to brown; setation comprised of dense white setae, surface entirely smooth; T1–2 anteriorly yellow otherwise brown, T3 antero-medially brown otherwise yellow, T4–7 yellow to light brown; T1 and anterior ½ of T2 long white setose, remaining T short white setose; T predominantly apubescent; S1–7 yellow; S1–7 short white setose; S predominantly apubescent; T2–4 parallel-sided and not constricted waist-like; bullae on T2 black, transversely elongate, surface entirely smooth, T2 surface anterior to bullae smooth.

♂ terminalia: Fig. 8a, b, c.

**Female:** unknown.

#### Diagnosis

This large species (wing length in males 9.6–12.2 mm, females unknown) is distinguished from congeners by the entirely white pubescent vertex and postgena, by the long postpedicel (about 7 times as long as combined length of scape and pedicel), the long white scutal setation, the yellow postpronotal lobes, the densely grey pubescent mesopostnotum, anatergite, and katatergite, the yellow to light brown coloration of the legs, and the long, erect white setae dorsally on the metathoracic femur.

#### Distribution

Namibia (Karas) and South Africa (Northern Cape) (Fig. 6).

#### Discussion

Hesse 1972 (p. 148) alludes to the morphological similarity of *Namadytes maculiventris* and *Namadytes pallidus*. We regard the differences between the male holotype of *Namadytes maculiventris* and the male holotype and paratype of *Namadytes pallidus* as intraspecific variation. So far, only 4 specimens of this species, all males, have been collected in southern Namibia and Vioolsdrift in South Africa.

#### Type locality

South Africa: Northern Cape: Vioolsdrift (28°46'10''S, 17°37'37''E) (Fig. 6).

#### Biodiversity hotspot

Not known to occur in any of the southern African biodiversity hotspots (Cape Floristic Region, Maputaland-Pondoland-Albany, or Succulent Karoo) (Fig. 6).

### Namadytes vansoni

Hesse, 1969

urn:lsid:zoobank.org:act:CE05440A-6508-4A15-BC27-2A4EA9E52790

Namadytes vansoni Hesse, 1969: 280.
Namadytes prozeskyi Hesse, 1969: 282 **syn. nov.** (ZooBank LSID). Type locality of Namadytes prozeskyi: Namibia: Erongo: Arechadamab (23°10'00''S 015°36'00''E).

#### Materials

- **Type status:** Holotype. **Occurrence:** catalogNumber: TMSA-Dip34; recordedBy: G. van Son; sex: 1 female; lifeStage: Adult; otherCatalogNumbers: AAM-000456; **Taxon:** scientificNameID: urn:lsid:zoobank.org:act:CE05440A-6508-4A15-BC27-2A4EA9E52790; scientificName: *Namadytes vansoni* Hesse, 1969; family: Mydidae; genus: Namadytes; specificEpithet: vansoni; scientificNameAuthorship: Hesse, 1969; **Location:** country: Namibia; stateProvince: Karas; locality: Seeheim; verbatimCoordinates: 26°48'53''S 017°47'57''E; decimalLatitude: -26.81472; decimalLongitude: 17.79917; **Identification:** identifiedBy: A. Hesse; dateIdentified: 1969; **Event:** eventDate: 1933-05-00; **Record Level:** institutionCode: TMSA; collectionCode: Insects; basisOfRecord: PreservedSpecimen
- **Type status:** Holotype. **Occurrence:** catalogNumber: TMSA-Dip35; recordedBy: O. Prozesky; sex: 1 female; lifeStage: Adult; otherCatalogNumbers: AAM-000457; previousIdentifications: Namadytes prozeskyi by A. Hesse in 1969; **Taxon:** scientificNameID: urn:lsid:zoobank.org:act:CE05440A-6508-4A15-BC27-2A4EA9E52790; scientificName: *Namadytes vansoni* Hesse, 1969; family: Mydidae; genus: Namadytes; specificEpithet: vansoni; scientificNameAuthorship: Hesse, 1969; **Location:** country: Namibia; stateProvince: Erongo; locality: Arechadamab, Game Reserve No. 3 (= Namib Naukluft Park); verbatimCoordinates: 23°10'00''S 015°36'00''E; decimalLatitude: -23.16667; decimalLongitude: 15.6; **Identification:** identifiedBy: T. Dikow S. Leon; dateIdentified: 2012; **Event:** eventDate: 1959-10-11; **Record Level:** institutionCode: TMSA; collectionCode: Insects; basisOfRecord: PreservedSpecimen
- **Type status:** Other material. **Occurrence:** catalogNumber: SAM-DIP-A012469; recordedBy: V. Whitehead; sex: 1 male; lifeStage: Adult; otherCatalogNumbers: AAM-003049; **Taxon:** scientificNameID: urn:lsid:zoobank.org:act:CE05440A-6508-4A15-BC27-2A4EA9E52790; scientificName: *Namadytes vansoni* Hesse, 1969; family: Mydidae; genus: Namadytes; specificEpithet: vansoni; scientificNameAuthorship: Hesse, 1969; **Location:** country: Namibia; stateProvince: Kunene; locality: Duineveld No. 529, SW Khorixas; verbatimCoordinates: 20°47'00''S 014°38'00''E; decimalLatitude: -20.78333; decimalLongitude: 14.63333; **Identification:** identifiedBy: T. Dikow S. Leon; dateIdentified: 2012; **Event:** eventDate: 1978-05-14; **Record Level:** institutionCode: SAMC; collectionCode: Insects; basisOfRecord: PreservedSpecimen
- **Type status:** Other material. **Occurrence:** catalogNumber: SAM-DIP-A012479; recordedBy: V. Whitehead; sex: 1 male; lifeStage: Adult; otherCatalogNumbers: AAM-003037; **Taxon:** scientificNameID: urn:lsid:zoobank.org:act:CE05440A-6508-4A15-BC27-2A4EA9E52790; scientificName: *Namadytes vansoni* Hesse, 1969; family: Mydidae; genus: Namadytes; specificEpithet: vansoni; scientificNameAuthorship: Hesse, 1969; **Location:** country: Namibia; stateProvince: Kunene; locality: Rooiberg No. 517, W Khorixas; verbatimCoordinates: 20°27'00''S 014°35'00''E; decimalLatitude: -20.45; decimalLongitude: 14.58333; **Identification:** identifiedBy: T. Dikow S. Leon; dateIdentified: 2012; **Event:** eventDate: 1978-05-12; **Record Level:** institutionCode: SAMC; collectionCode: Insects; basisOfRecord: PreservedSpecimen
- **Type status:** Other material. **Occurrence:** catalogNumber: AAM-000672; recordedBy: H. Brown; sex: 1 female; lifeStage: Adult; otherCatalogNumbers: AAM-000672; previousIdentifications: Namadytes prozeskyi by J. Bowden in; **Taxon:** scientificNameID: urn:lsid:zoobank.org:act:CE05440A-6508-4A15-BC27-2A4EA9E52790; scientificName: *Namadytes vansoni* Hesse, 1969; family: Mydidae; genus: Namadytes; specificEpithet: vansoni; scientificNameAuthorship: Hesse, 1969; **Location:** country: Namibia; stateProvince: Erongo; locality: Kuiseb Namib; verbatimCoordinates: 23°32'33''S 015°01'18''E; decimalLatitude: -23.5425; decimalLongitude: 15.02167; **Identification:** identifiedBy: T. Dikow S. Leon; dateIdentified: 2012; **Event:** eventDate: 1959-05-04; **Record Level:** institutionCode: BMNH; collectionCode: Insects; basisOfRecord: PreservedSpecimen
- **Type status:** Other material. **Occurrence:** catalogNumber: INHS-503368; recordedBy: I. Kapofi M. Irwin; sex: 1 male; lifeStage: Adult; previousIdentifications: Namadytes maculiventris by B. Kondratieff in 2000; **Taxon:** scientificNameID: urn:lsid:zoobank.org:act:CE05440A-6508-4A15-BC27-2A4EA9E52790; scientificName: *Namadytes vansoni* Hesse, 1969; family: Mydidae; genus: Namadytes; specificEpithet: vansoni; scientificNameAuthorship: Hesse, 1969; **Location:** country: Namibia; stateProvince: Erongo; locality: Namib-Naukluft Park, Namib Desert Research Station, Kuiseb River; verbatimElevation: 420 m; verbatimCoordinates: 23°33'45''S 015°02'38''E; decimalLatitude: -23.5625; decimalLongitude: 15.04389; **Identification:** identifiedBy: T. Dikow S. Leon; dateIdentified: 2012; **Event:** samplingProtocol: Malaise trap; eventDate: 1997-03-14–1997-03-26; habitat: riparian vegetation; **Record Level:** institutionCode: INHS; collectionCode: Insects; basisOfRecord: PreservedSpecimen
- **Type status:** Other material. **Occurrence:** catalogNumber: INHS-503373; recordedBy: I. Kapofi M. Irwin; sex: 1 male; lifeStage: Adult; previousIdentifications: Namadytes maculiventris by B. Kondratieff in 2000; **Taxon:** scientificNameID: urn:lsid:zoobank.org:act:CE05440A-6508-4A15-BC27-2A4EA9E52790; scientificName: *Namadytes vansoni* Hesse, 1969; family: Mydidae; genus: Namadytes; specificEpithet: vansoni; scientificNameAuthorship: Hesse, 1969; **Location:** country: Namibia; stateProvince: Erongo; locality: Namib-Naukluft Park, Namib Desert Research Station, Kuiseb River; verbatimElevation: 420 m; verbatimCoordinates: 23°33'45''S 015°02'38''E; decimalLatitude: -23.5625; decimalLongitude: 15.04389; **Identification:** identifiedBy: T. Dikow S. Leon; dateIdentified: 2012; **Event:** samplingProtocol: Malaise trap; eventDate: 1997-03-05–1997-03-14; habitat: riparian vegetation; **Record Level:** institutionCode: INHS; collectionCode: Insects; basisOfRecord: PreservedSpecimen
- **Type status:** Other material. **Occurrence:** catalogNumber: AAM-002824; recordedBy: R. Wharton; sex: 1 female; lifeStage: Adult; otherCatalogNumbers: AAM-002824; previousIdentifications: Namadytes prozeskyi by R. Wharton in 1979; **Taxon:** scientificNameID: urn:lsid:zoobank.org:act:CE05440A-6508-4A15-BC27-2A4EA9E52790; scientificName: *Namadytes vansoni* Hesse, 1969; family: Mydidae; genus: Namadytes; specificEpithet: vansoni; scientificNameAuthorship: Hesse, 1969; **Location:** country: Namibia; stateProvince: Erongo; locality: Gobabeb, Kuiseb River; verbatimCoordinates: 23°33'37''S 015°02'26''E; decimalLatitude: -23.56028; decimalLongitude: 15.04056; **Identification:** identifiedBy: T. Dikow S. Leon; dateIdentified: 2012; **Event:** eventDate: 1979-06-10; **Record Level:** institutionCode: NMSA; collectionCode: Insects; basisOfRecord: PreservedSpecimen
- **Type status:** Other material. **Occurrence:** catalogNumber: AAM-002825; recordedBy: R. Wharton; sex: 1 male; lifeStage: Adult; otherCatalogNumbers: AAM-002825; previousIdentifications: Namadytes prozeskyi by R. Wharton in 1979; **Taxon:** scientificNameID: urn:lsid:zoobank.org:act:CE05440A-6508-4A15-BC27-2A4EA9E52790; scientificName: *Namadytes vansoni* Hesse, 1969; family: Mydidae; genus: Namadytes; specificEpithet: vansoni; scientificNameAuthorship: Hesse, 1969; **Location:** country: Namibia; stateProvince: Erongo; locality: Gobabeb, Kuiseb River; verbatimCoordinates: 23°33'37''S 015°02'26''E; decimalLatitude: -23.56028; decimalLongitude: 15.04056; **Identification:** identifiedBy: T. Dikow S. Leon; dateIdentified: 2012; **Event:** eventDate: 1979-06-12; **Record Level:** institutionCode: NMSA; collectionCode: Insects; basisOfRecord: PreservedSpecimen
- **Type status:** Other material. **Occurrence:** catalogNumber: AAM-002827; recordedBy: R. Wharton; sex: 1 female; lifeStage: Adult; otherCatalogNumbers: AAM-002827; previousIdentifications: Namadytes prozeskyi by R. Wharton in 1979; **Taxon:** scientificNameID: urn:lsid:zoobank.org:act:CE05440A-6508-4A15-BC27-2A4EA9E52790; scientificName: *Namadytes vansoni* Hesse, 1969; family: Mydidae; genus: Namadytes; specificEpithet: vansoni; scientificNameAuthorship: Hesse, 1969; **Location:** country: Namibia; stateProvince: Erongo; locality: Gobabeb, Kuiseb River; verbatimCoordinates: 23°33'37''S 015°02'26''E; decimalLatitude: -23.56028; decimalLongitude: 15.04056; **Identification:** identifiedBy: T. Dikow S. Leon; dateIdentified: 2012; **Event:** eventDate: 1979-06-09; **Record Level:** institutionCode: NMSA; collectionCode: Insects; basisOfRecord: PreservedSpecimen
- **Type status:** Other material. **Occurrence:** catalogNumber: AAM-002828; recordedBy: R. Wharton; sex: 1 male; lifeStage: Adult; otherCatalogNumbers: AAM-002828; previousIdentifications: Namadytes prozeskyi by R. Wharton in 1979; **Taxon:** scientificNameID: urn:lsid:zoobank.org:act:CE05440A-6508-4A15-BC27-2A4EA9E52790; scientificName: *Namadytes vansoni* Hesse, 1969; family: Mydidae; genus: Namadytes; specificEpithet: vansoni; scientificNameAuthorship: Hesse, 1969; **Location:** country: Namibia; stateProvince: Erongo; locality: Gobabeb; verbatimCoordinates: 23°33'37''S 015°02'26''E; decimalLatitude: -23.56028; decimalLongitude: 15.04056; **Identification:** identifiedBy: T. Dikow S. Leon; dateIdentified: 2012; **Event:** eventDate: 1979-05-11; **Record Level:** institutionCode: NMSA; collectionCode: Insects; basisOfRecord: PreservedSpecimen
- **Type status:** Other material. **Occurrence:** catalogNumber: INHS-503356; recordedBy: I. Kapofi M. Irwin; sex: 1 male; lifeStage: Adult; **Taxon:** scientificNameID: urn:lsid:zoobank.org:act:CE05440A-6508-4A15-BC27-2A4EA9E52790; scientificName: *Namadytes vansoni* Hesse, 1969; family: Mydidae; genus: Namadytes; specificEpithet: vansoni; scientificNameAuthorship: Hesse, 1969; **Location:** country: Namibia; stateProvince: Erongo; locality: Namib-Naukluft Park, Namib Desert Research Station, Kuiseb River; verbatimElevation: 420 m; verbatimCoordinates: 23°33'45''S 015°02'38''E; decimalLatitude: -23.5625; decimalLongitude: 15.04389; **Identification:** identifiedBy: T. Dikow S. Leon; dateIdentified: 2012; **Event:** samplingProtocol: Malaise trap; eventDate: 1997-03-14–1997-03-26; habitat: riparian vegetation; **Record Level:** institutionCode: INHS; collectionCode: Insects; basisOfRecord: PreservedSpecimen
- **Type status:** Other material. **Occurrence:** catalogNumber: INHS-503365; recordedBy: I. Kapofi M. Irwin; sex: 1 male; lifeStage: Adult; **Taxon:** scientificNameID: urn:lsid:zoobank.org:act:CE05440A-6508-4A15-BC27-2A4EA9E52790; scientificName: *Namadytes vansoni* Hesse, 1969; family: Mydidae; genus: Namadytes; specificEpithet: vansoni; scientificNameAuthorship: Hesse, 1969; **Location:** country: Namibia; stateProvince: Erongo; locality: Namib-Naukluft Park, Namib Desert Research Station, Kuiseb River; verbatimElevation: 420 m; verbatimCoordinates: 23°33'45''S 015°02'38''E; decimalLatitude: -23.5625; decimalLongitude: 15.04389; **Identification:** identifiedBy: T. Dikow S. Leon; dateIdentified: 2012; **Event:** samplingProtocol: Malaise trap; eventDate: 1997-03-26–1997-04-02; habitat: riparian vegetation; **Record Level:** institutionCode: INHS; collectionCode: Insects; basisOfRecord: PreservedSpecimen
- **Type status:** Other material. **Occurrence:** catalogNumber: INHS-503360; recordedBy: I. Kapofi M. Irwin; sex: 1 female; lifeStage: Adult; **Taxon:** scientificNameID: urn:lsid:zoobank.org:act:CE05440A-6508-4A15-BC27-2A4EA9E52790; scientificName: *Namadytes vansoni* Hesse, 1969; family: Mydidae; genus: Namadytes; specificEpithet: vansoni; scientificNameAuthorship: Hesse, 1969; **Location:** country: Namibia; stateProvince: Erongo; locality: Namib-Naukluft Park, Namib Desert Research Station, Kuiseb River; verbatimElevation: 420 m; verbatimCoordinates: 23°33'45''S 015°02'38''E; decimalLatitude: -23.5625; decimalLongitude: 15.04389; **Identification:** identifiedBy: T. Dikow S. Leon; dateIdentified: 2012; **Event:** samplingProtocol: Malaise trap; eventDate: 1997-04-21–1997-04-28; habitat: riparian vegetation; **Record Level:** institutionCode: INHS; collectionCode: Insects; basisOfRecord: PreservedSpecimen
- **Type status:** Other material. **Occurrence:** catalogNumber: INHS-503359; recordedBy: I. Kapofi M. Irwin; sex: 1 male; lifeStage: Adult; **Taxon:** scientificNameID: urn:lsid:zoobank.org:act:CE05440A-6508-4A15-BC27-2A4EA9E52790; scientificName: *Namadytes vansoni* Hesse, 1969; family: Mydidae; genus: Namadytes; specificEpithet: vansoni; scientificNameAuthorship: Hesse, 1969; **Location:** country: Namibia; stateProvince: Erongo; locality: Namib-Naukluft Park, Namib Desert Research Station, Kuiseb River; verbatimElevation: 420 m; verbatimCoordinates: 23°33'45''S 015°02'38''E; decimalLatitude: -23.5625; decimalLongitude: 15.04389; **Identification:** identifiedBy: T. Dikow S. Leon; dateIdentified: 2012; **Event:** samplingProtocol: Malaise trap; eventDate: 1997-04-09–1997-04-21; habitat: riparian vegetation; **Record Level:** institutionCode: INHS; collectionCode: Insects; basisOfRecord: PreservedSpecimen
- **Type status:** Other material. **Occurrence:** catalogNumber: INHS-503364; recordedBy: I. Kapofi M. Irwin; sex: 1 male; lifeStage: Adult; **Taxon:** scientificNameID: urn:lsid:zoobank.org:act:CE05440A-6508-4A15-BC27-2A4EA9E52790; scientificName: *Namadytes vansoni* Hesse, 1969; family: Mydidae; genus: Namadytes; specificEpithet: vansoni; scientificNameAuthorship: Hesse, 1969; **Location:** country: Namibia; stateProvince: Erongo; locality: Namib-Naukluft Park, Namib Desert Research Station, Kuiseb River; verbatimElevation: 420 m; verbatimCoordinates: 23°33'45''S 015°02'38''E; decimalLatitude: -23.5625; decimalLongitude: 15.04389; **Identification:** identifiedBy: T. Dikow S. Leon; dateIdentified: 2012; **Event:** samplingProtocol: Malaise trap; eventDate: 1997-02-26–1997-03-05; habitat: riparian vegetation; **Record Level:** institutionCode: INHS; collectionCode: Insects; basisOfRecord: PreservedSpecimen
- **Type status:** Other material. **Occurrence:** catalogNumber: AAM-002985; recordedBy: J. Potgieter; sex: 1 female; lifeStage: Adult; otherCatalogNumbers: AAM-002985; **Taxon:** scientificNameID: urn:lsid:zoobank.org:act:CE05440A-6508-4A15-BC27-2A4EA9E52790; scientificName: *Namadytes vansoni* Hesse, 1969; family: Mydidae; genus: Namadytes; specificEpithet: vansoni; scientificNameAuthorship: Hesse, 1969; **Location:** country: Namibia; stateProvince: Erongo; locality: Ganab, Game Reserve No. 3 (= Namib Naukluft Park); verbatimCoordinates: 23°06'10''S 015°31'45''E; decimalLatitude: -23.10278; decimalLongitude: 15.52917; **Identification:** identifiedBy: T. Dikow S. Leon; dateIdentified: 2012; **Event:** eventDate: 1967-04-21; **Record Level:** institutionCode: NMSA; collectionCode: Insects; basisOfRecord: PreservedSpecimen
- **Type status:** Other material. **Occurrence:** catalogNumber: AAM-002986; recordedBy: J. Londt B. Stuckenberg; sex: 1 male; lifeStage: Adult; otherCatalogNumbers: AAM-002986; **Taxon:** scientificNameID: urn:lsid:zoobank.org:act:CE05440A-6508-4A15-BC27-2A4EA9E52790; scientificName: *Namadytes vansoni* Hesse, 1969; family: Mydidae; genus: Namadytes; specificEpithet: vansoni; scientificNameAuthorship: Hesse, 1969; **Location:** country: Namibia; stateProvince: Erongo; locality: Windhoek, 158 km W; verbatimCoordinates: 22°44'21''S 015°55'57''E; decimalLatitude: -22.73917; decimalLongitude: 15.9325; **Identification:** identifiedBy: T. Dikow S. Leon; dateIdentified: 2012; **Event:** eventDate: 1983-04-22; habitat: thornveld in dry river bed; **Record Level:** institutionCode: NMSA; collectionCode: Insects; basisOfRecord: PreservedSpecimen
- **Type status:** Other material. **Occurrence:** catalogNumber: AAM-002989; recordedBy: B. Stuckenberg J. Londt; sex: 1 female; lifeStage: Adult; otherCatalogNumbers: AAM-002989; **Taxon:** scientificNameID: urn:lsid:zoobank.org:act:CE05440A-6508-4A15-BC27-2A4EA9E52790; scientificName: *Namadytes vansoni* Hesse, 1969; family: Mydidae; genus: Namadytes; specificEpithet: vansoni; scientificNameAuthorship: Hesse, 1969; **Location:** country: Namibia; stateProvince: Erongo; locality: Swakopmund, 110 km E; verbatimCoordinates: 22°55'01''S 015°28'12''E; decimalLatitude: -22.91694; decimalLongitude: 15.47; **Identification:** identifiedBy: T. Dikow S. Leon; dateIdentified: 2012; **Event:** eventDate: 1983-04-22; habitat: barren gravel plain; **Record Level:** institutionCode: NMSA; collectionCode: Insects; basisOfRecord: PreservedSpecimen
- **Type status:** Other material. **Occurrence:** catalogNumber: AAM-002997; recordedBy: I. Kapofi M. Irwin; sex: 1 male; lifeStage: Adult; otherCatalogNumbers: AAM-002997; **Taxon:** scientificNameID: urn:lsid:zoobank.org:act:CE05440A-6508-4A15-BC27-2A4EA9E52790; scientificName: *Namadytes vansoni* Hesse, 1969; family: Mydidae; genus: Namadytes; specificEpithet: vansoni; scientificNameAuthorship: Hesse, 1969; **Location:** country: Namibia; stateProvince: Erongo; locality: Namib-Naukluft Park, Namib Desert Research Station, Kuiseb River; verbatimElevation: 420 m; verbatimCoordinates: 23°33'45''S 015°02'38''E; decimalLatitude: -23.5625; decimalLongitude: 15.04389; **Identification:** identifiedBy: T. Dikow S. Leon; dateIdentified: 2012; **Event:** samplingProtocol: Malaise trap; eventDate: 1997-03-05–1997-03-14; habitat: riparian vegetation; **Record Level:** institutionCode: CSCA; collectionCode: Insects; basisOfRecord: PreservedSpecimen
- **Type status:** Other material. **Occurrence:** catalogNumber: AAM-002999; recordedBy: I. Kapofi M. Irwin; sex: 1 female; lifeStage: Adult; otherCatalogNumbers: AAM-002999; **Taxon:** scientificNameID: urn:lsid:zoobank.org:act:CE05440A-6508-4A15-BC27-2A4EA9E52790; scientificName: *Namadytes vansoni* Hesse, 1969; family: Mydidae; genus: Namadytes; specificEpithet: vansoni; scientificNameAuthorship: Hesse, 1969; **Location:** country: Namibia; stateProvince: Erongo; locality: Namib-Naukluft Park, Namib Desert Research Station, Kuiseb River; verbatimElevation: 420 m; verbatimCoordinates: 23°33'45''S 015°02'38''E; decimalLatitude: -23.5625; decimalLongitude: 15.04389; **Identification:** identifiedBy: T. Dikow S. Leon; dateIdentified: 2012; **Event:** samplingProtocol: Malaise trap; eventDate: 1997-04-21–1997-04-28; habitat: riparian vegetation; **Record Level:** institutionCode: CSCA; collectionCode: Insects; basisOfRecord: PreservedSpecimen
- **Type status:** Other material. **Occurrence:** catalogNumber: NMNW-H12725; sex: 1 female; lifeStage: Adult; otherCatalogNumbers: AAM-003007; **Taxon:** scientificNameID: urn:lsid:zoobank.org:act:CE05440A-6508-4A15-BC27-2A4EA9E52790; scientificName: *Namadytes vansoni* Hesse, 1969; family: Mydidae; genus: Namadytes; specificEpithet: vansoni; scientificNameAuthorship: Hesse, 1969; **Location:** country: Namibia; stateProvince: Kunene; locality: Outjo, Bethanis No. 514; verbatimCoordinates: 20°24'00''S 014°24'00''E; decimalLatitude: -20.4; decimalLongitude: 14.4; **Identification:** identifiedBy: T. Dikow S. Leon; dateIdentified: 2012; **Event:** eventDate: 1973-05-08–1973-05-10; **Record Level:** institutionCode: NMNW; collectionCode: Insects; basisOfRecord: PreservedSpecimen
- **Type status:** Other material. **Occurrence:** catalogNumber: NMNW-H8279; sex: 1 female; lifeStage: Adult; otherCatalogNumbers: AAM-003009; **Taxon:** scientificNameID: urn:lsid:zoobank.org:act:CE05440A-6508-4A15-BC27-2A4EA9E52790; scientificName: *Namadytes vansoni* Hesse, 1969; family: Mydidae; genus: Namadytes; specificEpithet: vansoni; scientificNameAuthorship: Hesse, 1969; **Location:** country: Namibia; stateProvince: Karas; county: Keetmanshoop; locality: Rotegab No. 95; verbatimCoordinates: 27°20'00''S 018°25'00''E; decimalLatitude: -27.33333; decimalLongitude: 18.41667; **Identification:** identifiedBy: T. Dikow S. Leon; dateIdentified: 2012; **Event:** eventDate: 1972-04-27; **Record Level:** institutionCode: NMNW; collectionCode: Insects; basisOfRecord: PreservedSpecimen
- **Type status:** Other material. **Occurrence:** catalogNumber: NMNW-H18282; sex: 1 male; lifeStage: Adult; otherCatalogNumbers: AAM-003011; **Taxon:** scientificNameID: urn:lsid:zoobank.org:act:CE05440A-6508-4A15-BC27-2A4EA9E52790; scientificName: *Namadytes vansoni* Hesse, 1969; family: Mydidae; genus: Namadytes; specificEpithet: vansoni; scientificNameAuthorship: Hesse, 1969; **Location:** country: Namibia; stateProvince: Karas; county: Namaland; locality: Mukorob No. 14; verbatimCoordinates: 25°29'00''S 018°10'00''E; decimalLatitude: -25.48333; decimalLongitude: 18.16667; **Identification:** identifiedBy: T. Dikow S. Leon; dateIdentified: 2012; **Event:** eventDate: 1974-04-12–1974-04-14; **Record Level:** institutionCode: NMNW; collectionCode: Insects; basisOfRecord: PreservedSpecimen
- **Type status:** Other material. **Occurrence:** catalogNumber: NMNW-H36196; recordedBy: M.-L. Penrith S. Louw; sex: 1 female; lifeStage: Adult; otherCatalogNumbers: AAM-003015; **Taxon:** scientificNameID: urn:lsid:zoobank.org:act:CE05440A-6508-4A15-BC27-2A4EA9E52790; scientificName: *Namadytes vansoni* Hesse, 1969; family: Mydidae; genus: Namadytes; specificEpithet: vansoni; scientificNameAuthorship: Hesse, 1969; **Location:** country: Namibia; stateProvince: Kunene; locality: Damaraland, Duineveld No. 529; verbatimCoordinates: 20°47'00''S 014°38'00''E; decimalLatitude: -20.78333; decimalLongitude: 14.63333; **Identification:** identifiedBy: T. Dikow S. Leon; dateIdentified: 2012; **Event:** eventDate: 1978-05-14–1978-05-16; **Record Level:** institutionCode: NMNW; collectionCode: Insects; basisOfRecord: PreservedSpecimen
- **Type status:** Other material. **Occurrence:** catalogNumber: AAM-003019; recordedBy: F. and S. Gess; sex: 1 male; lifeStage: Adult; otherCatalogNumbers: AAM-003019; **Taxon:** scientificNameID: urn:lsid:zoobank.org:act:CE05440A-6508-4A15-BC27-2A4EA9E52790; scientificName: *Namadytes vansoni* Hesse, 1969; family: Mydidae; genus: Namadytes; specificEpithet: vansoni; scientificNameAuthorship: Hesse, 1969; **Location:** country: Namibia; stateProvince: Karas; locality: Gibeon, 41 km SW on 1089; verbatimCoordinates: 25°20'00''S 017°29'00''E; decimalLatitude: -25.33333; decimalLongitude: 17.48333; **Identification:** identifiedBy: T. Dikow S. Leon; dateIdentified: 2012; **Event:** eventDate: 1999-03-24; **Record Level:** institutionCode: AMGS; collectionCode: Insects; basisOfRecord: PreservedSpecimen
- **Type status:** Other material. **Occurrence:** catalogNumber: AAM-003020; recordedBy: F. and S. Gess; sex: 1 male; lifeStage: Adult; otherCatalogNumbers: AAM-003020; **Taxon:** scientificNameID: urn:lsid:zoobank.org:act:CE05440A-6508-4A15-BC27-2A4EA9E52790; scientificName: *Namadytes vansoni* Hesse, 1969; family: Mydidae; genus: Namadytes; specificEpithet: vansoni; scientificNameAuthorship: Hesse, 1969; **Location:** country: Namibia; stateProvince: Erongo; locality: Usakos, Phillips Caves; verbatimCoordinates: 21°52'16''S 015°35'18''E; decimalLatitude: -21.87111; decimalLongitude: 15.58833; **Identification:** identifiedBy: T. Dikow S. Leon; dateIdentified: 2012; **Event:** eventDate: 2002-04-23; **Record Level:** institutionCode: AMGS; collectionCode: Insects; basisOfRecord: PreservedSpecimen
- **Type status:** Other material. **Occurrence:** catalogNumber: AAM-003058; recordedBy: National Collection Kuiseb Survey; sex: 1 male; lifeStage: Adult; otherCatalogNumbers: AAM-003058; **Taxon:** scientificNameID: urn:lsid:zoobank.org:act:CE05440A-6508-4A15-BC27-2A4EA9E52790; scientificName: *Namadytes vansoni* Hesse, 1969; family: Mydidae; genus: Namadytes; specificEpithet: vansoni; scientificNameAuthorship: Hesse, 1969; **Location:** country: Namibia; stateProvince: Erongo; locality: Namib-Naukluft Park, Kuiseb River near Gobabeb; verbatimCoordinates: 23°34'00''S 015°03'00''E; decimalLatitude: -23.56667; decimalLongitude: 15.05; **Identification:** identifiedBy: T. Dikow S. Leon; dateIdentified: 2012; **Event:** samplingProtocol: Malaise trap; eventDate: 1983-02-18–1983-03-20; **Record Level:** institutionCode: SANC; collectionCode: Insects; basisOfRecord: PreservedSpecimen
- **Type status:** Other material. **Occurrence:** catalogNumber: AAM-003059; recordedBy: C. Eardley; sex: 1 male; lifeStage: Adult; otherCatalogNumbers: AAM-003059; **Taxon:** scientificNameID: urn:lsid:zoobank.org:act:CE05440A-6508-4A15-BC27-2A4EA9E52790; scientificName: *Namadytes vansoni* Hesse, 1969; family: Mydidae; genus: Namadytes; specificEpithet: vansoni; scientificNameAuthorship: Hesse, 1969; **Location:** country: Namibia; stateProvince: Erongo; locality: Mariental, 52 km W; verbatimCoordinates: 24°46'35''S 017°31'13''E; decimalLatitude: -24.77639; decimalLongitude: 17.52028; **Identification:** identifiedBy: T. Dikow S. Leon; dateIdentified: 2012; **Event:** eventDate: 1983-03-27; **Record Level:** institutionCode: SANC; collectionCode: Insects; basisOfRecord: PreservedSpecimen
- **Type status:** Other material. **Occurrence:** catalogNumber: SAM-DIP-A007149; recordedBy: H. Brown; sex: 1 female; lifeStage: Adult; otherCatalogNumbers: AAM-002909; previousIdentifications: Namadytes prozeskyi by A. Hesse in 1969; **Taxon:** scientificNameID: urn:lsid:zoobank.org:act:CE05440A-6508-4A15-BC27-2A4EA9E52790; scientificName: *Namadytes vansoni* Hesse, 1969; family: Mydidae; genus: Namadytes; specificEpithet: vansoni; scientificNameAuthorship: Hesse, 1969; **Location:** country: Namibia; stateProvince: Erongo; locality: Hope Mine, 48 km N, Kuiseb River; verbatimCoordinates: 23°33'56''S 015°16'16''E; decimalLatitude: -23.56556; decimalLongitude: 15.27111; **Identification:** identifiedBy: T. Dikow S. Leon; dateIdentified: 2012; **Event:** eventDate: 1959-05-11; **Record Level:** institutionCode: SAMC; collectionCode: Insects; basisOfRecord: PreservedSpecimen
- **Type status:** Other material. **Occurrence:** catalogNumber: NMNW-H12726; sex: 1 female; lifeStage: Adult; otherCatalogNumbers: AAM-003008; **Taxon:** scientificNameID: urn:lsid:zoobank.org:act:CE05440A-6508-4A15-BC27-2A4EA9E52790; scientificName: *Namadytes vansoni* Hesse, 1969; family: Mydidae; genus: Namadytes; specificEpithet: vansoni; scientificNameAuthorship: Hesse, 1969; **Location:** country: Namibia; stateProvince: Kunene; locality: Outjo, Bethanis No. 514; verbatimCoordinates: 20°24'00''S 014°24'00''E; decimalLatitude: -20.4; decimalLongitude: 14.4; **Identification:** identifiedBy: T. Dikow S. Leon; dateIdentified: 2012; **Event:** eventDate: 1973-05-08–1973-05-10; **Record Level:** institutionCode: NMNW; collectionCode: Insects; basisOfRecord: PreservedSpecimen
- **Type status:** Other material. **Occurrence:** catalogNumber: NMNW-H12727; sex: 1 male; lifeStage: Adult; otherCatalogNumbers: AAM-003006; **Taxon:** scientificNameID: urn:lsid:zoobank.org:act:CE05440A-6508-4A15-BC27-2A4EA9E52790; scientificName: *Namadytes vansoni* Hesse, 1969; family: Mydidae; genus: Namadytes; specificEpithet: vansoni; scientificNameAuthorship: Hesse, 1969; **Location:** country: Namibia; stateProvince: Kunene; locality: Outjo, Bethanis No. 514; verbatimCoordinates: 20°24'00''S 014°24'00''E; decimalLatitude: -20.4; decimalLongitude: 14.4; **Identification:** identifiedBy: T. Dikow S. Leon; dateIdentified: 2012; **Event:** eventDate: 1973-05-08–1973-05-10; **Record Level:** institutionCode: NMNW; collectionCode: Insects; basisOfRecord: PreservedSpecimen
- **Type status:** Other material. **Occurrence:** catalogNumber: INHS-503370; recordedBy: I. Kapofi M. Irwin; sex: 1 male; lifeStage: Adult; previousIdentifications: Namadytes maculiventris by B. Kondratieff in 2000; **Taxon:** scientificNameID: urn:lsid:zoobank.org:act:CE05440A-6508-4A15-BC27-2A4EA9E52790; scientificName: *Namadytes vansoni* Hesse, 1969; family: Mydidae; genus: Namadytes; specificEpithet: vansoni; scientificNameAuthorship: Hesse, 1969; **Location:** country: Namibia; stateProvince: Erongo; locality: Namib-Naukluft Park, Namib Desert Research Station, Kuiseb River; verbatimElevation: 420 m; verbatimCoordinates: 23°33'45''S 015°02'38''E; decimalLatitude: -23.5625; decimalLongitude: 15.04389; **Identification:** identifiedBy: T. Dikow S. Leon; dateIdentified: 2012; **Event:** samplingProtocol: Malaise trap; eventDate: 1997-03-14–1997-03-26; habitat: riparian vegetation; **Record Level:** institutionCode: INHS; collectionCode: Insects; basisOfRecord: PreservedSpecimen
- **Type status:** Other material. **Occurrence:** catalogNumber: INHS-503371; recordedBy: I. Kapofi M. Irwin; sex: 1 male; lifeStage: Adult; previousIdentifications: Namadytes maculiventris by B. Kondratieff in 2000; **Taxon:** scientificNameID: urn:lsid:zoobank.org:act:CE05440A-6508-4A15-BC27-2A4EA9E52790; scientificName: *Namadytes vansoni* Hesse, 1969; family: Mydidae; genus: Namadytes; specificEpithet: vansoni; scientificNameAuthorship: Hesse, 1969; **Location:** country: Namibia; stateProvince: Erongo; locality: Namib-Naukluft Park, Namib Desert Research Station, Kuiseb River; verbatimElevation: 420 m; verbatimCoordinates: 23°33'45''S 015°02'38''E; decimalLatitude: -23.5625; decimalLongitude: 15.04389; **Identification:** identifiedBy: T. Dikow S. Leon; dateIdentified: 2012; **Event:** samplingProtocol: Malaise trap; eventDate: 1997-03-14–1997-03-26; habitat: riparian vegetation; **Record Level:** institutionCode: INHS; collectionCode: Insects; basisOfRecord: PreservedSpecimen
- **Type status:** Other material. **Occurrence:** catalogNumber: INHS-503369; recordedBy: I. Kapofi M. Irwin; sex: 1 male; lifeStage: Adult; previousIdentifications: Namadytes maculiventris by B. Kondratieff in 2000; **Taxon:** scientificNameID: urn:lsid:zoobank.org:act:CE05440A-6508-4A15-BC27-2A4EA9E52790; scientificName: *Namadytes vansoni* Hesse, 1969; family: Mydidae; genus: Namadytes; specificEpithet: vansoni; scientificNameAuthorship: Hesse, 1969; **Location:** country: Namibia; stateProvince: Erongo; locality: Namib-Naukluft Park, Namib Desert Research Station, Kuiseb River; verbatimElevation: 420 m; verbatimCoordinates: 23°33'45''S 015°02'38''E; decimalLatitude: -23.5625; decimalLongitude: 15.04389; **Identification:** identifiedBy: T. Dikow S. Leon; dateIdentified: 2012; **Event:** samplingProtocol: Malaise trap; eventDate: 1997-03-14–1997-03-26; habitat: riparian vegetation; **Record Level:** institutionCode: INHS; collectionCode: Insects; basisOfRecord: PreservedSpecimen
- **Type status:** Other material. **Occurrence:** catalogNumber: INHS-503372; recordedBy: I. Kapofi M. Irwin; sex: 1 male; lifeStage: Adult; previousIdentifications: Namadytes maculiventris by B. Kondratieff in 2000; **Taxon:** scientificNameID: urn:lsid:zoobank.org:act:CE05440A-6508-4A15-BC27-2A4EA9E52790; scientificName: *Namadytes vansoni* Hesse, 1969; family: Mydidae; genus: Namadytes; specificEpithet: vansoni; scientificNameAuthorship: Hesse, 1969; **Location:** country: Namibia; stateProvince: Erongo; locality: Namib-Naukluft Park, Namib Desert Research Station, Kuiseb River; verbatimElevation: 420 m; verbatimCoordinates: 23°33'45''S 015°02'38''E; decimalLatitude: -23.5625; decimalLongitude: 15.04389; **Identification:** identifiedBy: T. Dikow S. Leon; dateIdentified: 2012; **Event:** samplingProtocol: Malaise trap; eventDate: 1997-03-05–1997-03-14; habitat: riparian vegetation; **Record Level:** institutionCode: INHS; collectionCode: Insects; basisOfRecord: PreservedSpecimen
- **Type status:** Other material. **Occurrence:** catalogNumber: AAM-002998; recordedBy: I. Kapofi M. Irwin; sex: 1 male; lifeStage: Adult; otherCatalogNumbers: AAM-002998; **Taxon:** scientificNameID: urn:lsid:zoobank.org:act:CE05440A-6508-4A15-BC27-2A4EA9E52790; scientificName: *Namadytes vansoni* Hesse, 1969; family: Mydidae; genus: Namadytes; specificEpithet: vansoni; scientificNameAuthorship: Hesse, 1969; **Location:** country: Namibia; stateProvince: Erongo; locality: Namib-Naukluft Park, Namib Desert Research Station, Kuiseb River; verbatimElevation: 420 m; verbatimCoordinates: 23°33'45''S 015°02'38''E; decimalLatitude: -23.5625; decimalLongitude: 15.04389; **Identification:** identifiedBy: T. Dikow S. Leon; dateIdentified: 2012; **Event:** samplingProtocol: Malaise trap; eventDate: 1997-03-05–1997-03-14; habitat: riparian vegetation; **Record Level:** institutionCode: CSCA; collectionCode: Insects; basisOfRecord: PreservedSpecimen
- **Type status:** Other material. **Occurrence:** catalogNumber: SAM-DIP-A012469; recordedBy: V. Whitehead; sex: 1 male; lifeStage: Adult; otherCatalogNumbers: AAM-003043; **Taxon:** scientificNameID: urn:lsid:zoobank.org:act:CE05440A-6508-4A15-BC27-2A4EA9E52790; scientificName: *Namadytes vansoni* Hesse, 1969; family: Mydidae; genus: Namadytes; specificEpithet: vansoni; scientificNameAuthorship: Hesse, 1969; **Location:** country: Namibia; stateProvince: Kunene; locality: Duineveld No. 529, SW Khorixas; verbatimCoordinates: 20°47'00''S 014°38'00''E; decimalLatitude: -20.78333; decimalLongitude: 14.63333; **Identification:** identifiedBy: T. Dikow S. Leon; dateIdentified: 2012; **Event:** eventDate: 1978-05-14; **Record Level:** institutionCode: SAMC; collectionCode: Insects; basisOfRecord: PreservedSpecimen
- **Type status:** Other material. **Occurrence:** catalogNumber: SAM-DIP-A012467; recordedBy: V. Whitehead; sex: 1 male; lifeStage: Adult; otherCatalogNumbers: AAM-003046; **Taxon:** scientificNameID: urn:lsid:zoobank.org:act:CE05440A-6508-4A15-BC27-2A4EA9E52790; scientificName: *Namadytes vansoni* Hesse, 1969; family: Mydidae; genus: Namadytes; specificEpithet: vansoni; scientificNameAuthorship: Hesse, 1969; **Location:** country: Namibia; stateProvince: Kunene; locality: Duineveld No. 529, SW Khorixas; verbatimCoordinates: 20°47'00''S 014°38'00''E; decimalLatitude: -20.78333; decimalLongitude: 14.63333; **Identification:** identifiedBy: T. Dikow S. Leon; dateIdentified: 2012; **Event:** eventDate: 1978-05-14; **Record Level:** institutionCode: SAMC; collectionCode: Insects; basisOfRecord: PreservedSpecimen
- **Type status:** Other material. **Occurrence:** catalogNumber: SAM-DIP-A012469; recordedBy: V. Whitehead; sex: 1 male; lifeStage: Adult; otherCatalogNumbers: AAM-003048; **Taxon:** scientificNameID: urn:lsid:zoobank.org:act:CE05440A-6508-4A15-BC27-2A4EA9E52790; scientificName: *Namadytes vansoni* Hesse, 1969; family: Mydidae; genus: Namadytes; specificEpithet: vansoni; scientificNameAuthorship: Hesse, 1969; **Location:** country: Namibia; stateProvince: Kunene; locality: Duineveld No. 529, SW Khorixas; verbatimCoordinates: 20°47'00''S 014°38'00''E; decimalLatitude: -20.78333; decimalLongitude: 14.63333; **Identification:** identifiedBy: T. Dikow S. Leon; dateIdentified: 2012; **Event:** eventDate: 1978-05-14; **Record Level:** institutionCode: SAMC; collectionCode: Insects; basisOfRecord: PreservedSpecimen
- **Type status:** Other material. **Occurrence:** catalogNumber: SAM-DIP-A012469; recordedBy: V. Whitehead; sex: 1 male; lifeStage: Adult; otherCatalogNumbers: AAM-003047; **Taxon:** scientificNameID: urn:lsid:zoobank.org:act:CE05440A-6508-4A15-BC27-2A4EA9E52790; scientificName: *Namadytes vansoni* Hesse, 1969; family: Mydidae; genus: Namadytes; specificEpithet: vansoni; scientificNameAuthorship: Hesse, 1969; **Location:** country: Namibia; stateProvince: Kunene; locality: Duineveld No. 529, SW Khorixas; verbatimCoordinates: 20°47'00''S 014°38'00''E; decimalLatitude: -20.78333; decimalLongitude: 14.63333; **Identification:** identifiedBy: T. Dikow S. Leon; dateIdentified: 2012; **Event:** eventDate: 1978-05-14; **Record Level:** institutionCode: SAMC; collectionCode: Insects; basisOfRecord: PreservedSpecimen
- **Type status:** Other material. **Occurrence:** catalogNumber: SAM-DIP-A012470; recordedBy: V. Whitehead; sex: 1 female; lifeStage: Adult; otherCatalogNumbers: AAM-003050; **Taxon:** scientificNameID: urn:lsid:zoobank.org:act:CE05440A-6508-4A15-BC27-2A4EA9E52790; scientificName: *Namadytes vansoni* Hesse, 1969; family: Mydidae; genus: Namadytes; specificEpithet: vansoni; scientificNameAuthorship: Hesse, 1969; **Location:** country: Namibia; stateProvince: Kunene; locality: Duineveld No. 529, SW Khorixas; verbatimCoordinates: 20°47'00''S 014°38'00''E; decimalLatitude: -20.78333; decimalLongitude: 14.63333; **Identification:** identifiedBy: T. Dikow S. Leon; dateIdentified: 2012; **Event:** eventDate: 1978-05-14; **Record Level:** institutionCode: SAMC; collectionCode: Insects; basisOfRecord: PreservedSpecimen
- **Type status:** Other material. **Occurrence:** catalogNumber: SAM-DIP-A012468; recordedBy: V. Whitehead; sex: 1 female; lifeStage: Adult; otherCatalogNumbers: AAM-003041; **Taxon:** scientificNameID: urn:lsid:zoobank.org:act:CE05440A-6508-4A15-BC27-2A4EA9E52790; scientificName: *Namadytes vansoni* Hesse, 1969; family: Mydidae; genus: Namadytes; specificEpithet: vansoni; scientificNameAuthorship: Hesse, 1969; **Location:** country: Namibia; stateProvince: Kunene; locality: Duineveld No. 529, SW Khorixas; verbatimCoordinates: 20°47'00''S 014°38'00''E; decimalLatitude: -20.78333; decimalLongitude: 14.63333; **Identification:** identifiedBy: T. Dikow S. Leon; dateIdentified: 2012; **Event:** eventDate: 1978-05-14; **Record Level:** institutionCode: SAMC; collectionCode: Insects; basisOfRecord: PreservedSpecimen
- **Type status:** Other material. **Occurrence:** catalogNumber: SAM-DIP-A012470; recordedBy: V. Whitehead; sex: 1 female; lifeStage: Adult; otherCatalogNumbers: AAM-003042; **Taxon:** scientificNameID: urn:lsid:zoobank.org:act:CE05440A-6508-4A15-BC27-2A4EA9E52790; scientificName: *Namadytes vansoni* Hesse, 1969; family: Mydidae; genus: Namadytes; specificEpithet: vansoni; scientificNameAuthorship: Hesse, 1969; **Location:** country: Namibia; stateProvince: Kunene; locality: Duineveld No. 529, SW Khorixas; verbatimCoordinates: 20°47'00''S 014°38'00''E; decimalLatitude: -20.78333; decimalLongitude: 14.63333; **Identification:** identifiedBy: T. Dikow S. Leon; dateIdentified: 2012; **Event:** eventDate: 1978-05-14; **Record Level:** institutionCode: SAMC; collectionCode: Insects; basisOfRecord: PreservedSpecimen
- **Type status:** Other material. **Occurrence:** catalogNumber: SAM-DIP-A012470; recordedBy: V. Whitehead; sex: 1 female; lifeStage: Adult; otherCatalogNumbers: AAM-003044; **Taxon:** scientificNameID: urn:lsid:zoobank.org:act:CE05440A-6508-4A15-BC27-2A4EA9E52790; scientificName: *Namadytes vansoni* Hesse, 1969; family: Mydidae; genus: Namadytes; specificEpithet: vansoni; scientificNameAuthorship: Hesse, 1969; **Location:** country: Namibia; stateProvince: Kunene; locality: Duineveld No. 529, SW Khorixas; verbatimCoordinates: 20°47'00''S 014°38'00''E; decimalLatitude: -20.78333; decimalLongitude: 14.63333; **Identification:** identifiedBy: T. Dikow S. Leon; dateIdentified: 2012; **Event:** eventDate: 1978-05-14; **Record Level:** institutionCode: SAMC; collectionCode: Insects; basisOfRecord: PreservedSpecimen
- **Type status:** Other material. **Occurrence:** catalogNumber: SAM-DIP-A012470; recordedBy: V. Whitehead; sex: 1 female; lifeStage: Adult; otherCatalogNumbers: AAM-003051; **Taxon:** scientificNameID: urn:lsid:zoobank.org:act:CE05440A-6508-4A15-BC27-2A4EA9E52790; scientificName: *Namadytes vansoni* Hesse, 1969; family: Mydidae; genus: Namadytes; specificEpithet: vansoni; scientificNameAuthorship: Hesse, 1969; **Location:** country: Namibia; stateProvince: Kunene; locality: Duineveld No. 529, SW Khorixas; verbatimCoordinates: 20°47'00''S 014°38'00''E; decimalLatitude: -20.78333; decimalLongitude: 14.63333; **Identification:** identifiedBy: T. Dikow S. Leon; dateIdentified: 2012; **Event:** eventDate: 1978-05-14; **Record Level:** institutionCode: SAMC; collectionCode: Insects; basisOfRecord: PreservedSpecimen
- **Type status:** Other material. **Occurrence:** catalogNumber: SAM-DIP-A012468; recordedBy: V. Whitehead; sex: 1 female; lifeStage: Adult; otherCatalogNumbers: AAM-003045; **Taxon:** scientificNameID: urn:lsid:zoobank.org:act:CE05440A-6508-4A15-BC27-2A4EA9E52790; scientificName: *Namadytes vansoni* Hesse, 1969; family: Mydidae; genus: Namadytes; specificEpithet: vansoni; scientificNameAuthorship: Hesse, 1969; **Location:** country: Namibia; stateProvince: Kunene; locality: Duineveld No. 529, SW Khorixas; verbatimCoordinates: 20°47'00''S 014°38'00''E; decimalLatitude: -20.78333; decimalLongitude: 14.63333; **Identification:** identifiedBy: T. Dikow S. Leon; dateIdentified: 2012; **Event:** eventDate: 1978-05-14; **Record Level:** institutionCode: SAMC; collectionCode: Insects; basisOfRecord: PreservedSpecimen
- **Type status:** Other material. **Occurrence:** catalogNumber: SAM-DIP-A012474; recordedBy: V. Whitehead; sex: 1 male; lifeStage: Adult; otherCatalogNumbers: AAM-003040; **Taxon:** scientificNameID: urn:lsid:zoobank.org:act:CE05440A-6508-4A15-BC27-2A4EA9E52790; scientificName: *Namadytes vansoni* Hesse, 1969; family: Mydidae; genus: Namadytes; specificEpithet: vansoni; scientificNameAuthorship: Hesse, 1969; **Location:** country: Namibia; stateProvince: Kunene; locality: Rooiberg No. 517, W Khorixas; verbatimCoordinates: 20°27'00''S 014°35'00''E; decimalLatitude: -20.45; decimalLongitude: 14.58333; **Identification:** identifiedBy: T. Dikow S. Leon; dateIdentified: 2012; **Event:** eventDate: 1978-05-14; **Record Level:** institutionCode: SAMC; collectionCode: Insects; basisOfRecord: PreservedSpecimen
- **Type status:** Other material. **Occurrence:** catalogNumber: SAM-DIP-A012479; recordedBy: V. Whitehead; sex: 1 male; lifeStage: Adult; otherCatalogNumbers: AAM-003038; **Taxon:** scientificNameID: urn:lsid:zoobank.org:act:CE05440A-6508-4A15-BC27-2A4EA9E52790; scientificName: *Namadytes vansoni* Hesse, 1969; family: Mydidae; genus: Namadytes; specificEpithet: vansoni; scientificNameAuthorship: Hesse, 1969; **Location:** country: Namibia; stateProvince: Kunene; locality: Rooiberg No. 517, W Khorixas; verbatimCoordinates: 20°27'00''S 014°35'00''E; decimalLatitude: -20.45; decimalLongitude: 14.58333; **Identification:** identifiedBy: T. Dikow S. Leon; dateIdentified: 2012; **Event:** eventDate: 1978-05-14; **Record Level:** institutionCode: SAMC; collectionCode: Insects; basisOfRecord: PreservedSpecimen
- **Type status:** Other material. **Occurrence:** catalogNumber: SAM-DIP-A012473; recordedBy: V. Whitehead; sex: 1 female; lifeStage: Adult; otherCatalogNumbers: AAM-003039; **Taxon:** scientificNameID: urn:lsid:zoobank.org:act:CE05440A-6508-4A15-BC27-2A4EA9E52790; scientificName: *Namadytes vansoni* Hesse, 1969; family: Mydidae; genus: Namadytes; specificEpithet: vansoni; scientificNameAuthorship: Hesse, 1969; **Location:** country: Namibia; stateProvince: Kunene; locality: Rooiberg No. 517, W Khorixas; verbatimCoordinates: 20°27'00''S 014°35'00''E; decimalLatitude: -20.45; decimalLongitude: 14.58333; **Identification:** identifiedBy: T. Dikow S. Leon; dateIdentified: 2012; **Event:** eventDate: 1978-05-14; **Record Level:** institutionCode: SAMC; collectionCode: Insects; basisOfRecord: PreservedSpecimen
- **Type status:** Other material. **Occurrence:** catalogNumber: NMNW-H18283; sex: 1 female; lifeStage: Adult; otherCatalogNumbers: AAM-003012; **Taxon:** scientificNameID: urn:lsid:zoobank.org:act:CE05440A-6508-4A15-BC27-2A4EA9E52790; scientificName: *Namadytes vansoni* Hesse, 1969; family: Mydidae; genus: Namadytes; specificEpithet: vansoni; scientificNameAuthorship: Hesse, 1969; **Location:** country: Namibia; stateProvince: Karas; county: Namaland; locality: Mukorob No. 14; verbatimCoordinates: 25°29'00''S 018°10'00''E; decimalLatitude: -25.48333; decimalLongitude: 18.16667; **Identification:** identifiedBy: T. Dikow S. Leon; dateIdentified: 2012; **Event:** eventDate: 1974-04-12–1974-04-14; **Record Level:** institutionCode: NMNW; collectionCode: Insects; basisOfRecord: PreservedSpecimen
- **Type status:** Other material. **Occurrence:** catalogNumber: NMNW-H18282; sex: 1 male; lifeStage: Adult; otherCatalogNumbers: AAM-003010; **Taxon:** scientificNameID: urn:lsid:zoobank.org:act:CE05440A-6508-4A15-BC27-2A4EA9E52790; scientificName: *Namadytes vansoni* Hesse, 1969; family: Mydidae; genus: Namadytes; specificEpithet: vansoni; scientificNameAuthorship: Hesse, 1969; **Location:** country: Namibia; stateProvince: Karas; county: Namaland; locality: Mukorob No. 14; verbatimCoordinates: 25°29'00''S 018°10'00''E; decimalLatitude: -25.48333; decimalLongitude: 18.16667; **Identification:** identifiedBy: T. Dikow S. Leon; dateIdentified: 2012; **Event:** eventDate: 1974-04-12–1974-04-14; **Record Level:** institutionCode: NMNW; collectionCode: Insects; basisOfRecord: PreservedSpecimen
- **Type status:** Other material. **Occurrence:** catalogNumber: AAM-002987; recordedBy: J. Londt B. Stuckenberg; sex: 1 male; lifeStage: Adult; otherCatalogNumbers: AAM-002987; **Taxon:** scientificNameID: urn:lsid:zoobank.org:act:CE05440A-6508-4A15-BC27-2A4EA9E52790; scientificName: *Namadytes vansoni* Hesse, 1969; family: Mydidae; genus: Namadytes; specificEpithet: vansoni; scientificNameAuthorship: Hesse, 1969; **Location:** country: Namibia; stateProvince: Erongo; locality: Windhoek, 158 km W; verbatimCoordinates: 22°44'21''S 015°55'57''E; decimalLatitude: -22.73917; decimalLongitude: 15.9325; **Identification:** identifiedBy: T. Dikow S. Leon; dateIdentified: 2012; **Event:** eventDate: 1983-04-22; habitat: thornveld in dry river bed; **Record Level:** institutionCode: NMSA; collectionCode: Insects; basisOfRecord: PreservedSpecimen
- **Type status:** Other material. **Occurrence:** catalogNumber: AAM-002988; recordedBy: J. Londt B. Stuckenberg; sex: 1 male; lifeStage: Adult; otherCatalogNumbers: AAM-002988; **Taxon:** scientificNameID: urn:lsid:zoobank.org:act:CE05440A-6508-4A15-BC27-2A4EA9E52790; scientificName: *Namadytes vansoni* Hesse, 1969; family: Mydidae; genus: Namadytes; specificEpithet: vansoni; scientificNameAuthorship: Hesse, 1969; **Location:** country: Namibia; stateProvince: Erongo; locality: Windhoek, 158 km W; verbatimCoordinates: 22°44'21''S 015°55'57''E; decimalLatitude: -22.73917; decimalLongitude: 15.9325; **Identification:** identifiedBy: T. Dikow S. Leon; dateIdentified: 2012; **Event:** eventDate: 1983-04-22; habitat: thornveld in dry river bed; **Record Level:** institutionCode: NMSA; collectionCode: Insects; basisOfRecord: PreservedSpecimen
- **Type status:** Other material. **Occurrence:** catalogNumber: AAM-002826; recordedBy: R. Wharton; sex: 1 male; lifeStage: Adult; otherCatalogNumbers: AAM-002826; previousIdentifications: Namadytes prozeskyi by R. Wharton in 1979; **Taxon:** scientificNameID: urn:lsid:zoobank.org:act:CE05440A-6508-4A15-BC27-2A4EA9E52790; scientificName: *Namadytes vansoni* Hesse, 1969; family: Mydidae; genus: Namadytes; specificEpithet: vansoni; scientificNameAuthorship: Hesse, 1969; **Location:** country: Namibia; stateProvince: Erongo; locality: Gobabeb, Kuiseb River; verbatimCoordinates: 23°33'37''S 015°02'26''E; decimalLatitude: -23.56028; decimalLongitude: 15.04056; **Identification:** identifiedBy: T. Dikow S. Leon; dateIdentified: 2012; **Event:** eventDate: 1979-12-06; **Record Level:** institutionCode: NMSA; collectionCode: Insects; basisOfRecord: PreservedSpecimen

#### Description

**Male:** Fig. 9a, b.

Head: brown, in general lightly silver pubescent; width distinctly greater than thorax, interocular distance on vertex larger than at ventral eye margin, vertex between compound eyes ± horizontally straight, medially only slightly below dorsal eye margin, parafacial area about as wide as ½ the width of central facial gibbosity; facial gibbosity distinct, well-developed and discernible in lateral view; mystax white, densely covering entire facial gibbosity; frons not elevated, predominantly apubescent; vertex predominantly apubescent, only lateral margin grey pubescent; postgena lightly grey pubescent; setation: vertex white, frons white, ocp setae white, pocl macrosetae absent; ocellar triangle apubescent; proboscis brown, short, about ½ length of oral cavity; labellum small, as wide as prementum, as long as prementum, unsclerotized laterally; maxillary palpus cylindrical, light brown, minute.

Antenna: brown, scape and pedicel white setose dorsally and ventrally; postpedicel cylindrical in proximal ½, symmetrically bulbous in distal ½, ≥ 7.0 times as long as combined length of scape and pedicel, asetose; apical seta-like sensory element situated apically in cavity on postpedicel.

Thorax: brown, lightly grey pubescent; scutum uniformly brown, surface entirely smooth, apubescent, scutal setation comprised of long white setae with distinct rows of long dorsocentral setae and dense lateral scutal setae; dc setae pre- and postsuturally white, acr setae absent, lateral scutal setae white, npl setae 0, spal setae 0, pal setae 0; antepronotum dorso-medially with V-shaped indentation; postpronotal lobe light brown, grey pubescent; proepisternum, lateral postpronotum, and postpronotal lobe long white setose; scutellum apubescent, asetose, apical scutellar setae absent; mesopostnotum, anatergite, and katatergite lightly grey pubescent, mesopostnotum asetose, anatergite long white setose, katatergite long white setose; katatergite ± flat; anterior anepisternum white setose, supero-posterior anepisternum long white setose; posterior anepimeron long white setose, katepimeron long white setose; metanepisternum grey pubescent, asetose, metepimeron ± flat, yellow, grey pubescent, long white setose; infra-halter sclerite white setose.

Leg: light brown to brown, setation predominantly white; pro, mes, and met coxa lightly white pubescent, long white setose; met trochanter setose medially; femur light brown to brown, met femur evenly clubbed in distal 3/4, in distal ½ macrosetose, 1 antero-ventral and 1 postero-ventral row of macrosetae, postero-ventrally long white, erect setose proximally with setae arranged in distinct row; pro, mes, and met tibia straight, met tibia cylindrical, ventral keel absent, latero-posteriorly long white, erect setose with setae arranged in distinct row; pro and mes tarsomere 1 longer than tarsomere 2, but less than combined length of tarsomeres 2–3, met tarsomere 1 as long as combined length of tarsomeres 2–4; pulvillus well-developed, as long as well-developed claw, and as wide as base of claw; empodium absent.

Wing: length = 7.1–8.9 mm; hyaline throughout, veins brown, microtrichia absent; cells r_1_, r_4_, r_5_, m_3_, + cu*p* closed except r_5_ open; C terminates at junction with M_1_ (or M_1_+M_2_); R_4_ terminates in R_1_; R_5_ terminates in R_1_; stump vein (R_3_) at base of R_4_ present, short not reaching R_2_; R_4_ and R_5_ widest apart medially; r-m distinct, R_4__+__5_ and M_1_ apart, connected by crossvein; M_1_ straight at r-m (not curving anteriorly), M_1_ (or M_1_+M_2_) terminates in C; CuA_1_ and CuA_2_ split proximally to m-cu (cell m_3_ narrow proximally); M_3_+CuA_1_ do not terminate together in C; A_1_ undulating, cell a_1_ wide, A_1_ and wing margin further apart proximally than distally; alula well-developed; halter light brown.

Abdomen: yellow to brown; setation comprised of dense white setae, surface entirely smooth; T1 brown, T2 predominantly yellow with brown medially and antero-laterally, T3–7 yellow with brown antero-laterally; T1 and anterior ½ of T2 long white setose, remaining T short white setose; T predominantly apubescent; S1–7 light brown; S1–7 short white setose; S predominantly apubescent; T2–4 parallel-sided and not constricted waist-like; bullae on T2 black, transversely elongate, surface entirely smooth, T2 surface anterior to bullae smooth.

♂ terminalia: Fig. 10.

**Female:** Fig. 9c, d.

Head: mystax white, covering entire facial gibbosity, sparse; pocl macrosetae white.

Antenna: postpedicel ≥ 5.0–≥ 6.0 times as long as combined length of scape and pedicel.

Thorax: scutum predominantly brown pubescent, narrow sublateral stripes (wider anteriorly) and lateral and posterior margins grey pubescent, scutal setation comprised of scattered short white setae; scutellum grey pubescent proximally, apubescent distally; supero-posterior anepisternum short white setose; posterior anepimeron short white setose, katepimeron short white setose; metepimeron light brown or yellow, grey pubescent, short white setose.

Leg: setation yellow; met femur ± cylindrical only slightly wider than pro and mes femur, postero-ventrally regular setose only; met tibia latero-posteriorly regular setose only; pulvillus reduced, half length of well-developed claw.

Wing: length = 10.9–14.2 mm; hyaline throughout, slightly brown stained along veins.

Abdomen: setation comprised of sparsely scattered short yellow setae; T1–5 brown with yellow posterior margin, T6 brown (sometimes yellow posteriorly), T7 brown; T1–7 sparsely yellow setose; S1–7 brown; S1–7 sparsely short yellow setose.

♀ genitalia: 8–9 acanthophorite spines per plate.

#### Diagnosis

This large species (wing length in males 7.1–8.9 mm and in females 10.9–14.2 mm) is distinguished from congeners by the wing venation in that cell r_5_ is open and therefore M_1_ terminates in C (and not in R_1_), the predominantly apubescent vertex, the short proboscis that is only about half the length of the oral cavity, the long white setose anatergite, and the setose katepimeron.

#### Distribution

Namibia (Erongo, Karas, Kunene) (Fig. 6).

#### Biology

##### Flight behavior

Females of this species (as *Namadytes prozeskyi* syn. nov.) were observed by Wharton (1982) to have a hop-like flight in contrast to the low-flying males, which show the characteristic rapid gliding flight behavior in order to locate females for mating. A similar observation has recently been made for *Namibimydas psamminos* Dikow, 2012 (Dikow 2012, p. 92).

##### Oviposition

Females usually oviposited in shallow depressions, such as hoof prints and in particular on the lip of these prints, in the sandy Kuiseb river bed and followed a Mydidae-characteristic oviposition sequence of sand-ovipositing species (for details see Wharton 1982, p. 149). The insertion of the abdomen into the sand took about 9 seconds while the egg-laying with buried abdomen lasted for 6 seconds. The eggs were orange, hyaline, and pear-shaped and measured 2 x 1 mm (length x maximum width).

##### Habitat

*Namadytes vansoni* has been collected in riparian vegetation along a dry river bed, in thornveld in a dry river bed, and on barren gravel plains.

#### Discussion

This species exhibits substantial intra-specific variation (Figs 9, 11) and is the most variable species. However, it is also the species known from the most specimens (61 specimens in total) and has the largest geographic range. Prior to this study, *Namadytes vansoni* is only known from the sole female holotype (Fig. 9c, d). Only through female and male specimens collected during a single collecting event is it possible to associate both sexes and hence appreciate the pronounced sexual dimorphism (Figs 9a, c, 11). Hesse (1969) hints in the description of *Namadytes prozeskyi*, which is also only known from a single female holotype, at the similarity to *Namadytes vansoni* and while he provides quite a few minor differences, we attribute these to intra-specific variation. With the increased number of specimens available in our study, we cannot differentiate the two species and therefore synonymize *Namadytes prozeskyi* (described on page 282) with *Namadytes vansoni* (described on page 280) by page priority.

#### Type locality

Namibia: Karas: Seeheim (26°48'53''S, 017°47'57''E) (Fig. 6).

#### Biodiversity hotspot

Not known to occur in any of the southern African biodiversity hotspots (Cape Floristic Region, Maputaland-Pondoland-Albany, or Succulent Karoo) (Fig. 6).

## Identification Keys

### Key to *Namadytes* species

**Table:** 

1	Anatergite setose; cell r_5_ open (M_1+2_ terminating into C); proboscis about ½ length of oral cavity; katepimeron setose	* Namadytes vansoni *
–	Anatergite asetose; cell r_5_ closed (M_1+2_ terminating into R_1_); proboscis short, less than ½ length of oral cavity; katepimeron asetose	2
2	Infra-halter sclerite (ventral to halter base and posterior to metathoracic spiracle) with only very few white setae (Fig. 12b); metathoracic femur with only short, appressed yellowish setae dorsally; postpedicel only about 4 times as long as scape and pedicel combined; mystacal setae white or yellow and only sparsely covering facial gibbosity; smaller flies: male wing length = 6.6–8.1 mm, female wing length = 10.6-11.9 mm	* Namadytes cimbebasiensis *
–	Infra-halter sclerite long white setose (Fig. 12c); metathoracic femur with long, erect white setae dorsally; postpedicel about 7 times as long as scape and pedicel combined; mystacal setae white and densely covering facial gibbosity; larger flies: male wing length = 9.6–12.2 mm, females unknown	* Namadytes maculiventris *

There is considerable sexual dimorphism and the setation, for example on the anatergites, is always easier to observe in males. Intraspecific variation in the abdominal coloration, especially in females, is likewise substantial.

## Discussion

### Morphological characteristics

*Namadytes* species exhibit two remarkable morphological characteristics unknown in any other Mydidae genus.

- The antepronotum is anteriorly not entire as in all Mydidae, but has a V-shaped indentation medially that is easily visible (Fig. 12a).
- A small sclerite ventral to the halter and posterior to the metathoracic spiracle, here termed the infra-halter sclerite (Fig. 12b, c), is unique within Mydidae. This sclerite is usually long, densely white setose, but only sparsely white setose *Namadytes cimbebasiensis* males and sometimes even asetose in females of this species.

### Biology

The knowlegde of the biology of *Namadytes* is very scarce as there is hardly any information on habitat preferences or flight behavior available on collecting labels. However, Wharton (1982) provided some information on *Namadytes vansoni* (as *Namadytes prozeskyi* syn. nov.) based on his year-long study of Mydidae at the Gobabeb Research and Training Centre in the central Namib Desert, which is summarized under the biology section of that species.

Although not directly observed by Wharton, he suggests that mating with teneral or very young females might occur in *Namadytes* as well and is an important adaptation for short-lived species in desert environments (Wharton 1982, p. 149).

### Seasonal incidence

*Namadytes* has primarily been collected during February through June, during the Southern Hemisphere late summer to early winter, as well as in October (Southern Hemisphere spring) (Table 1). *Namadytes cimbebasiensis*, ranging from north-western South Africa to south-western Namibia (Fig. 6) occurs during March–May, while *Namadytes maculiventris*, the species with the smallest geographic range restricted to southern Namibia (the type locality is on the South African bank of the Orange River, Fig. 6), occurs in February–March and October, and *Namadytes vansoni*, the most abundantly collected species distributed throughout much of Namibia (Fig. 6), occurs in February–June and October. It is interesting to note that the October records represent the collecting events of the holotypes of *Namadytes pallidus* (junior synonym of *Namadytes maculiventris*) and *Namadytes prozeskyi* (junior synonym of *Namadytes vansoni*).

Wharton (1982) studied the Mydidae fauna at the Gobabeb Research and Training Centre, which is located in the center of the species' north-south range in the central Namib desert (23°33'37''S 015°02'26''E), for an entire year and collected *Namadytes vansoni* (identified as *Namadytes prozeskyi*) between April through June (Southern Hemisphere fall to early winter). The southernmost occurrence record of *Namadytes vansoni* is from Rotegab farm No. 95 (27°20'00''S 018°25'00''E) collected in April, which is only some 85 km (straight line) south-east of the type locality Seeheim (26°48'53''S 017°47'57''E) where the holotype was collected in October. The northernmost occurrence records around Khorixas in southern Kunene Region are all from May (Southern Hemisphere late fall).

*Namadytes* has not been sampled during the hottest months of the year, *i.e.*, December–January, but has been collected in the cooler spring and late summer to early winter months. However, when one takes a closer look at the temperature and rainfall patterns at specific localities, taken from World Weather Online (http://www.worldweatheronline.com), a different picture emerges (see Table 2 for details). At Gobabeb, the hottest months are March–April (Suppl. material 4) and the wettest are February–April and June (Suppl. material 5) coinciding with the occurrence of *Namadytes vansoni*. Further north at the northernmost distribution of this species around Khorixas, where it occurs in May, the average temperature is higher in May than in any other month between February–August (Suppl. materials 8, 9). At the type locality of *Namadytes vansoni* (Seeheim near Keetmanshoop), the holotype was collected during May, which is one of the coolest months during the year (Suppl. materials 6, 7). *Namadytes maculiventris* occurs at Aus and Vioolsdrift during the hotter months of the year (February–March, Suppl. materials 2, 3, 14, 15) and during one of the median temperature months (October) near Keetmanshoop. *Namadytes cimbebasiensis* occurs in March–April during a time of high temperature (see Suppl. materials 12, 13 for nearest available weather station in Upington) and during a cooler month (May) at its type locality near Maltahöhe (Suppl. materials 10, 11). Despite not occurring during the peak of the summer, locally *Namadytes* does occur during the hottest months as well as cooler time sod the year.

These data provide a glimpse at the temperature tolerance within species as, for example, *Namadytes maculiventris* occurs at Vioolsdrift with an average high temperature of 19 °C (March) and near Keetmanshoop with an average high temperature of 32 °C (October).

### Biodiversity hotspots

The biodiversity hotspots *sensu Conservation International* (Myers et al. 2000) are areas of high plant endemism in which the habitat has been destroyed to a considerable extant and which are under threat of more destruction. Evaluating the presence/absence of Diptera species in these priority areas earmarked for conservation can determine whether these species will also be preserved when funding is made available for their protection (*e.g.*, Dikow et al. 2009). In a number of recent taxonomic revisions on Mydidae (Dikow 2010, Dikow 2012), it was discovered that several species within the studied genera are endemic to a particular hotspot, occur within a hotspot (but are not endemic to it), or occur only outside of these hotspots. Of the three species dealt with in this contribution, none occur or are endemic to any biodiversity hotspot *sensu Conservation International* (see Fig. 6). Therefore, species of *Namadytes* would not benefit when only the biodiversity hotspots will receive funding for conservation and future habitat destruction and lack of conservation initiatives might have an effect on local populations.

## Supplementary Material

Supplementary material 1 Natural-language species descriptions in SDD format Data type: morphological
Brief description: The XML file includes the natural-language species descriptions in SDD (Structure of Descriptive Data) format.
File: oo_6075.sdd Dikow, T. and Leon, S.

Supplementary material 2 Average annual temperature at Aus Data type: image, graph
Brief description: Average temperature Aus
File: oo_4629.png World Weather Online

Supplementary material 3 Average annual rainfall at Aus Data type: image, graph
Brief description: Average rainfall Aus
File: oo_4630.png World Weather Online

Supplementary material 4 Average annual temperature at Gobabeb Data type: image, graph
Brief description: Average temperature Gobabeb
File: oo_4616.png World Weather Online

Supplementary material 5 Average annual rainfall at Gobabeb Data type: image, graph
Brief description: Average rainfall Gobabeb
File: oo_4617.png World Weather Online

Supplementary material 6 Average annual temperature at Keetmanshoop Data type: image, graph
Brief description: Average temperature Keetmanshoop
File: oo_4618.png World Weather Online

Supplementary material 7 Average annual rainfall at Keetmanshoop Data type: image, graph
Brief description: Average rainfall Keetmanshoop
File: oo_4619.png World Weather Online

Supplementary material 8 Average annual temperature at Khorixas Data type: image, graph
Brief description: Average temperature Khorixas
File: oo_4620.png World Weather Online

Supplementary material 9 Average annual rainfall at Khorixas Data type: image, graph
Brief description: Average rainfall Khorixas
File: oo_4621.png World Weather Online

Supplementary material 10 Average annual temperature at Maltahöhe Data type: image, graph
Brief description: Average temperature Maltahöhe
File: oo_4626.png World Weather Online

Supplementary material 11 Average annual rainfall at Maltahöhe Data type: image, graph
Brief description: Average rainfall Maltahöhe
File: oo_4627.png World Weather Online

Supplementary material 12 Average annual temperature at Upington Data type: image, graph
Brief description: Average temperature Upington
File: oo_4622.png World Weather Online

Supplementary material 13 Average annual rainfall at Upington Data type: image, graph
Brief description: Average rainfall Upington
File: oo_4623.png World Weather Online

Supplementary material 14 Average annual temperature at Vioolsdrift Data type: image, graph
Brief description: Average temperature Vioolsdrift
File: oo_4624.png World Weather Online

Supplementary material 15 Average annual rainfall at Vioolsdrift Data type: image, graph
Brief description: Average rainfall Vioolsdrift
File: oo_4625.png World Weather Online

XML Treatment for Namadytes

XML Treatment for Namadytes cimbebasiensis

XML Treatment for Namadytes maculiventris

XML Treatment for Namadytes vansoni

## Figures and Tables

**Figure 2a.**
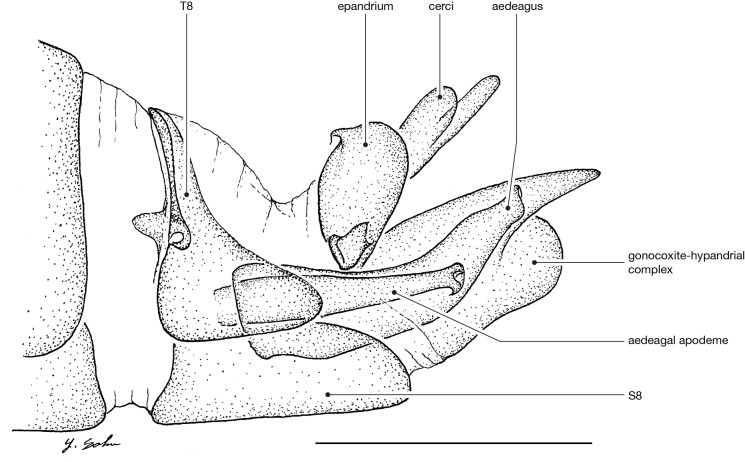
lateral

**Figure 2b.**
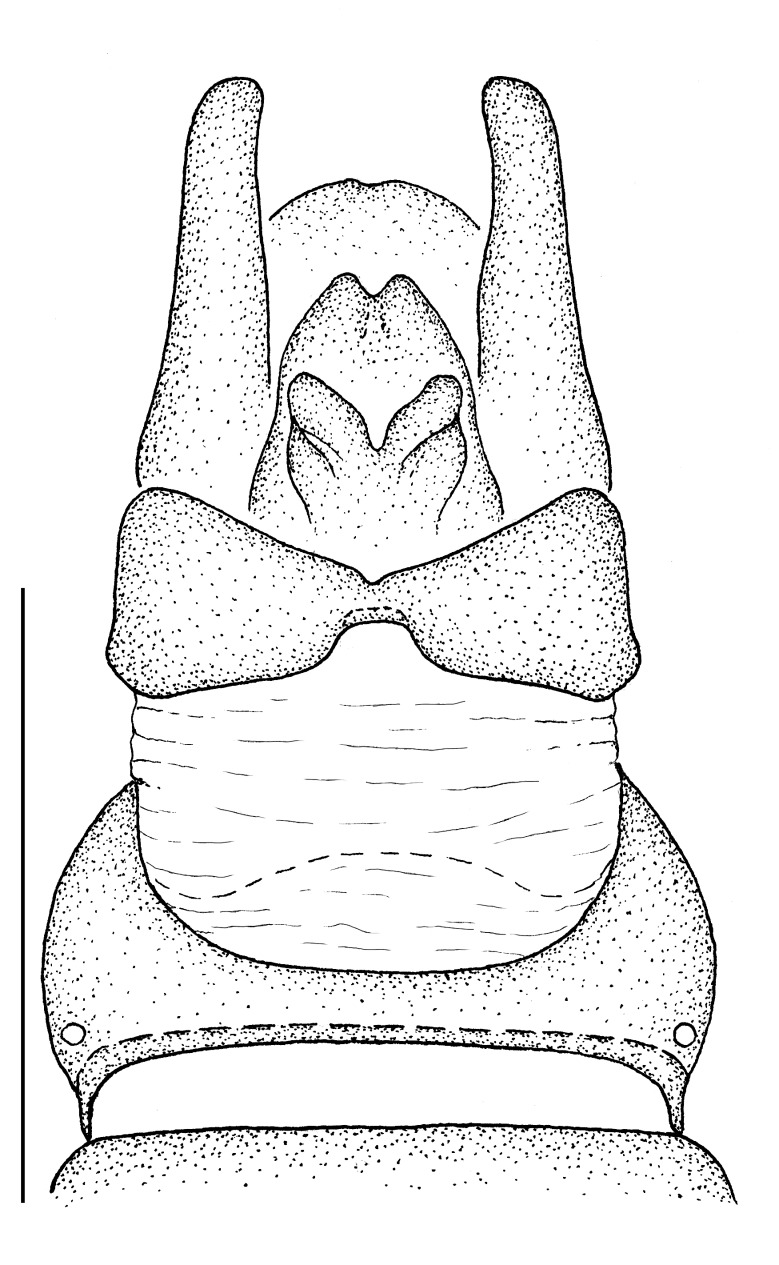
dorsal

**Figure 2c.**
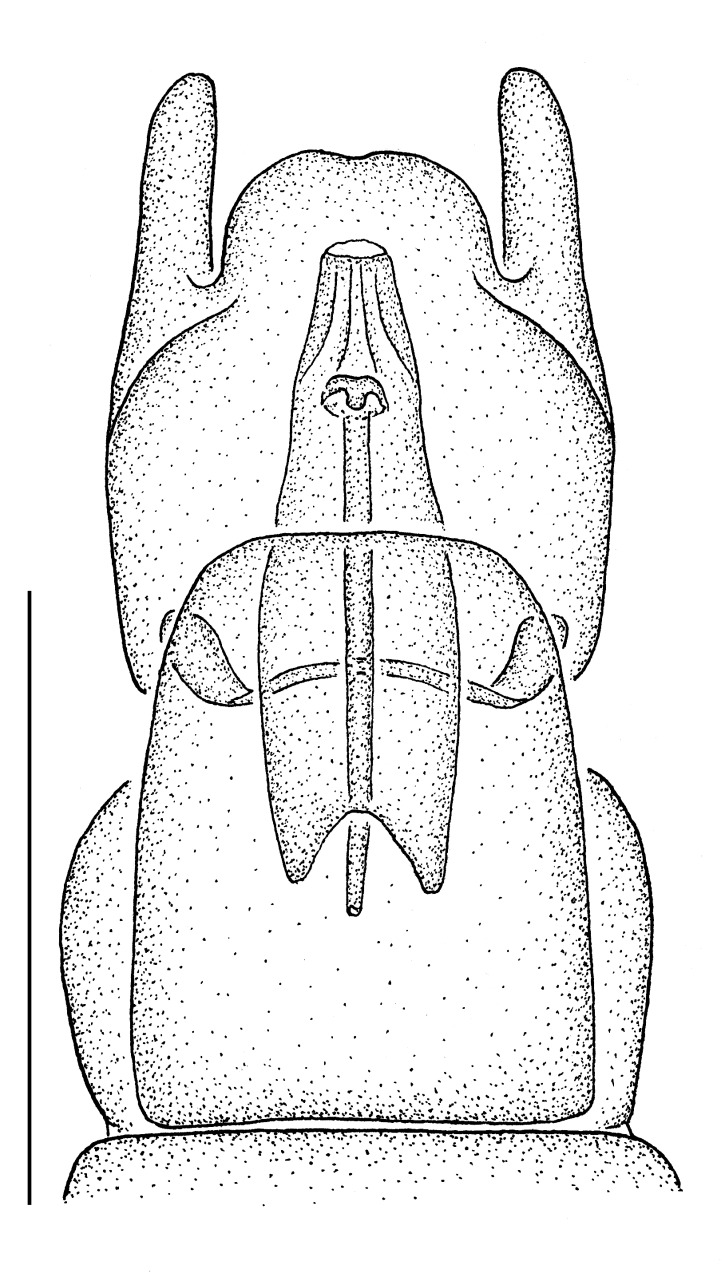
ventral

**Figure 5a.**
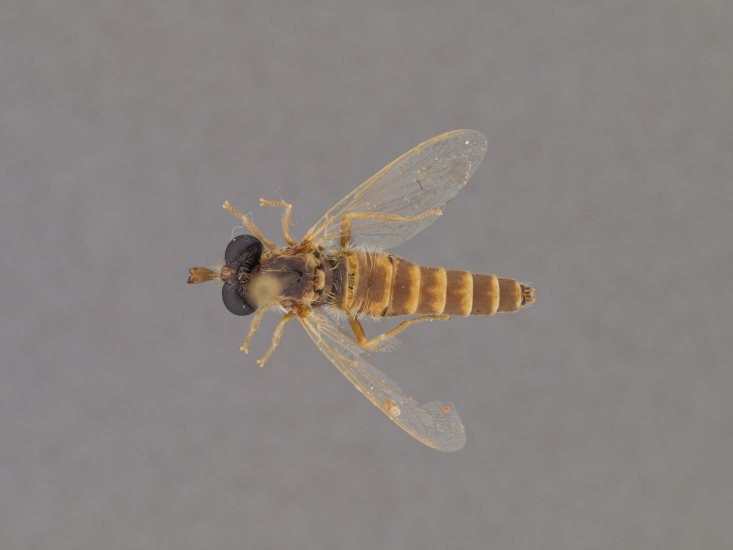
♂ (AAM-003021) dorsal (Morphbank #835209)

**Figure 5b.**
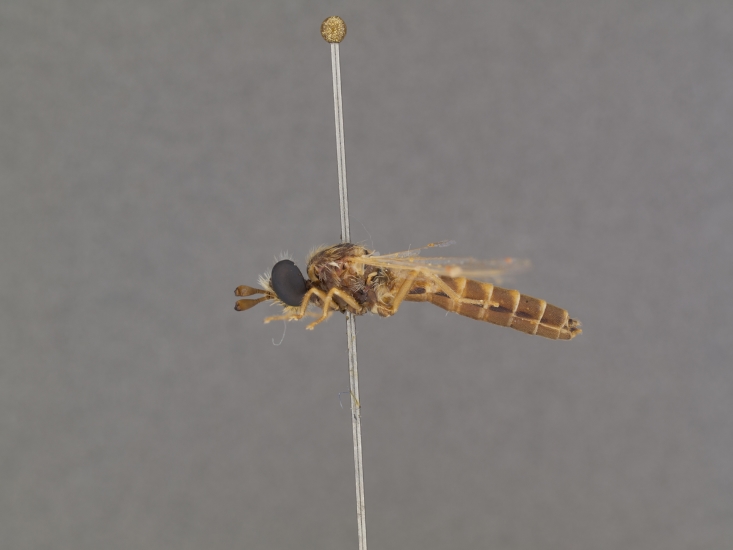
♂ (AAM-003021) lateral (#835211)

**Figure 5c.**
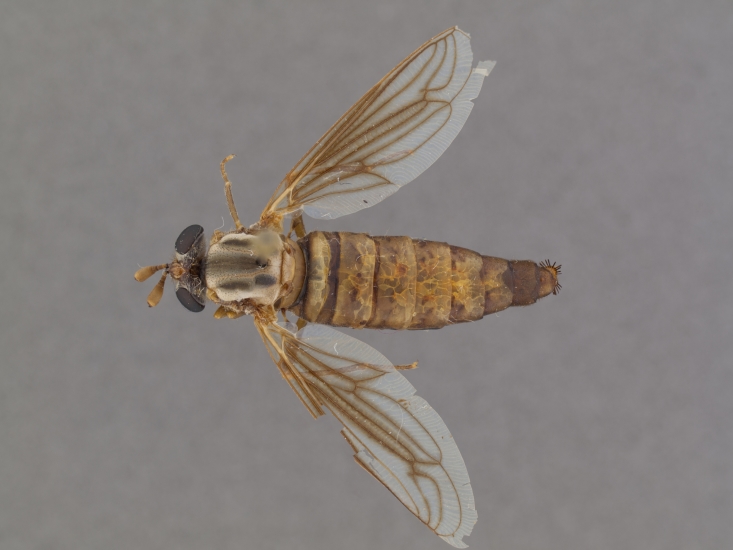
♀ (AAM-003000) dorsal (#835216)

**Figure 5d.**
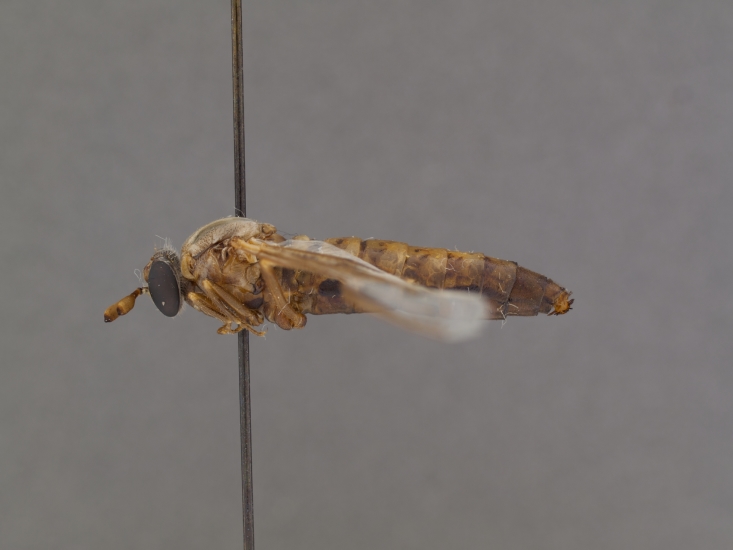
♀ (AAM-003000) lateral (#835217)

**Figure 7a.**
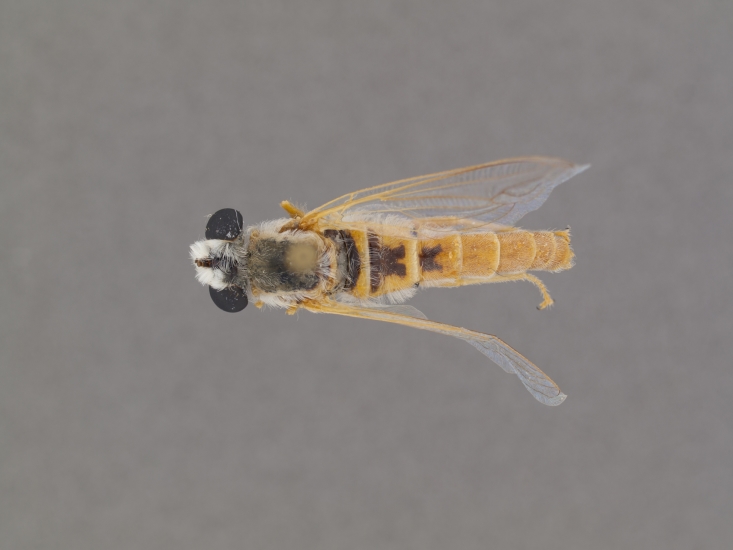
dorsal (Morphbank #835202)

**Figure 7b.**
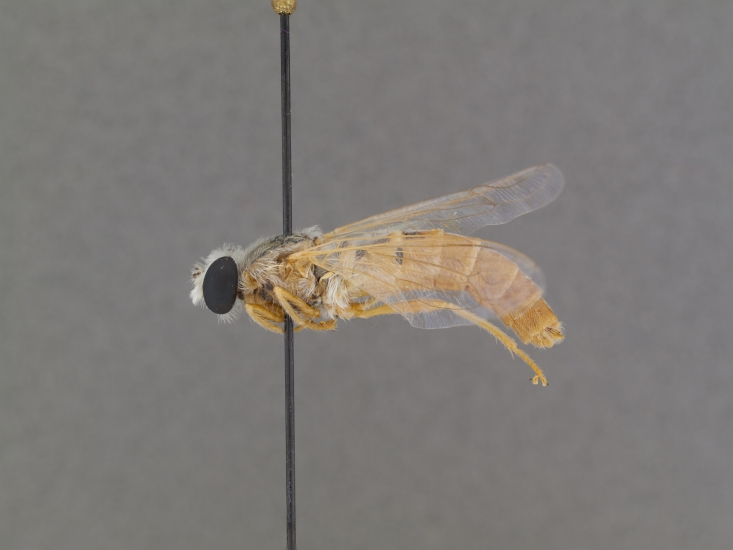
lateral (#835204)

**Figure 8a.**
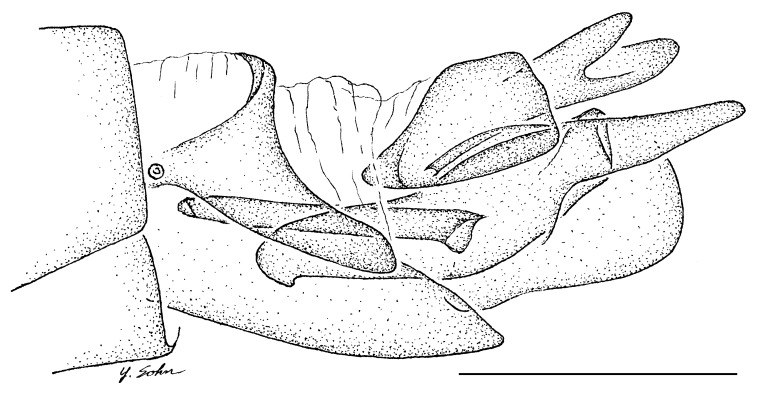
lateral

**Figure 8b.**
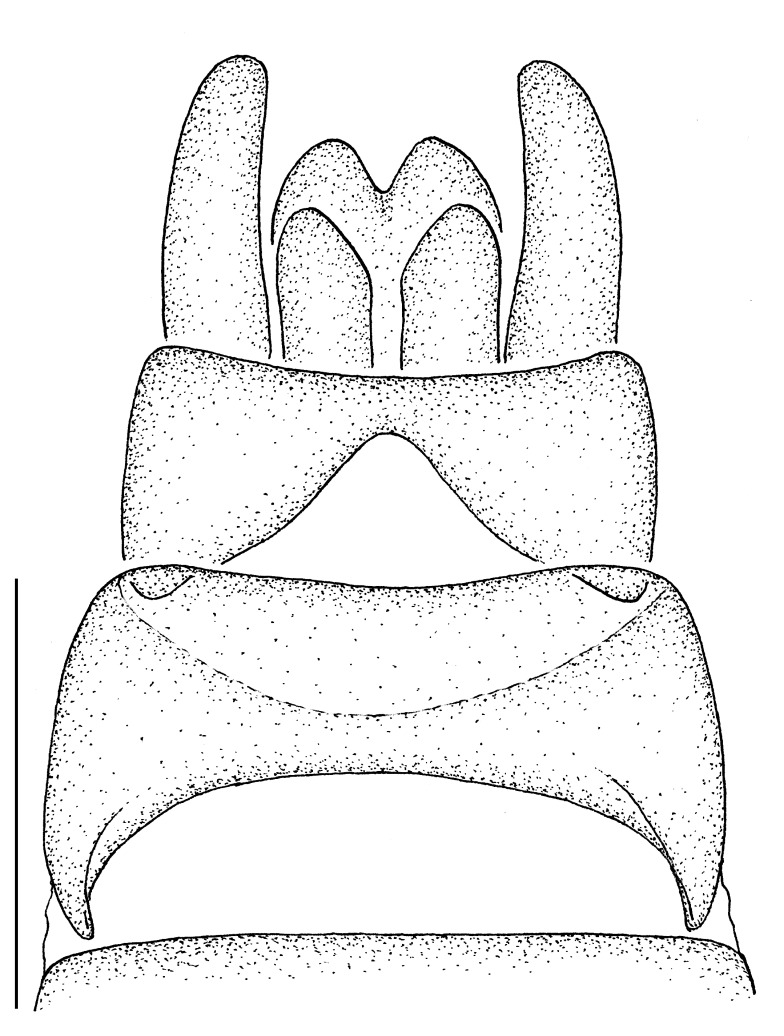
dorsal

**Figure 8c.**
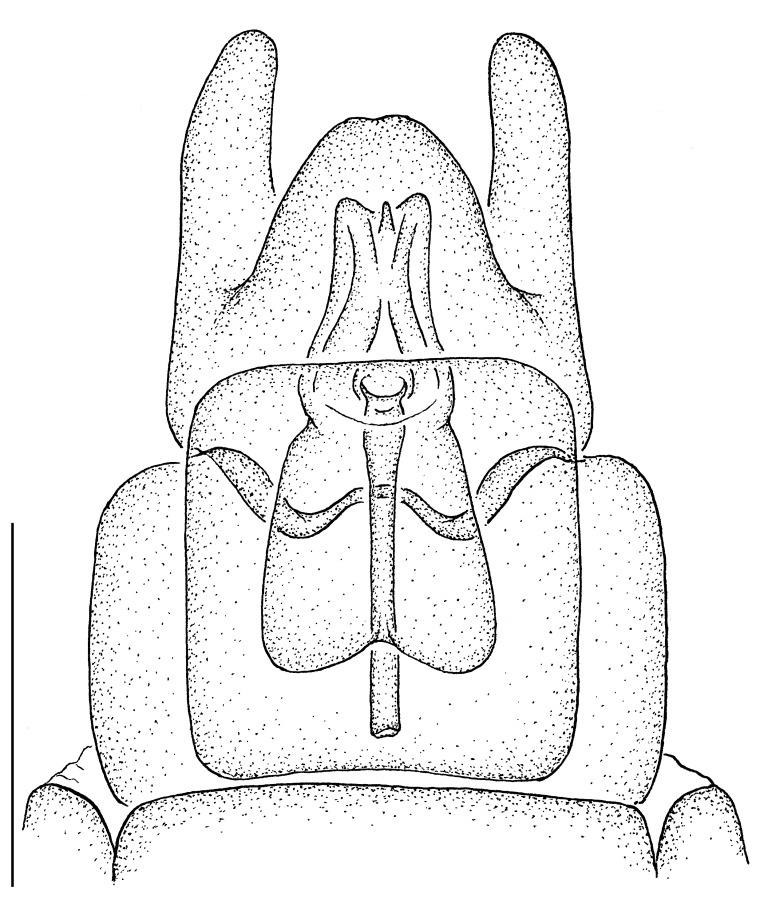
ventral

**Figure 9a.**
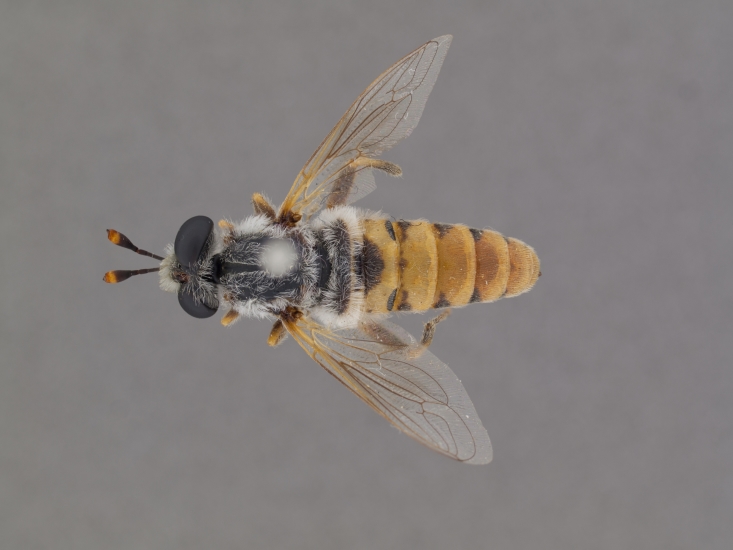
♂ (AAM-002988, head rotated 180˚) dorsal (Morphbank #835189)

**Figure 9b.**
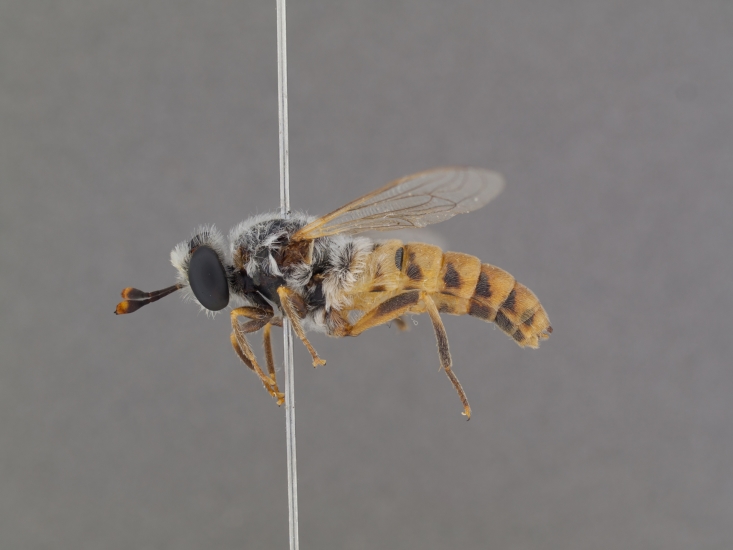
♂ (AAM-002988, head rotated 180˚) lateral (#835191)

**Figure 9c.**
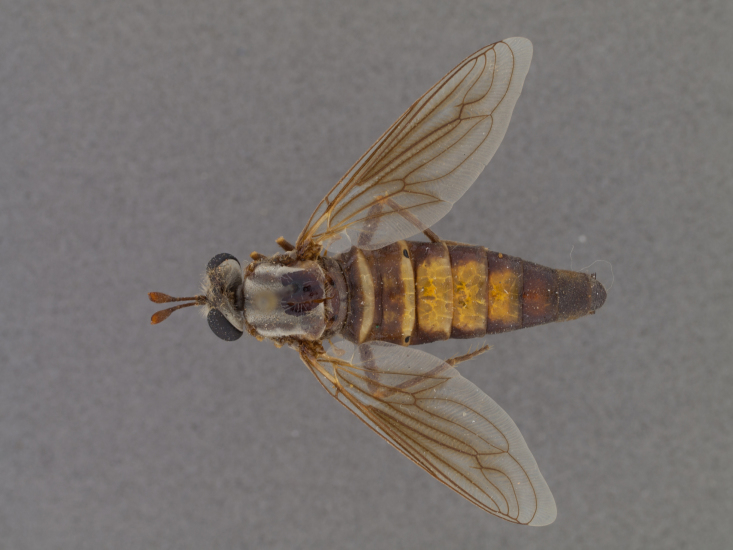
♀ (AAM-000456, Holotype) dorsal (#835195)

**Figure 9d.**
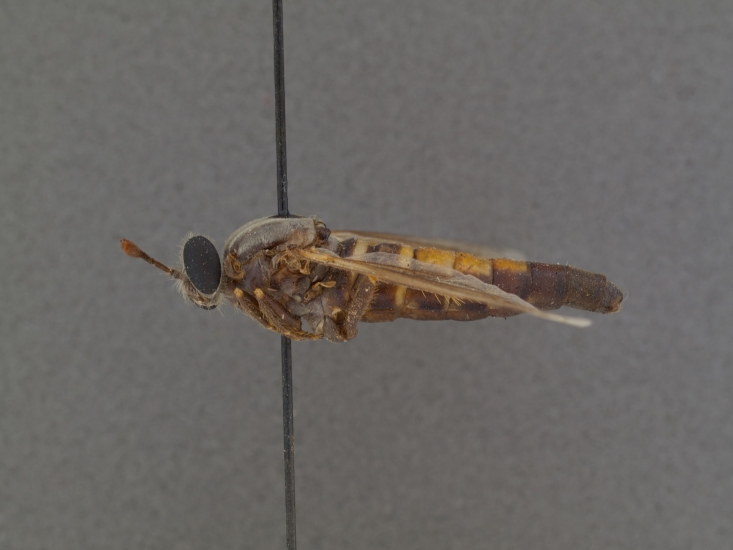
♀ (AAM-000456, Holotype) lateral (#835197)

**Figure 10a.**
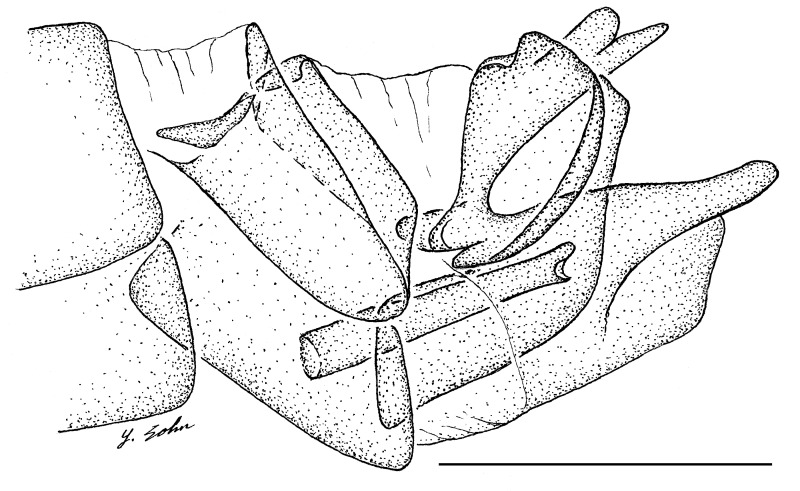
lateral

**Figure 10b.**
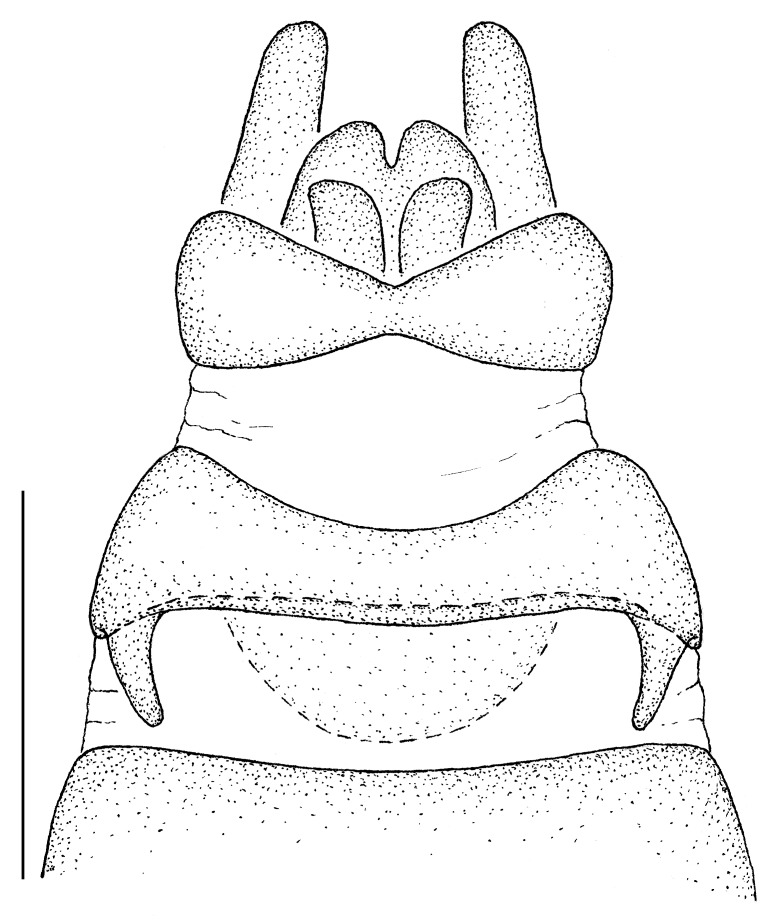
dorsal

**Figure 10c.**
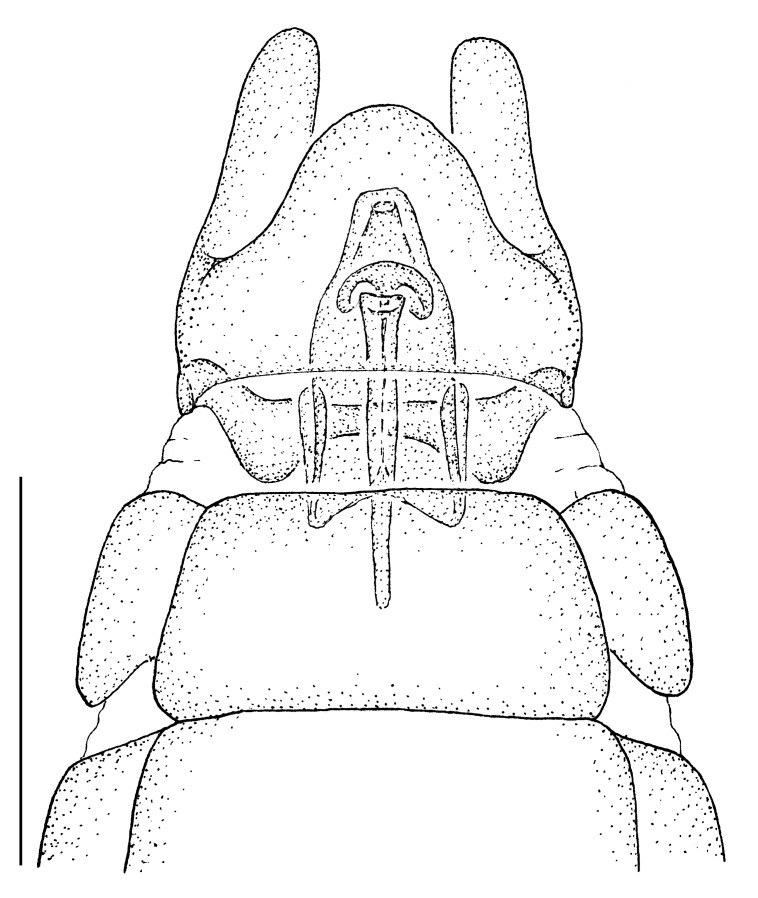
ventral

**Figure 10d.**
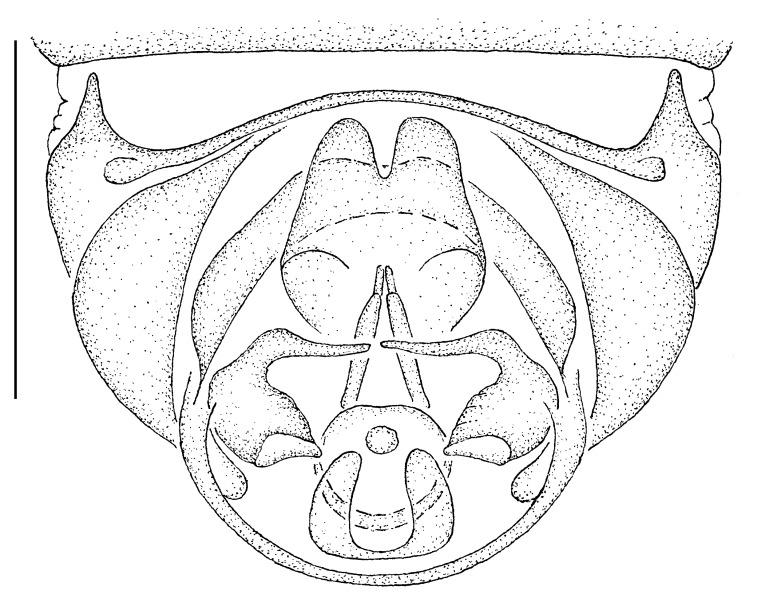
posterior

**Figure 11a.**
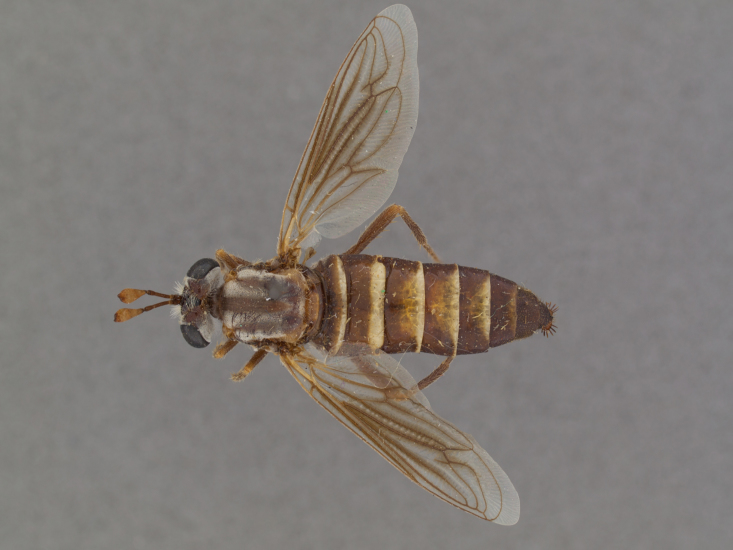
♀ holotype of *Namadytes prozeskyi* syn. nov. (TMSA-Dip35, Morphbank #835295)

**Figure 11b.**
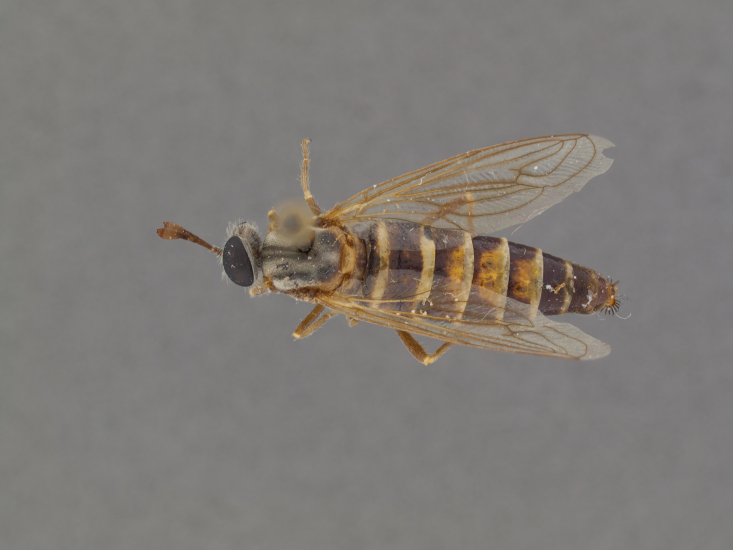
♀ (AAM-002924, #835303)

**Figure 11c.**
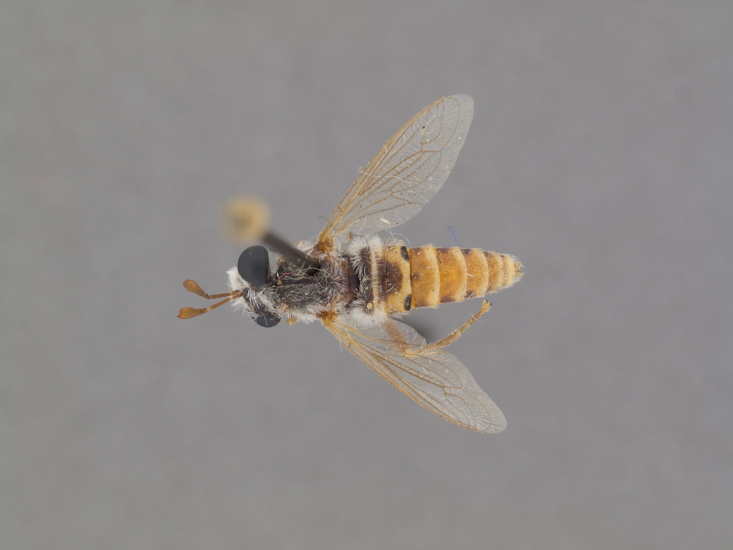
♂ (AAM-002826, #835306)

**Figure 11d.**
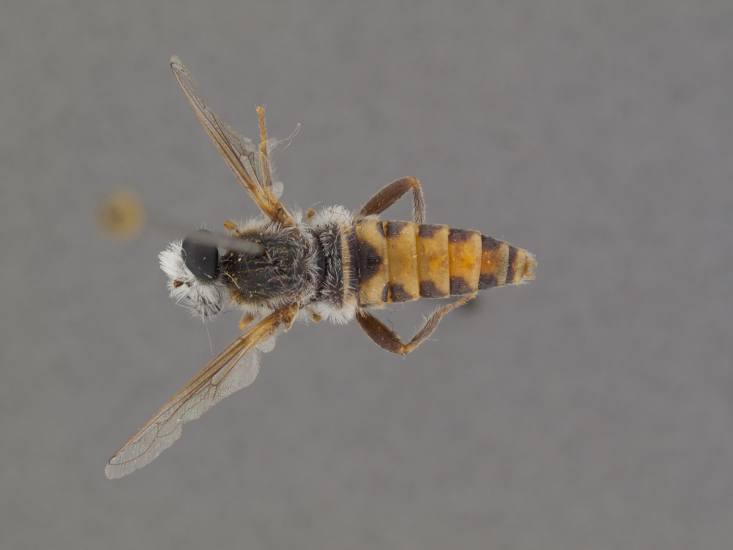
♂ (AAM-003046, #835299)

**Figure 12a.**
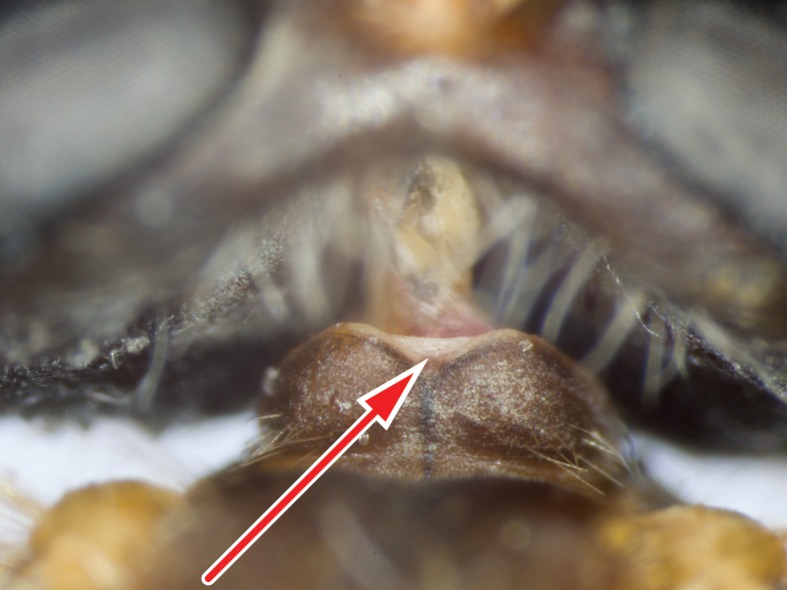
Antepronotum with V-shaped indentation of *Namadytes cimbebasiensis* in dorsal view (♂ AAM-003021, Morphbank #835220).

**Figure 12b.**
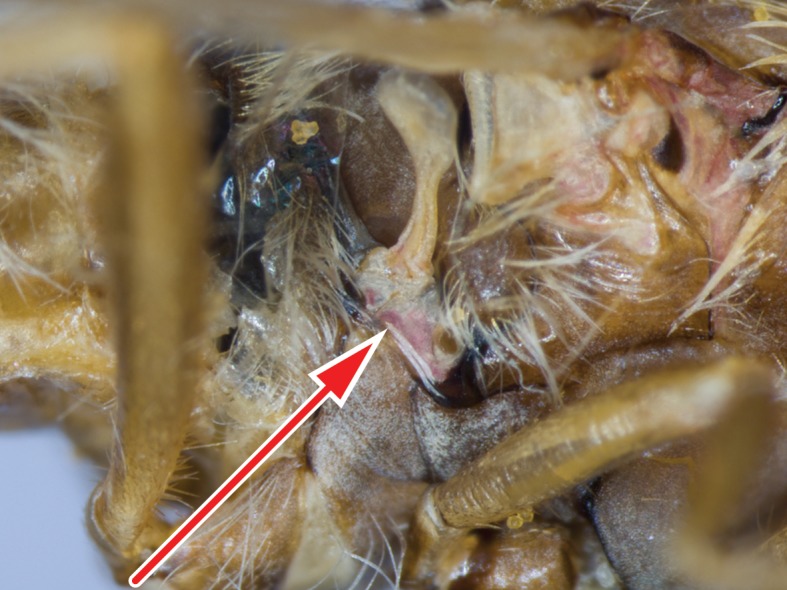
Sparsely setose infra-halter sclerite of *Namadytes cimbebasiensis* in lateral view (♂ AAM-003021, #835222).

**Figure 12c.**
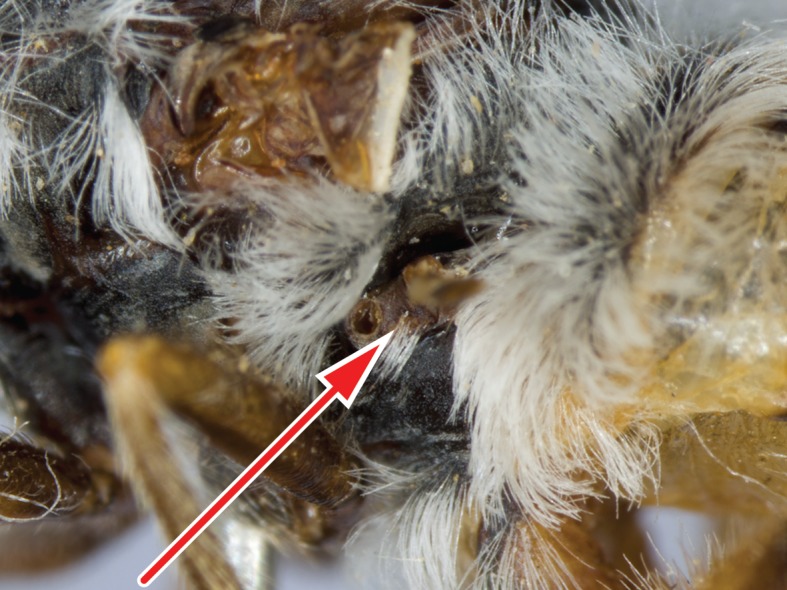
Densely setose infra-halter sclerite of *Namadytes vansoni* in lateral view (♂ AAM-002899, #835290)

**Table 1.** Seasonal incidence of *Namadytes* species.

Month	*Namadytes cimbebasiensis*	*Namadytes maculiventris*	*Namadytes vansoni*
January	–	–	–
February	–	√	√
March	√	√	√
April	√	–	√
May	√	–	√
June	–	–	√
July	–	–	–
August	–	–	–
September	–	–	–
October	–	√	√
November	–	–	–
December	–	–	–

**Table 2.** *Namadytes* species and the temperature and occurrence at selected localities. Temperature data from WorldWeatherOnline. Note that selected localities are places for which temperature data are available, for example, *Namadytes cimbebasiensis* has not been collected at Upington, but north, north-east, and north-west of it.

Month	*Namadytes cimbebasiensis*				*Namadytes maculiventris*						*Namadytes vansoni*					
	Maltahöhe		Upington		Aus		Keetman­shoop		Vioolsdrift		Gobabeb		Keetman­shoop		Khorixas	
	°C	coll.	°C	coll.	°C	coll.	°C	coll.	°C	coll.	°C	coll.	°C	coll.	°C	coll.
January	34	–	37	–	24	–	36	–	20	–	30	–	36	–	31	–
February	33	–	37	–	24	✓	35	–	20	–	30	✓	35	–	27	–
March	31	–	34	✓	24	–	34	–	19	✓	32	✓	34	–	24	–
April	28	–	30	✓	23	–	30	–	19	–	31	✓	30	✓	24	✓
May	25	✓	26	–	22	–	26	–	20	–	29	–	26	✓	28	✓
June	21	–	23	–	22	–	23	–	19	–	27	✓	23	–	26	–
July	22	–	23	–	21	–	23	–	18	–	26	–	23	–	26	–
August	24	–	25	–	19	–	25	–	17	–	24	–	25	–	25	–
September	27	–	29	–	20	–	29	–	17	–	26	–	29	–	29	–
October	30	–	32	–	21	–	32	✓	18	–	28	–	32	–	30	–
November	32	–	34	–	22	–	34	–	18	–	30	–	34	–	32	–
December	33	–	37	–	23	–	36	–	19	–	29	–	36	–	30	–
