# Supplementary figures and images for: Review of the genus Namadytes Hesse, 1969 (Insecta: Diptera: Mydidae: Syllegomydinae)
Source: Biodivers Data J. 2014 Mar 10;(2):e1071. doi: 10.3897/BDJ.2.e1071 (PMC4031440; doi:10.3897/BDJ.2.e1071)

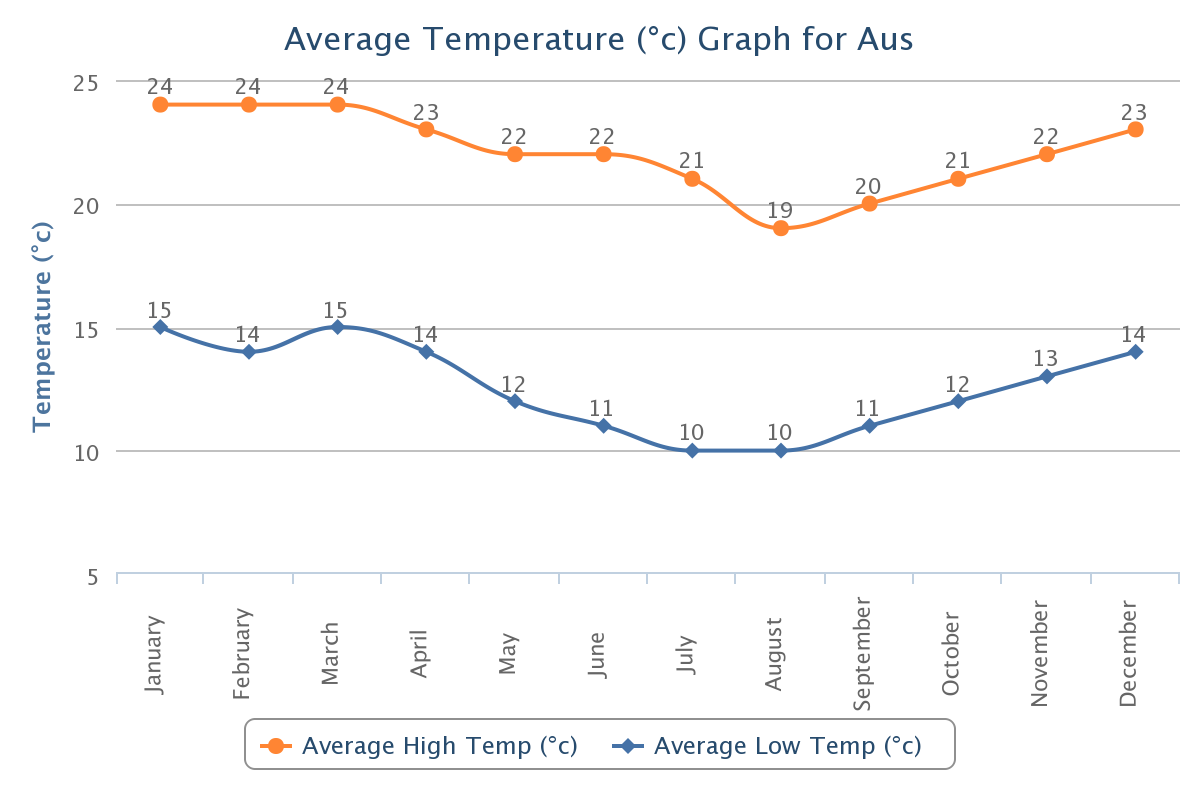

Supplement: Supplementary material 2 — Average annual temperature at Aus [file biodiversity_data_journal-2-e1071-s002.png]

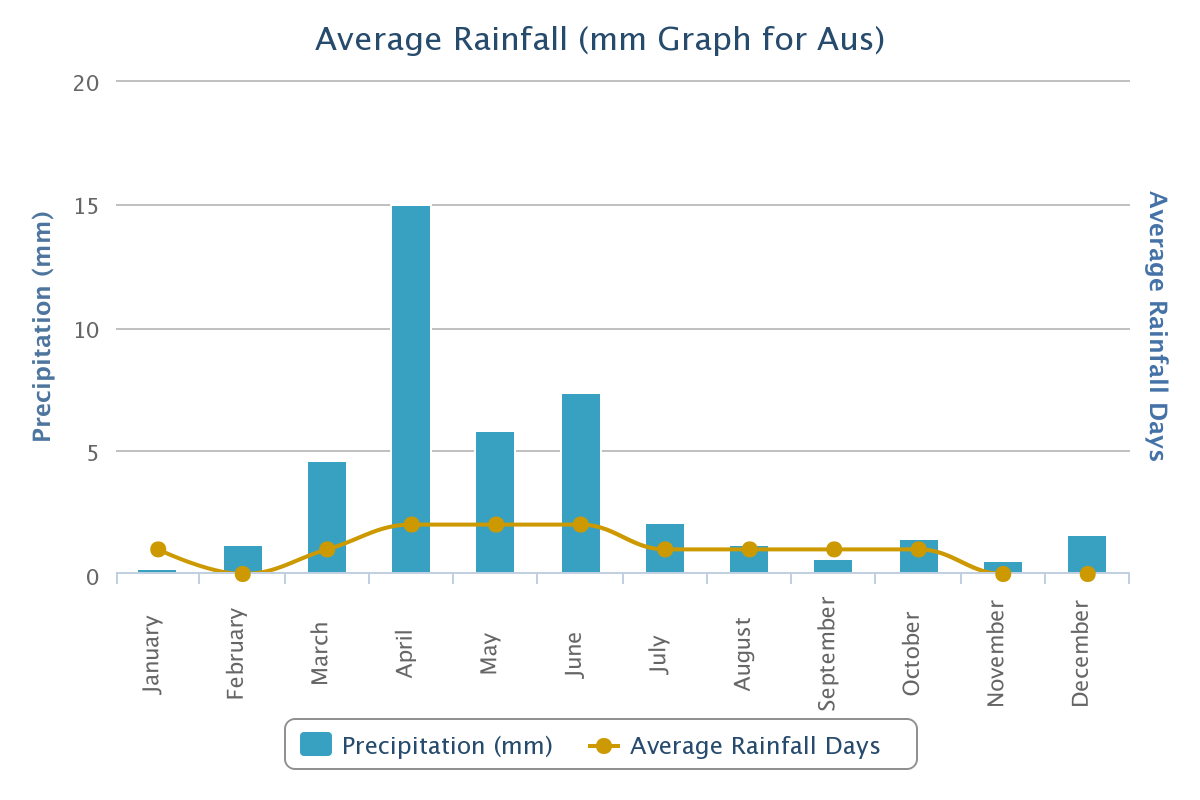

Supplement: Supplementary material 3 — Average annual rainfall at Aus [file biodiversity_data_journal-2-e1071-s003.png]

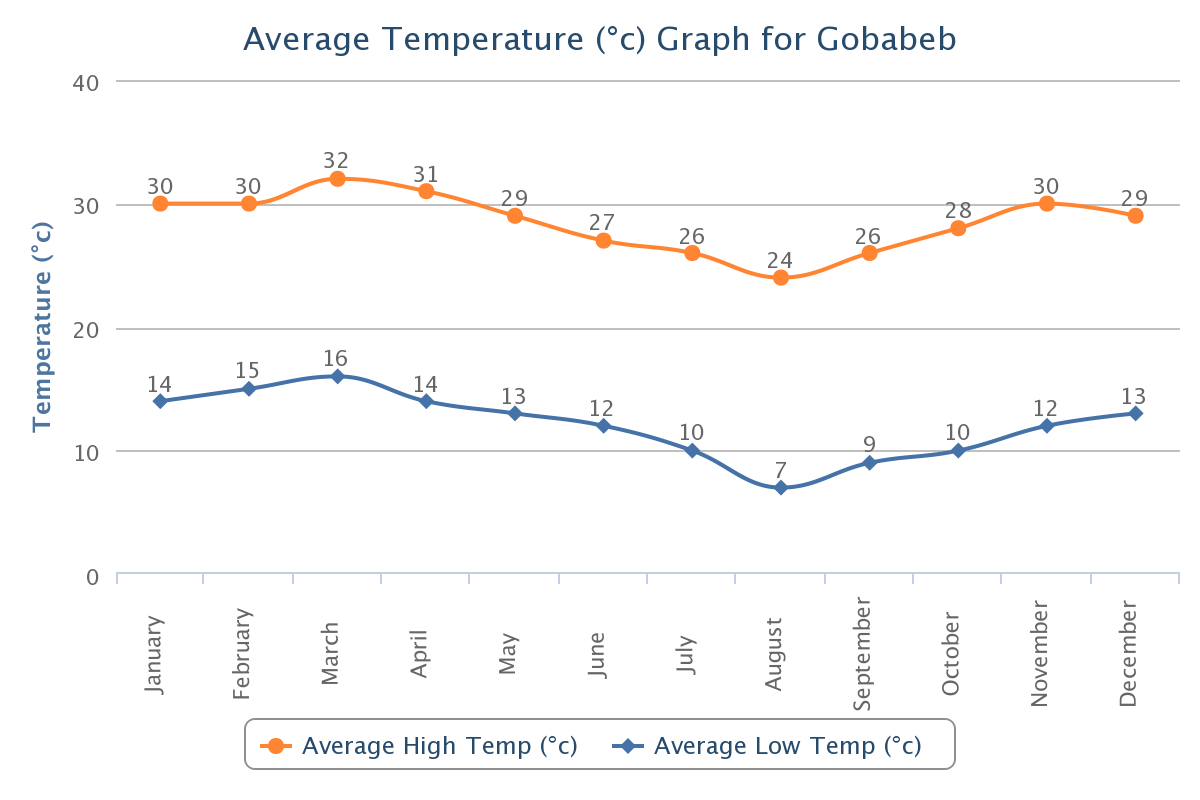

Supplement: Supplementary material 4 — Average annual temperature at Gobabeb [file biodiversity_data_journal-2-e1071-s004.png]

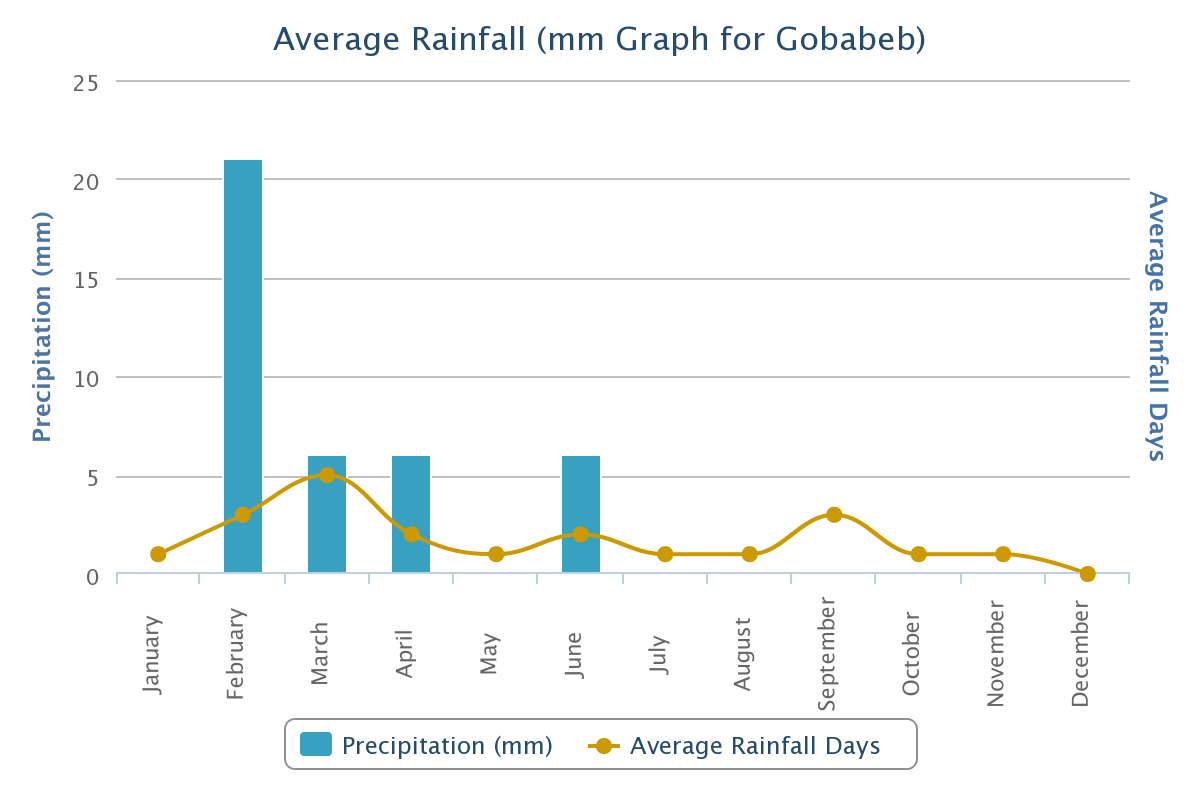

Supplement: Supplementary material 5 — Average annual rainfall at Gobabeb [file biodiversity_data_journal-2-e1071-s005.png]

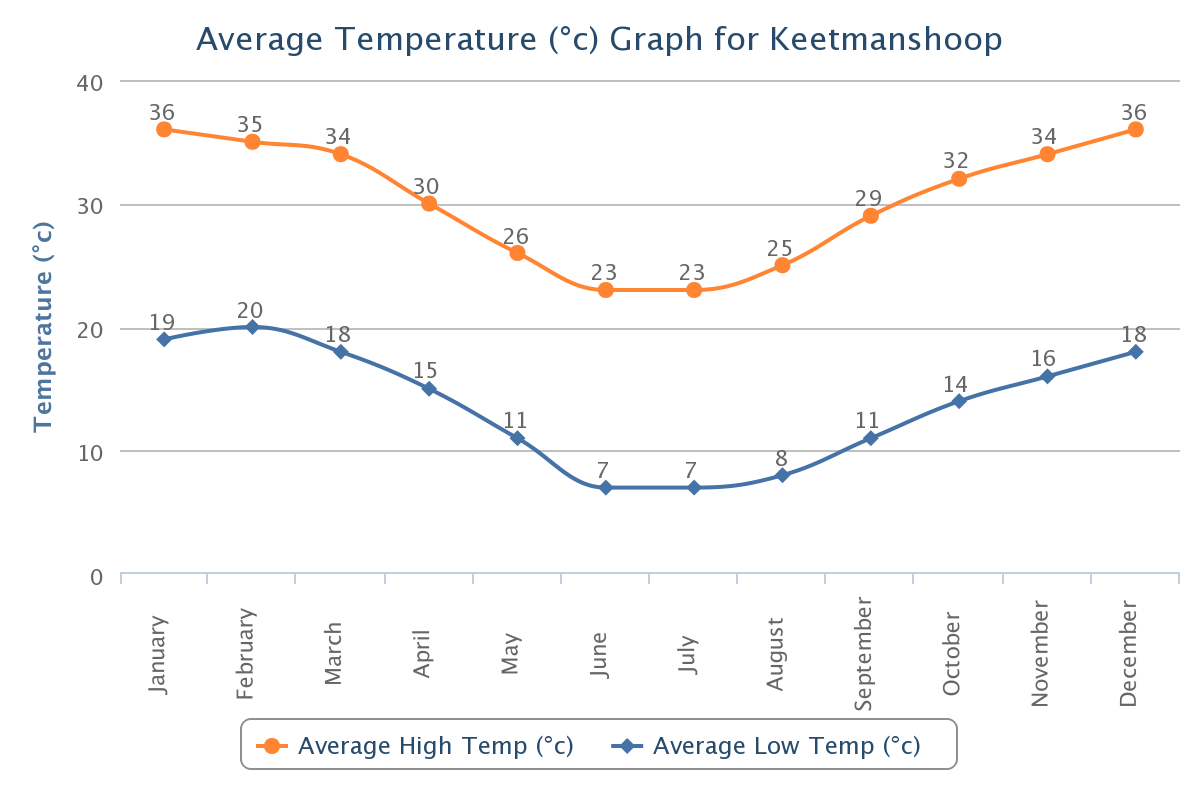

Supplement: Supplementary material 6 — Average annual temperature at Keetmanshoop [file biodiversity_data_journal-2-e1071-s006.png]

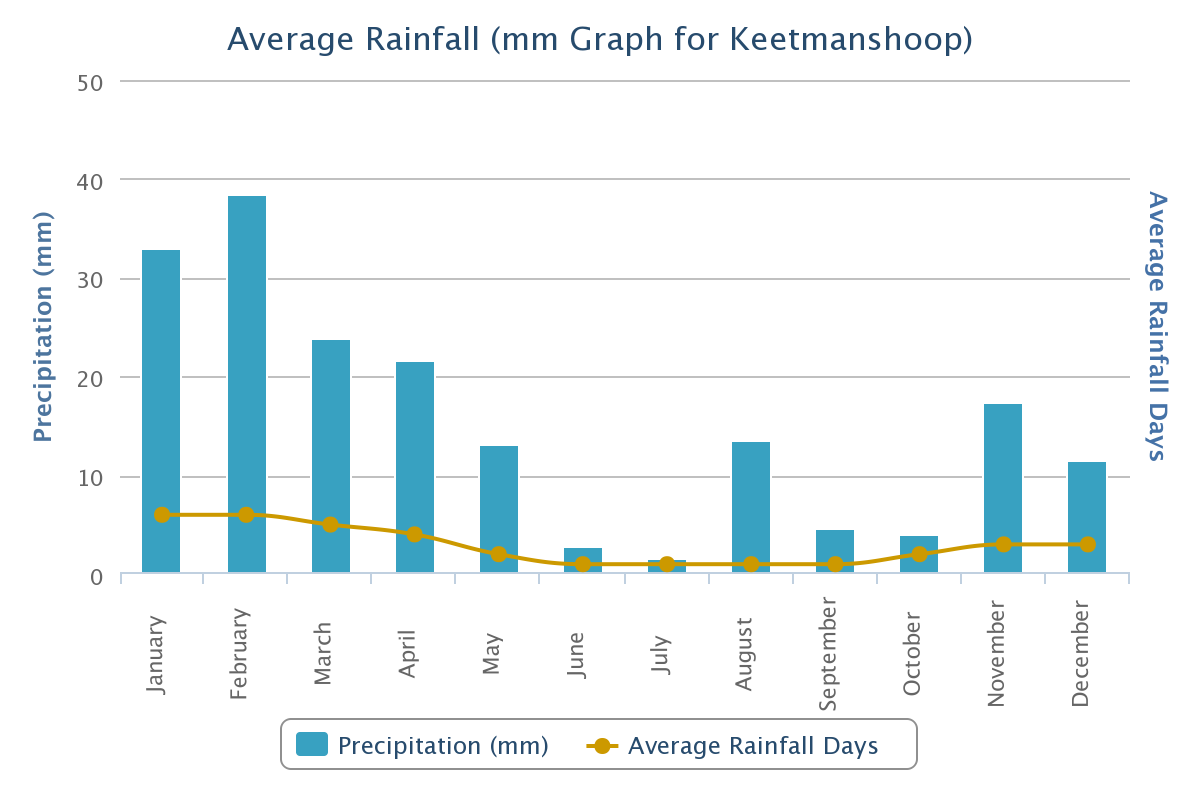

Supplement: Supplementary material 7 — Average annual rainfall at Keetmanshoop [file biodiversity_data_journal-2-e1071-s007.png]

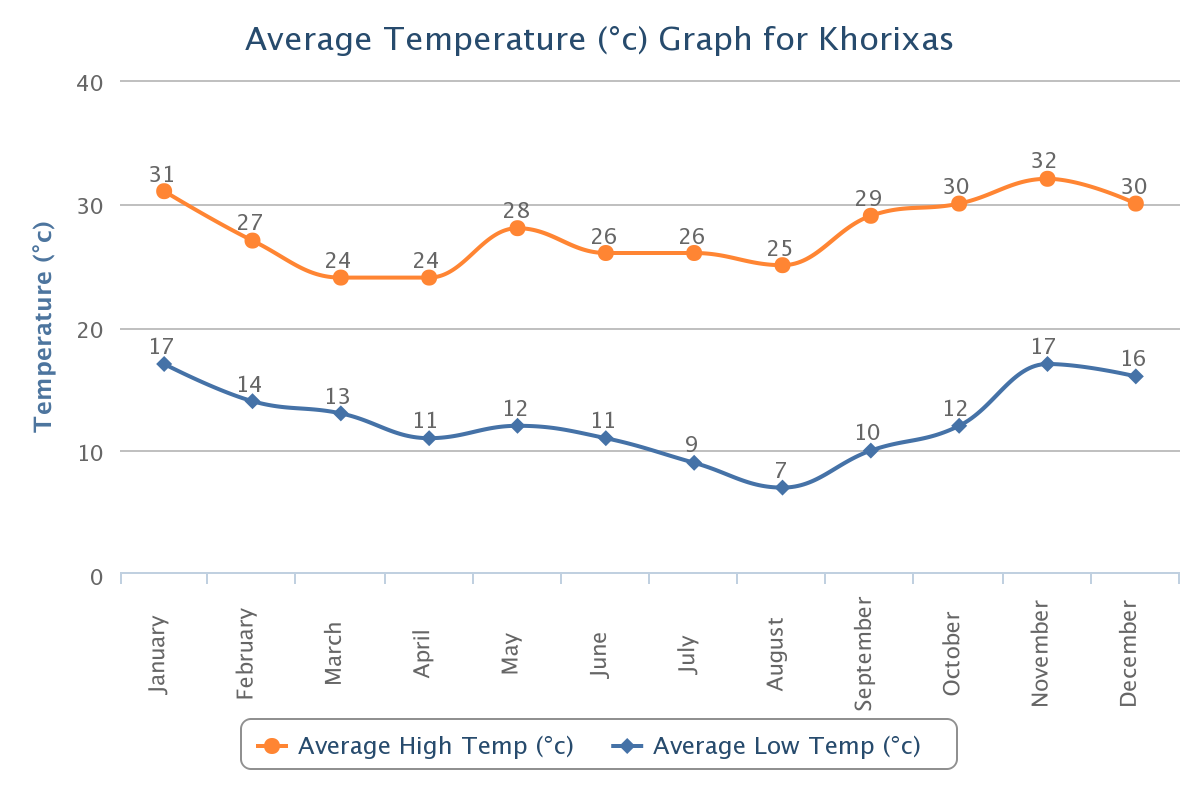

Supplement: Supplementary material 8 — Average annual temperature at Khorixas [file biodiversity_data_journal-2-e1071-s008.png]

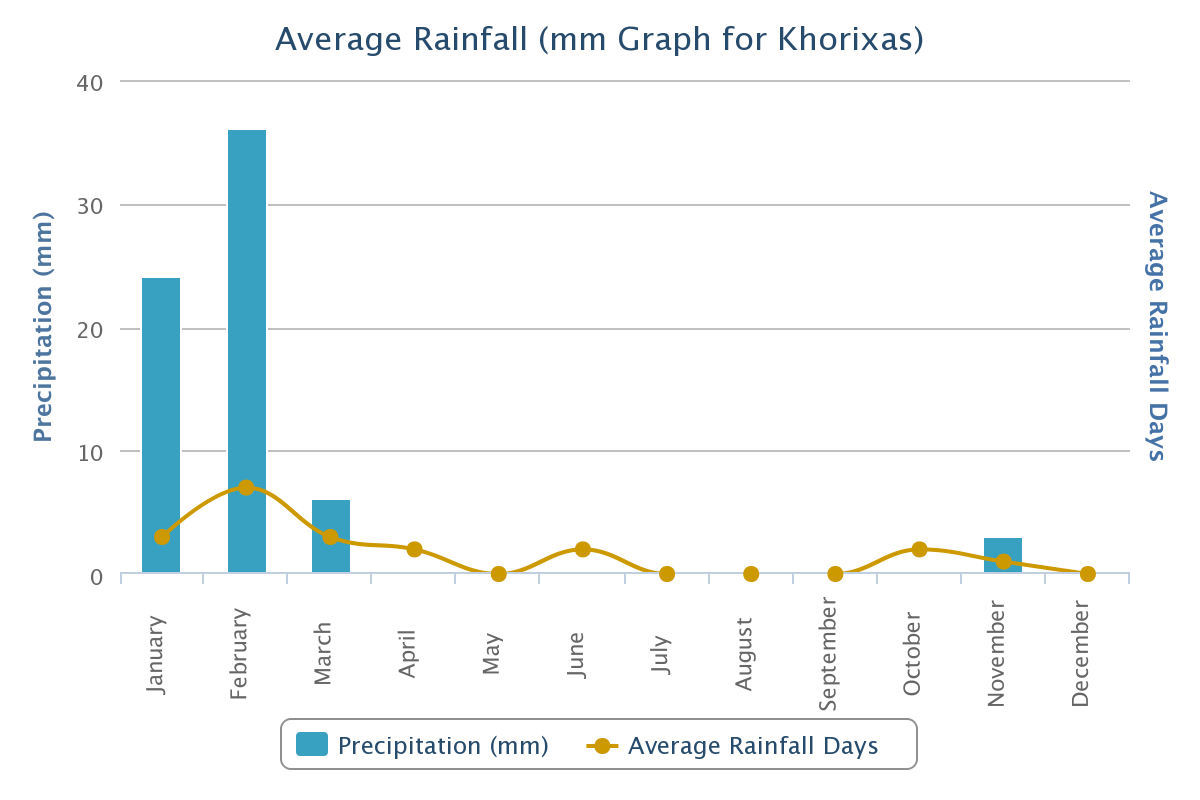

Supplement: Supplementary material 9 — Average annual rainfall at Khorixas [file biodiversity_data_journal-2-e1071-s009.png]

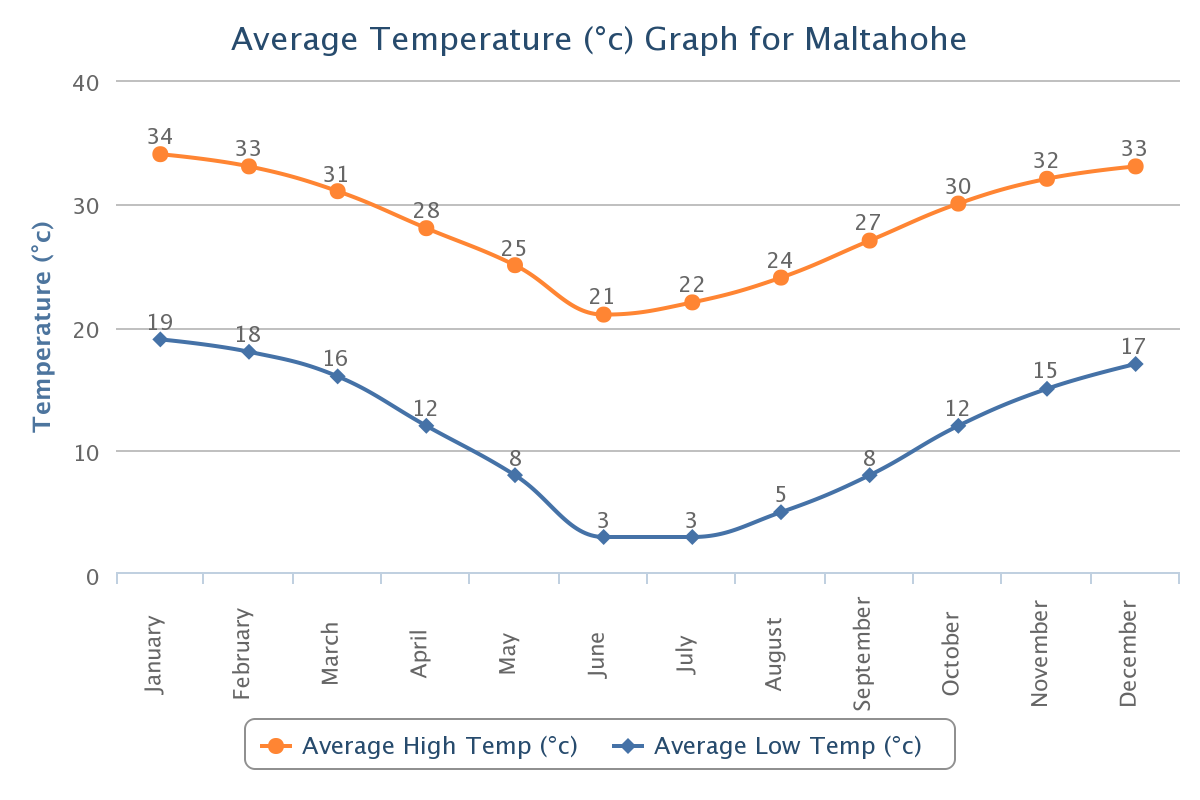

Supplement: Supplementary material 10 — Average annual temperature at Maltahöhe [file biodiversity_data_journal-2-e1071-s010.png]

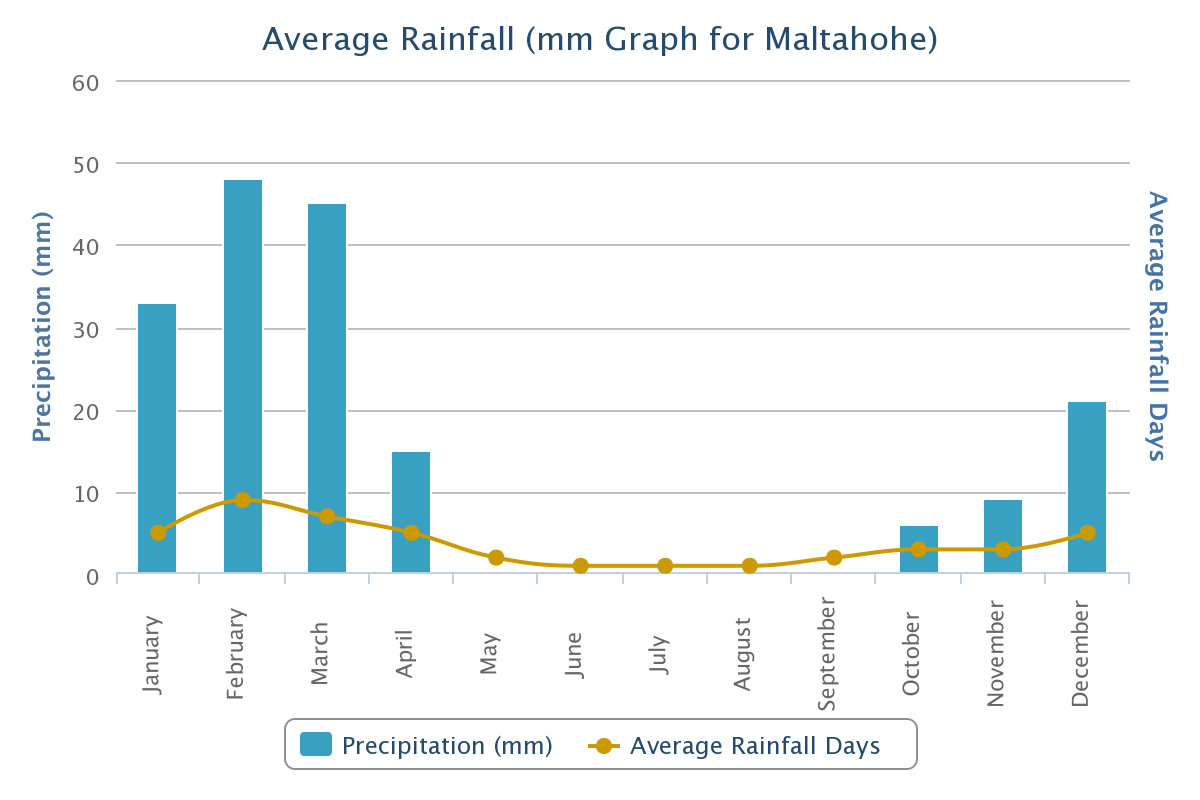

Supplement: Supplementary material 11 — Average annual rainfall at Maltahöhe [file biodiversity_data_journal-2-e1071-s011.png]

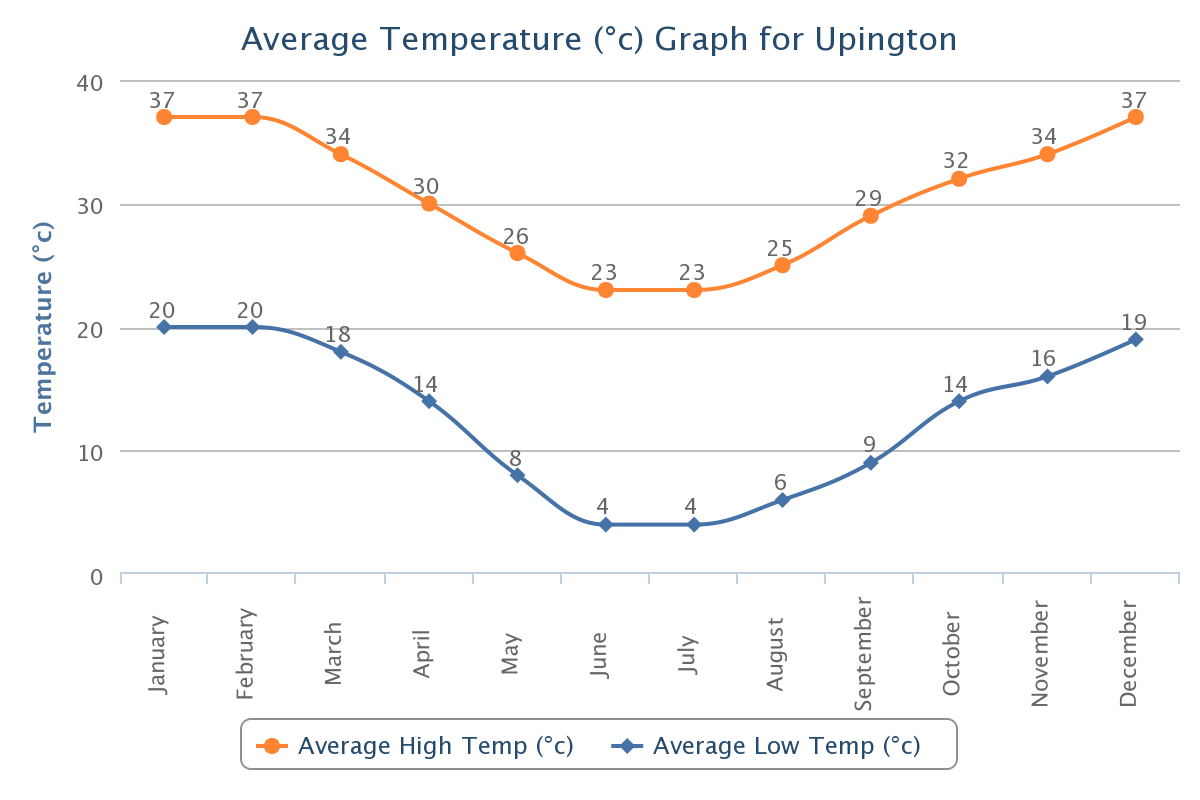

Supplement: Supplementary material 12 — Average annual temperature at Upington [file biodiversity_data_journal-2-e1071-s012.png]

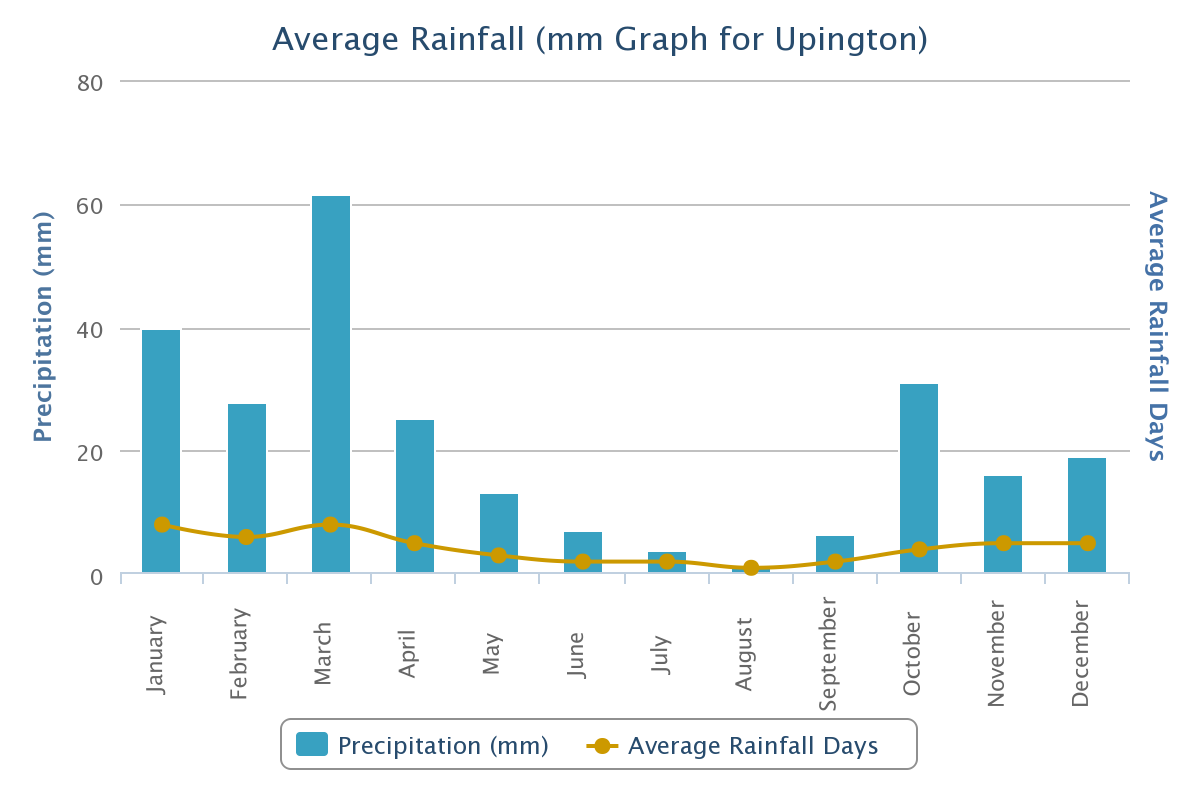

Supplement: Supplementary material 13 — Average annual rainfall at Upington [file biodiversity_data_journal-2-e1071-s013.png]

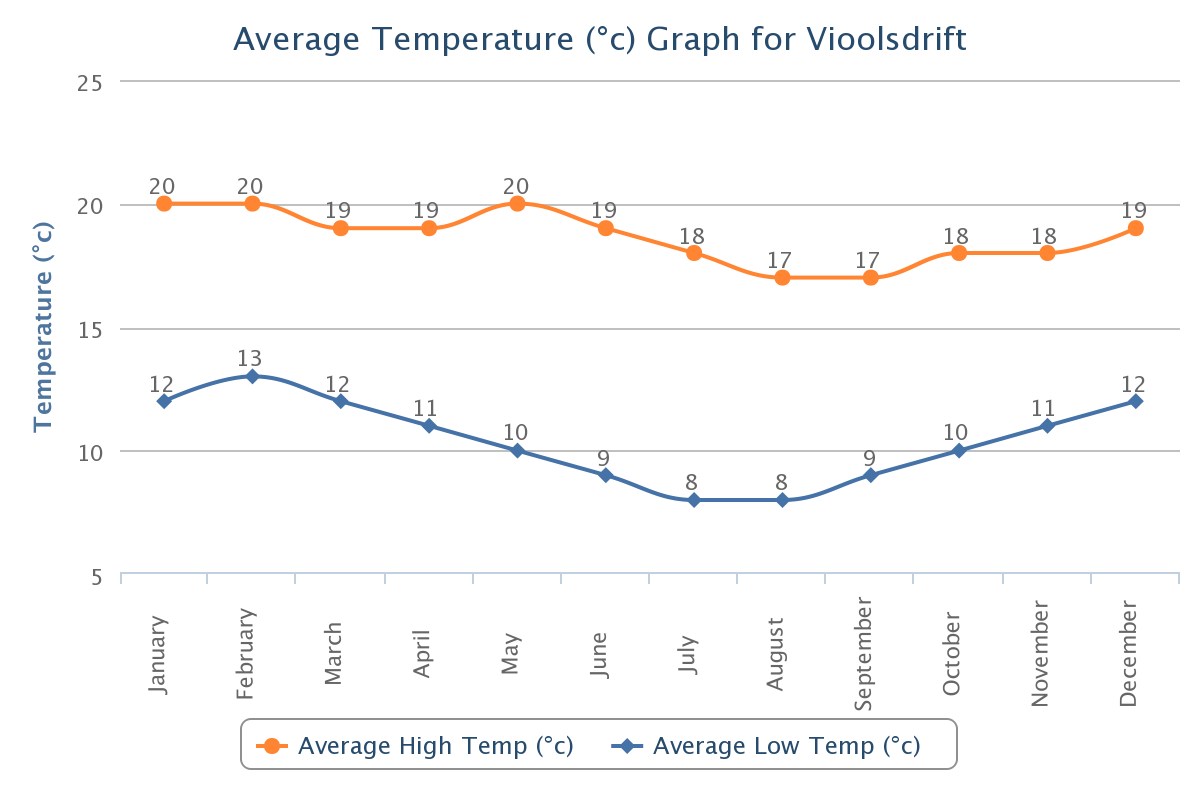

Supplement: Supplementary material 14 — Average annual temperature at Vioolsdrift [file biodiversity_data_journal-2-e1071-s014.png]

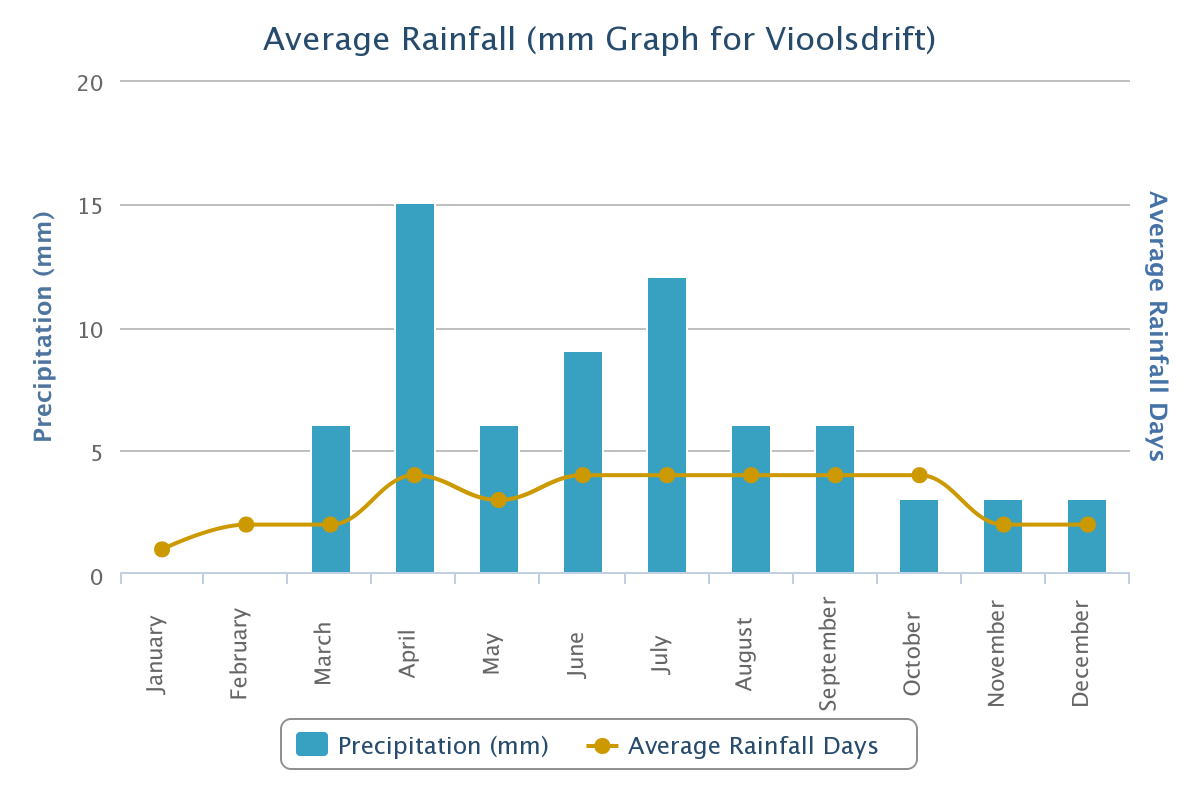

Supplement: Supplementary material 15 — Average annual rainfall at Vioolsdrift [file biodiversity_data_journal-2-e1071-s015.png]
